# Biomaterials-mediated CRISPR/Cas9 delivery: recent challenges and opportunities in gene therapy

**DOI:** 10.3389/fchem.2023.1259435

**Published:** 2023-09-28

**Authors:** Ankit Kumar Dubey, Ebrahim Mostafavi

**Affiliations:** ^1^ Global Research and Publishing Foundation, New Delhi, India; ^2^ Institute of Scholars, Bengaluru, Karnataka, India; ^3^ Stanford Cardiovascular Institute, Stanford University School of Medicine, Stanford, CA, United States; ^4^ Department of Medicine, Stanford University School of Medicine, Stanford, CA, United States

**Keywords:** biomaterials, CRiSPR/Cas, targeted delivery, gene therapy, synthesis

## Abstract

The use of biomaterials in delivering CRISPR/Cas9 for gene therapy in infectious diseases holds tremendous potential. This innovative approach combines the advantages of CRISPR/Cas9 with the protective properties of biomaterials, enabling accurate and efficient gene editing while enhancing safety. Biomaterials play a vital role in shielding CRISPR/Cas9 components, such as lipid nanoparticles or viral vectors, from immunological processes and degradation, extending their effectiveness. By utilizing the flexibility of biomaterials, tailored systems can be designed to address specific genetic diseases, paving the way for personalized therapeutics. Furthermore, this delivery method offers promising avenues in combating viral illnesses by precisely modifying pathogen genomes, and reducing their pathogenicity. Biomaterials facilitate site-specific gene modifications, ensuring effective delivery to infected cells while minimizing off-target effects. However, challenges remain, including optimizing delivery efficiency, reducing off-target effects, ensuring long-term safety, and establishing scalable production techniques. Thorough research, pre-clinical investigations, and rigorous safety evaluations are imperative for successful translation from the laboratory to clinical applications. In this review, we discussed how CRISPR/Cas9 delivery using biomaterials revolutionizes gene therapy and infectious disease treatment, offering precise and safe editing capabilities with the potential to significantly improve human health and quality of life.

## 1 Introduction

Targeted delivery is a major determinant of gene editing’s therapeutic efficacy, and it should be developed to accommodate a range of features, such as the form of the payload, the physiological environment, and potential immune responses. Biomaterials have lately emerged as an intriguing candidate for Cas9 delivery due to their tunability, biocompatibility, and increasing effectiveness (Lino et al., 2018). Though the CRISPR/Cas9 technique is predicted to help a variety of diseases in the long run in an *ex vivo* environment, CRISPR/Cas9 medicines may have greater therapeutic success if they can be supplied directly to patients. *In vivo*, delivery techniques for CRISPR/Cas9 medicinal aspects, on the other hand, are still to be developed (Behr et al., 2021).

The CRISPR/Cas9 complex requires the translocation of its components into the nucleus to work on the nuclear genome. As a result, overcoming tissue and cell membrane barriers is required. CRISPR/Cas9 delivery to specific tissues or cells is difficult and time-consuming (Cheng et al., 2021). Non-viral vectors, viral vectors, and physical delivery are all now used to distribute CRISPR/Cas9. The most common strategy is incorporating CRISPR/Cas9-encoding sequences into the viral genome and then releasing the CRISPR/Cas9 gene complex into infected cells via virus-mediated gene delivery (Xu et al., 2019a). Extrinsic stimuli like magnetic fields, optical radiation, and chemical signals have been used to augment the tissue specificity of systemic CRISPR/Cas9 delivery, allowing for local genome editing. These approaches, however, usually require repeated dosages due to their inability to accomplish therapeutic levels of genome editing at the target location. Furthermore, the challenges of restricting systemic diffusion might lead to considerable genotoxicity and adverse off-target effects (Hsu et al., 2014).

The science of genome editing is intertwined with biomaterials in a complex manner. The goal of early gene editing research was to use biomaterial platforms to disseminate and support genome editing tools so that they might achieve their full potential in gene and cell therapy (Gaj et al., 2016). Simultaneously, gene editing tools and complex biomaterial culture systems were introduced to help us better understand human sickness and cellular function in much more ecological conditions. CRISPR–Cas9 (clustered regularly interspaced short palindromic repeat–CRISPR associated protein) is being used in novel ways to help researchers and manufacturers create revolutionary biomaterial frameworks (Abdeen et al., 2021; Nadakuduti and Enciso-Rodriguez, 2021).

With the aid of the groundbreaking genetic technology CRISPR-Cas9, live creatures’ DNA may be precisely and successfully modified. Because it can target and alter certain DNA sequences, CRISPR-Cas9’s method is based on this capability. The natural defenses of bacteria against viral infections serve as its primary source of inspiration. The system consists of two essential parts: the Cas9 enzyme, which functions as molecular scissors, and the CRISPR array, which is made up of short, repetitive DNA sequences interspersed with distinctive “spacer” sequences generated from previous viral contacts (Lim et al., 2018; Uddin et al., 2020; Nidhi et al., 2021).

The procedure initiates with the transcription of the CRISPR array into precursor molecules, which are subsequently transformed into distinct guide RNAs (gRNAs). Every gRNA is designed to work in conjunction with a particular target DNA sequence. The Cas9 enzyme is attracted to the target location when the gRNA binds to the DNA’s corresponding sequence (Asmamaw and Zawdie, 2021). The targeted place in the DNA is where Cas9 causes a double-strand break, triggering the cell’s inherent repair processes. Subsequently one of two major DNA repair processes, homology-directed repair (HDR) or non-homologous end joining (NHEJ), can occur. Small insertions or deletions (in-dels), frequently occur as a result of NHEJ and may affect the target gene’s functionality. Contrarily, HDR may be used to repair damaged DNA while including a unique DNA template, enabling precise insertion, deletion, or replacement of certain genetic sequences (Kosicki et al., 2022; Fichter et al., 2023; Wang et al., 2023).

**FIGURE 1:**
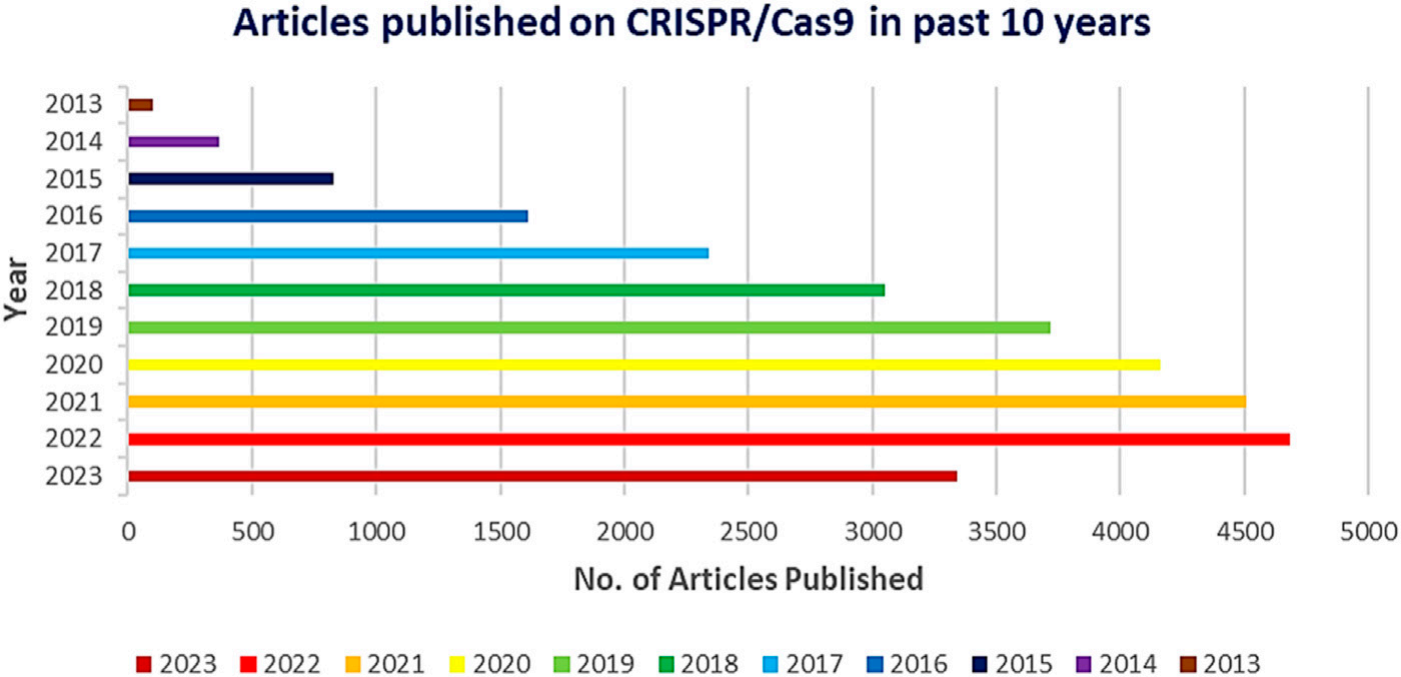
Graph depicting the number of CRISPR/Cas9 related publications yearly by PubMed 2013 to 2023.

Genetic research and applications have been completely transformed by the CRISPR-Cas9 system’s amazing adaptability, use, and effectiveness. With previously unheard-of precision and speed, it has allowed scientists to modify the genomes of a broad variety of creatures, including bacteria, plants, and mammals. But as the technology develops and finds more extensive uses in agriculture, medicine, and other fields, it is still crucial to carefully evaluate ethical issues as well as potential adverse effects (Khwatenge and Nahashon, 2021; Tavakoli et al., 2021). With more than 29,000 papers published in the last 10 years and more than 4,700 in 2022, the CRISPR/Cas9 system was quickly adopted by the scientific community due to its ease of programming and high specificity to perform gene editing at target sites (Figure 1).

In this review, we present a scaffold framework technology for CRISPR/Cas9 genome editing. Conventional biomaterial delivery mechanisms might not have been effective in treating such tissue engineering concerns. We believe the scaffold-mediated delivery mechanism will result in targeted and long-term accessibility of Cas9: sgRNA complexes, which will be useful for genome editing in tissue regeneration and other regenerative medication. Tissue regeneration frequently involves restricted damage that necessitates the restoration of tissue architecture. This study provides a thorough summary of the recent developments, challenges, and prospective opportunities in using biomaterials as CRISPR/Cas9 carriers in gene therapy applications. The relevance of gene therapy and the revolutionary potential of CRISPR/Cas9 technology are discussed at the outset of the paper. The difficulties with successful CRISPR/Cas9 delivery are then discussed, with a focus on the function of biomaterials in resolving these difficulties. A variety of biomaterials, including hydrogels and nanoparticles, are covered in the paper along with how they may be used for gene editing. It also explores the subtleties of *in vivo* and *ex vivo* gene therapy techniques, emphasizing significant advancements and current research. In the end, the study provides a thorough overview of the changing environment of gene therapy that uses biomaterial-based CRISPR/Cas9 delivery, highlighting both the difficulties and bright prospects in this quickly developing field.

## 2 Timeline for the key discoveries in the development of CRISPR/Cas9 in therapeutics

The history of significant discoveries that led to the creation of CRISPR/Cas9 tools that are employed in a variety of therapies is proof of the technology’s fast advancement and game-changing effects. The journey started in the late 1980s and picked up steam in the early 2000s with the discovery of the CRISPR/Cas immune system in bacteria as an adaptive immune system (Ishino et al., 1987). Let’s jump ahead to 2012 when Jennifer Doudna and Emmanuelle Charpentier produced a ground-breaking discovery by proving that the Cas9 protein could be trained using a guide RNA to target particular DNA regions for exact editing (Jinek et al., 2012). This important finding paved the way for a rush of further developments. The CRISPR/Cas9 technology was developed in 2013 by Feng Zhang’s team at the Broad Institute for usage in eukaryotic cells, opening the door for possible uses in human genomes (Ran et al., 2013).

The study on the tool’s ability to fix genetic abnormalities that cause illnesses exploded in the years that followed. In 2015, scientists had already started looking toward therapeutic uses, such as altering T-cells to improve cancer immunotherapies (Zhang and Zhang, 2020). The start of clinical trials employing CRISPR-edited cells for cancer treatment in 2019 highlights the progress made by the technique from the lab to the patient’s bedside. The accuracy and effectiveness of CRISPR/Cas9-based therapeutics were improved as scientists overcame problems including off-target effects and delivery techniques (Rafii et al., 2022). Beginning in the early 2020s, the first *in vivo* human trials for genetic disorders were underway, signaling a substantial advancement toward achieving the therapeutic promise of this ground-breaking technique (Ho et al., 2018). The history of CRISPR/Cas9’s development is a fascinating story of scientific innovation, teamwork, and unrelenting pursuit that has changed the therapeutic landscape and given rise to fresh hope for the treatment of genetic illnesses that were once thought to be incurable (Gostimskaya, 2022). The cumulative successes in the CRISPR/Cas9 area are described in Table 1.

**TABLE 1:** Timeline for the breakthroughs in CRISPR/Cas9 therapeutic development.

Year	Discovery/Event	Description	Reference
1987	Discovery of CRISPR	Japanese researchers describe “clustered regularly interspaced short palindromic repeats” (CRISPR) in *E. coli*	Ishino et al. (1987)
2005	First Evidence of CRISPR Function	Scientists demonstrate that CRISPR sequences play a role in bacterial immunity against phage infections	Barrangou et al. (2007)
2007	Identification of tracrRNA	Researchers discovered trans-activating CRISPR RNA (tracrRNA), which is part of the bacterial immune system	Deltcheva et al. (2011)
2011	CRISPR/Cas9 Genome Editing	Jennifer Doudna and Emmanuelle Charpentier propose using CRISPR/Cas9 for RNA-guided genome editing in bacteria	Jinek et al. (2012)
2012	CRISPR/Cas9 Editing in Eukaryotic Cells	George Church’s lab demonstrates efficient CRISPR/Cas9 genome editing in mammalian cells	Cong et al. (2013); Mali et al. (2013)
2013	Dual RNA Guided Cas9 System	Zhang Feng’s lab published a paper describing the use of the CRISPR/Cas9 system for precise genome editing in eukaryotic cells	Ran et al. (2013)
2014	RNA Editing with Catalytically Dead Cas9	Researchers developed a version of Cas9 with inactivated nuclease activity, known as “dead” Cas9 (dCas9), for RNA targeting and regulation	Hsu et al. (2014)
2015	Base Editing with CRISPR/Cas9	David Liu’s lab introduces base editing, allowing specific nucleotide changes without causing double-strand breaks	Komor et al. (2018)
2016	CRISPR/Cas9 *In Vivo* Gene Editing	Scientists use CRISPR/Cas9 for successful gene editing directly within living animals	Cyranoski (2016)
2017	Clinical Trials for Genetic Disorders	The first clinical trials using CRISPR/Cas9 are initiated to treat genetic disorders like beta-thalassemia and sickle cell anemia	Frangoul et al. (2021); NCT03655678; NCT03745287
2018	Prime Editing	David Liu and Doudna’s labs develop prime editing, a versatile genome editing technique allowing precise insertion, deletion, and substitution	Lapinaite et al. (2020)
2019	*In Vivo*, CRISPR/Cas9 Editing in Humans	Researchers in China report the use of CRISPR/Cas9 to edit genes within the human body for the first time in clinical trials	Lu et al. (2020)
2020	Nobel Prize in Chemistry Awarded	Jennifer Doudna and Emmanuelle Charpentier received the Nobel Prize in Chemistry for the development of CRISPR/Cas9 genome editing	Westermann et al. (2021)
2021	Continued Clinical Trials and Therapeutic Applications	Clinical trials and research continue, exploring CRISPR/Cas9’s potential in treating various genetic and acquired diseases	Li et al. (2020); Liu et al. (2021a); NCT03872479

## 3 Need for biomaterials: advantages over other delivery methods

The inadequacy of delivery approaches is by far the most significant limitation to genetic engineering’s approaching potential. Conventional viral delivery techniques based on retroviruses or adenoviruses have such a significant transfection efficiency, but their inherent cytotoxicity, particularly immune response activation and viral DNA invasion into the recipient chromosome, has limited their utilization (Dubey et al., 2022). Virus vectors have become the usual strategy for *in vivo* delivery, including gene augmentation and genome editing, as the number of gene therapies in clinical trials expands. *In vivo*, testing of viral vectors in disease models such as Duchenne muscular dystrophy, hereditary tyrosinemia type I, and retinitis pigmentosa has shown to be effective. While viral delivery has shown to be quite successful in several carefully selected situations, the limitations of viral delivery continue to be a major roadblock in the clinical translation of various gene therapies (Bulcha et al., 2021).

Because an AAV’s maximum capacity is roughly 4.7 kb, the physical capability of viral vectors is typically a stumbling block for CRISPR delivery. As a result, multiple vectors are typically needed to convey unique features. When specific targeting of an organ system is required, tropism becomes a problem since a single serotype might target multiple physiological functions (Yin et al., 2017; Xu et al., 2019b). Viral vectors also provide specific packaging concerns, such as stabilization at the higher concentration levels necessary for administration, preservation, and storage stability, as well as the cold chain from manufacture to administration (with some of these issues becoming more evident as product manufacturing scales up). In addition, vector preparations differ from batch to batch (Srivastava et al., 2021). Also, the viral capsid (the protein shell encasing viral genetic material) and viral genome could perhaps elicit simultaneous innate and adaptive immune responses, and neutralizing antibodies from previous spontaneous viral infections may exist (Mingozzi and High, 2017). Furthermore, for some viral vectors, the uncontrolled integration of genomic material or the ongoing expression of genome editors following payload delivery might induce disruption of the host genome (Shirley et al., 2020).

**FIGURE 2:**
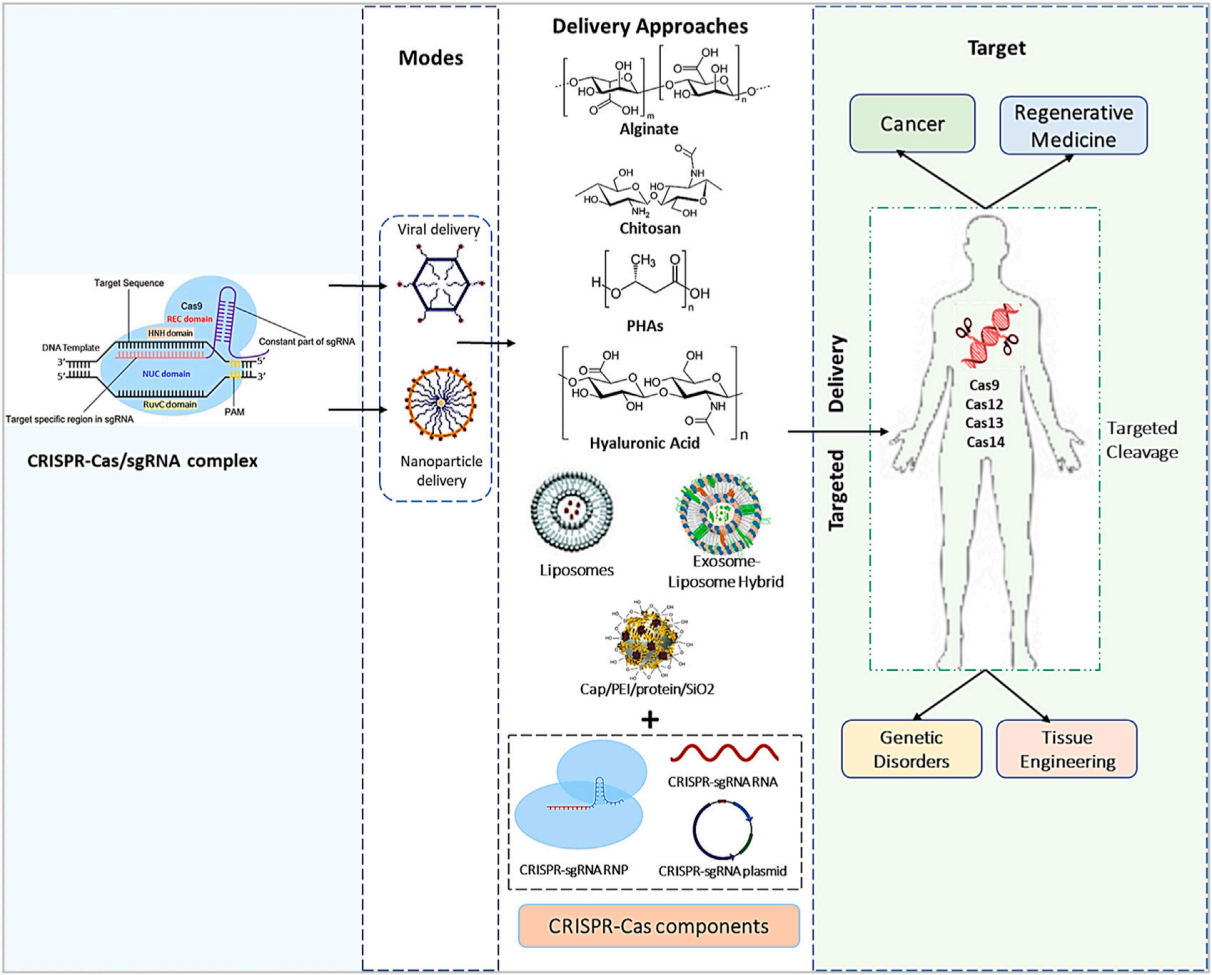
An overview of the CRISPR/Cas9 payload’s genetic engineering processes, effective diagnostics methods, and *in vivo* delivery techniques used in numerous types of medical treatment and medication delivery systems.

By overcoming any of the constraints of viral vectors, the spectrum of possible gene treatments and their effectiveness and safety might be considerably expanded. Furthermore, due to variances in the quantity of DNA, each cell gets as well as cell-to-cell variability in expression levels, controlling the overall dose of Cas9 supplied to each target cell is extremely challenging (Lundstrom, 2018). Gene therapy using synthetic and biological materials as carriers offers an exciting approach that could solve several of these concerns. Biomaterials have traditionally been investigated for use in regenerative medicine administration, despite their uptake into drug development being slower than viral vectors (Figure 2) (Han et al., 2022a). However, given recent success in employing nonviral platforms for vaccines and rising pressures in biomanufacturing viral vectors to fulfill the greater needs for regenerative medicine, current trends show a revived interest in nonviral platforms (Ramamoorth and Narvekar, 2015; Abdeen et al., 2021).

Biomaterials have garnered appeal as non-viral vectors in recent years because of their flexibility, cytocompatibility, and increased transfection effectiveness. Nanoparticles may now be further modified to increase tissue selectivity and nuclear transport, and capacity restrictions are no longer a concern. The capacity of biomaterials to be tuned offers considerable opportunities for enhancing CRISPR–Cas9 delivery for *in vivo* gene editing (Riley and Vermerris, 2017). Biomaterials give a significant edge over physical and viral delivery although they have nearly endless customization opportunities. Different cell types and organ systems will significantly require genome engineering procedures customized to match the requirements of respective vasculature (Mitragotri and Lahann, 2009).

### 3.1 Scaffold-based delivery systems

The development of innovative scaffold architectures and scalable production methods is essential for the advancement of biomedical applications. Biodegradable polymers, such as polylactic acid (PLA), are often employed, however, the final scaffold qualities may be customized depending on how these polymers are treated and what modifiers are added during manufacturing (Song et al., 2018). Even though it would be incredibly practical for patients and physicians if patients’ damaged tissues or organs could be repaired by a straightforward cell injection to a specified place, such occurrences are rare. These conditions include hematopoietic disorders, cardiovascular conditions characterized by capillary or tiny blood arteries dysfunction, such as arterioles, illnesses brought on by a deficiency in physiologically active chemicals, and sensory loss (Ikada, 2006). Most large tissues and organs with distinctive three-dimensional geometries will require aid during the cell-to-organ development process. The support is referred to as an artificial extracellular matrix, or a scaffold (Fuoco et al., 2016; Chooi et al., 2021).

The CRISPR/Cas9 technology, which is versatile and easy to use, can be employed to modify the genome in cell cultures. Delivering the components for genome engineering, especially the Cas9 protein and single-guide RNA (sgRNA), often involves chromosomes, mRNA, or ribonucleoprotein (RNP) complexes. Non-viral methods are especially promising since they overcome the safety concerns presented by viral vectors (Yip, 2020). Regional transport will be facilitated by scaffold-based genome engineering element administration, and it may even result in the development of pharmacological properties that are co-delivered by the same scaffold with other factors like geographic cues. This will allow for cell destiny control for tissue regeneration (Howard et al., 2008).

Researchers developed a scaffold-mediated Cas9 ribonucleoprotein (RNP) delivery method to improve the effectiveness of genetic manipulation in leukemia stem cells (LSCs) in the bone marrow. The *in-vivo* interaction period of Cas9 RNP at the incision site was considerably extended compared to that of free Cas9 RNP by loading Cas9 RNP onto the surface of NF. Due to the direct interaction between medications and cells and the high local drug concentration that scaffold-mediated drug administration offers, *in vitro* genetic modification efficiency is like bulk delivery (Jang et al., 2006; Ho et al., 2021; Zhang et al., 2021a). The interface between host cells and the delivery to target cells would be improved by the surface-coating of the CRISPR/Cas9 complexes. In addition, the complexes would remain at the site of injection for an extended period owing to their adherence to the scaffolding as opposed to slackening off complexes, resulting in a longer-lasting delivery of CRISPR/Cas9 complexes (Wei et al., 2020; Zhang et al., 2021b). Additionally, applying Cas9 RNP/nanoparticle complexes onto a scaffold could shield them from proteolytic deterioration, sustaining the complexes’ higher Cas9 RNP concentration for a longer duration. Similar intracellular absorption and *in vitro* genome-editing effectiveness were observed in Cas9 RNP-loaded NF as well (Chen et al., 2019). For reliable local distribution of the Cas9 RNP, its longer *in vivo* retention duration and lower cytotoxicity would be advantageous.

### 3.2 Scaffold tree delivery

A cutting-edge technique for tissue engineering called scaffold tree delivery integrates the application of scaffolds (biomaterials) with CRISPR/Cas9 technology to administer genetic elements to targeted cells in their natural environment. The CRISPR/Cas9 method enables efficient genetic manipulation in the cells, whereas the scaffold material offers a three-dimensional framework that can assist in the development and differentiation of cells (Pal et al., 2009; Ferracini et al., 2018). A scaffold substance that could promote cellular proliferation and differentiation was created by scientists to practice scaffold tree delivery for synthetic biology. Afterward, researchers manipulated the genes of the cells using CRISPR/Cas9 before being planted into the scaffold. This may entail altering genes to encourage cell division, proliferation, or other desirable characteristics for tissue regeneration (Krishna et al., 2016; Abpeikar et al., 2022). After transformation, the cells are loaded onto the biocompatible substance and implanted into the target tissue location *in vivo*. As they develop and differentiate, cells on the scaffold material release pharmacological components that encourage tissue regeneration and repair. One of the key advantages of scaffold tree management is the ability to accurately control the behavior of the cells used in tissue engineering (Chan and Leong, 2008; Dzobo et al., 2018).

Using CRISPR/Cas9 to modify certain genes, researchers can boost cell activity to facilitate tissue regeneration and repair. The ability to administer genetic materials *in vivo* is yet another benefit of scaffold tree delivery. In other words, the scaffold material can be surgically placed into the desired tissue location, where it can release therapeutic elements over time (Hsu et al., 2014; Hazafa et al., 2020). The intriguing new method of scaffold tree delivery for tissue engineering combines the advantages of scaffold materials with the accuracy of CRISPR/Cas9 gene editing. The technique will probably be used in novel ways throughout time to develop functioning tissues and organs for a variety of purposes (Chooi et al., 2021).

It has been suggested that using the gene that codes for a growth factor to target cells is an effective way to enable constant interpretation and release of the growth factor in the host tissue forum and prevent complications with expression levels that can arise during the challenging process of formulation and the brief half-life after discharge in the biological fluids when growth factor-releasing scaffolds are used (Yun et al., 2010; Robubi et al., 2014). Transfecting seeded cells and expressing the growth factor to encourage the morphogenesis of specific cells to produce the desired tissue, the genetic material is continuously delivered via polymeric scaffolds, either as bare DNA or in the form of polyplexes (Hwang et al., 2010; Muzzio et al., 2021).

In recombinant DNA technology, the effectiveness of delivering Cas9: sgRNA complexes utilizing fiber scaffolds was investigated. To validate the efficacy of the experimental scaffold’s genome editing, researchers employed prototype cultured cells U2OS, which contains individual clones of the enhanced fluorescent green protein (EFGP) inserted into the genome (Chin et al., 2019). A sgRNA was created specifically to target this EGFP gene. They used a bio-adhesive covering called poly-DOPA-melanin (pDOPA), which was inspired by mussels, to make it easier for Cas9: sgRNA complexes to adhere to the fiber scaffolds (Xie et al., 2012). They show that effective genetic manipulation utilizing these scaffolds is possible by inverse transfection. All such systems could make it easier to translate genome editing in the future for applications like tissue regeneration (Lawhorn et al., 2014; Iwasaki et al., 2020).

#### 3.2.1 The potential of CRISPR/Cas9 in tissue engineering

Using the precise genome editing that CRISPR/Cas9 technology enables in cells used for tissue regeneration, tissue engineering has the potential to undergo a revolution. Using biomaterials and cells, tissue engineering aims to produce functioning tissues or organs that may take the place of the body’s sick or damaged tissues (Armstrong and Stevens, 2019). Getting a firm grip on the behavior of the cells utilized in the regeneration process is one of the main difficulties in tissue engineering. Scientists can accurately change DNA sequences in cells using the potent genome editing technology CRISPR/Cas9 (Gopal et al., 2020). In tissue engineering cells, certain genes may be altered using CRISPR/Cas9 to improve their capacity for regeneration, encourage differentiation into cell types, and optimize their behavior in response to signals. For instance, CRISPR/Cas9 might be used to change the genes that regulate stem cells’ differentiation and proliferation, which are frequently employed in tissue engineering applications (Zhang et al., 2017; Valenti et al., 2019). Scientists might influence the development of stem cells into cell types, such as bone or cartilage cells, that may be utilized to rebuild damaged tissues by manipulating the expression of these genes. Moreover, CRISPR/Cas9 has the potential to alter immune response-related genes, which could enhance the compatibility of transplanted tissues with the recipient’s immune system. This could lower the possibility of rejection and increase the effectiveness of tissue engineering treatments over time (Clément et al., 2017; Ryczek et al., 2021; Meissner et al., 2022).

Tissue engineering using CRISPR/Cas9 involves using genome editing to modify the genetic makeup of cells used for tissue regeneration. The goal is to enhance the regenerative potential of these cells and optimize their behavior in response to specific signals. One example of how CRISPR/Cas9 can be used in tissue engineering is in the development of organoids, which are three-dimensional structures that mimic the architecture and function of organs in the body (Driehuis and Clevers, 2017; Rodríguez-Rodríguez et al., 2019). By modifying specific genes in stem cells, researchers can direct their differentiation into specific cell types that can be used to create organoids that closely resemble real organs. In addition to organoid development, CRISPR/Cas9 can also be used to modify genes involved in immune responses. This could improve the compatibility of transplanted tissues with the recipient’s immune system, reducing the risk of rejection and improving the long-term success of tissue engineering procedures (Yin et al., 2016; Lehmann et al., 2019; Kim et al., 2020). CRISPR/Cas9 can also be used to modify genes involved in wound healing, such as those that control the migration and proliferation of cells. By enhancing the ability of cells to regenerate damaged tissues, CRISPR/Cas9 could improve the outcomes of tissue engineering procedures for injuries and diseases (Grath and Dai, 2019; Srifa et al., 2020; Li et al., 2023a).

Using CRISPR/Cas9 to change certain genes or regulatory components, it is possible to precisely design tissues. This can lessen the possibility of negative effects while improving the efficacy of created tissues. By altering the expression of genes involved in angiogenesis, CRISPR/Cas9 has been utilized, for instance, to create a functioning blood artery (Liu et al., 2019; Uddin et al., 2020). With the introduction of disease-causing mutations into cells, CRISPR/Cas9 may be used to simulate illnesses in a lab setting. This can be utilized to research the processes that underlie the development of diseases and to create novel treatments. For instance, the modeling of Huntington’s disease using CRISPR/Cas9 in stem cells has resulted in the discovery of novel therapeutic targets (Cai et al., 2016; Cribbs and Perera, 2017; Li et al., 2020). Gene-edited tissues may be generated using CRISPR/Cas9 and exploited for drug testing or transplantation. For instance, CRISPR/Cas9 was utilized to make gene-edited liver tissue that might be used to study drug metabolism and liver disease (Xu et al., 2020a). CRISPR/Cas9 technology can be used to change the genomes of immune and stem cells used in tissue engineering to improve their therapeutic potential. For instance, T cells may be modified using CRISPR/Cas9 to form chimeric antigen receptors (CARs), which can enhance their ability to detect and destroy cancer cells (Gao et al., 2019; Kim et al., 2021). Overall, the potential of CRISPR/Cas9 in tissue engineering is vast, and ongoing research is likely to uncover new ways to harness this powerful technology to create functional tissues and organs that can be used to treat a wide range of diseases and injuries.

### 3.3 Delivery approaches for CRISPR/Cas9 gene editing techniques

By enabling researchers to precisely alter an organism’s DNA, CRISPR/Cas9 gene editing techniques have revolutionized genetic engineering and biotechnology. An essential step in gene editing is the introduction of CRISPR/Cas9 components into target cells (Li et al., 2023b). The target cell type needed editing efficiency, the likelihood of off-target effects, and whether the editing is carried out *in vitro* or *in vivo* all play a role in determining the delivery approach. To improve the accuracy and effectiveness of CRISPR/Cas9 gene editing techniques, researchers are always looking into and inventing new delivery systems (Tycko et al., 2016; Naeem et al., 2020). There are several approaches for delivering CRISPR/Cas9 systems, each with its advantages and limitations.

i. Viral Vectors: Delivering CRISPR/Cas9 components is frequently done via viral vectors. There are lentiviruses, adenoviruses, and adeno-associated viruses (AAVs) among them. These viruses contain the Cas9 gene and guide RNA sequences that are designed to enter target cells (Asmamaw Mengstie, 2022). Although viral vectors are effective in delivering drugs to a variety of cell types, they may have drawbacks such as immunogenicity, the possibility for haphazard integration into the host genome, and a small cargo capacity (Bulcha et al., 2021).
ii. Plasmid DNA Transfection: Cells can be transfected using methods like electroporation or lipofection using plasmid DNA carrying the Cas9 gene and guiding RNA sequences. Plasmid-based delivery is easy and affordable, but it may not be as successful as viral approaches, especially in non-dividing cells (Chong et al., 2021).
iii. Ribonucleoprotein (RNP) Delivery: In this method, the Cas9 protein and guide RNA are already put together and are given to the cells as a ribonucleoprotein complex. Given that the components of RNP delivery are ephemeral and not incorporated into the genome, it provides great editing efficiency and lowers off-target consequences. But to ensure effective delivery, it must be optimized, and some cell types may not respond as well (Jiang and Doudna, 2017; Zhang et al., 2021c).
iv. Electroporation: To allow CRISPR/Cas9 components to enter cells, electroporation entails delivering electrical pulses to temporarily break apart the cell membrane (Sinclair et al., 2023). Nucleic acids may be introduced into a variety of cells, including those that are challenging to transfect, using this standard technique. However various cell types need to be optimized for electroporation since it might be hazardous to them (Fus-Kujawa et al., 2021).
v. Lipid Nanoparticles (LNPs): To effectively transfer CRISPR/Cas9 components into cells, lipid-based nanoparticles may be loaded. LNPs may be made for certain cell types and are very simple to develop. For *in vivo* delivery functions, are being investigated (Jung et al., 2022).
vi. Protein Engineering: Cas9 proteins are being changed by researchers to make them easier to distribute. For instance, basic editors, high-fidelity Cas9, and smaller Cas9 variations like Cas9 nickase may all be administered more effectively and with fewer off-target consequences (Xu et al., 2019a).
vii. Cell-Penetrating Peptides (CPPs): CPPs are short peptides that can make it easier for substances to enter cells. To increase their absorption by cells, they can be combined with Cas9 or guide RNAs (Zhang et al., 2021d).
viii. Microinjection: Direct insertion of CRISPR/Cas9 components into cells is possible for some applications, such as editing embryos, using microinjection. Specialized tools and knowledge are needed for this technique (Khalil, 2020).

### 3.4 Biopolymer-based delivery system for CRISPR/Cas9

Biopolymer-based delivery systems are a particular kind of drug carrier technique that employs biodegradable polymers, such as proteins, polysaccharides, and lipids, as carriers for medications or beneficial substances. Because of these systems’ bioactivity, good biocompatibility, and capacity to target certain cell types and tissues, interest in them has grown over the past few years (Nitta and Numata, 2013; Gheorghita et al., 2021). Secondary metabolites, proteins, nucleic acids, and vaccines may all be delivered using biopolymer-based delivery methods, as well as a variety of other medications and bioactive substances. Moreover, they may be made to release the medicine or bioactive substance gradually, enabling sustained release over a long period (Hong et al., 2020; Moeini et al., 2022). Targeting certain tissues and cells allows biopolymer-based delivery systems to increase therapeutic effectiveness while minimizing negative effects. Moreover, gene therapy, tissue engineering, and regenerative medicine might all benefit from their use (Xia et al., 2021). Some of the most used biopolymers in drug delivery systems include chitosan, alginate, collagen, and gelatin. These biopolymers can be modified to improve their physical and chemical properties, such as their stability, solubility, and drug release profile (Dumontel et al., 2023).

Although research on CRISPR/Cas9 delivery methods based on biopolymers continues to be in its initial phases, there have been a few encouraging findings. For instance, a paper that was released described the creation of a CRISPR/Cas9 nanoparticle delivery system based on chitosan. It was demonstrated that the technique efficiently delivered the CRISPR/Cas9 components to human lung cancer cells *in vitro*, leading to substantial gene editing (Duan et al., 2021a; Nie et al., 2022). Another study detailed the creation of a CRISPR/Cas9 delivery platform based on silk fibroin. It was demonstrated that the method successfully delivered the CRISPR/Cas9 elements to cells derived from human breast cancer *in vitro*, leading to considerable genetic manipulation (Kaushik et al., 2019; Baci et al., 2021).

#### 3.4.1 Chitosan

A cationic polymer derived from chitin called chitosan has undergone substantial research as a drug carrier medium. It can create microparticles, hydrogels, and nanoparticles that may be utilized to deliver a range of medications, including proteins, peptides, and DNA (Cheung et al., 2015). Delivery methods based on chitosan have been utilized to treat infections, inflammatory disorders, and cancer. For instance, curcumin, a naturally occurring anti-cancer chemical, had its bioavailability and anti-tumor activity increased by a chitosan-based nanoparticle system (Huang et al., 2017). Because of their mucoadhesive qualities and capacity to improve drug absorption, chitosan-based delivery systems have shown promise in the administration of many medications, including anti-cancer therapies. Chitosan-based delivery systems for a variety of medicines, including polypeptides, polymers, and polynucleotides, have been created (Mohammed et al., 2017; Herdiana et al., 2021a). The effective creation of chitosan-based nanocarriers for the transport of capsaicin, an aqueous solubility anti-inflammatory medication. Chitosan has also been explored for gene delivery, with results demonstrating that it has a higher transfection effectiveness than other delivery methods (Cao et al., 2019; Herdiana et al., 2021b).

Nucleic acids and other medicinal compounds, including those, have been thoroughly explored for use as delivery systems using chitosan. Delivering CRISPR/Cas9 gene editing tools is one of the chitosan’s most intriguing uses (Caprifico et al., 2022). With the use of the potent gene-editing tool CRISPR/Cas9, genes in the genome may be precisely targeted and altered. Nevertheless, because the CRISPR/Cas9 components are bulky and negatively charged, they have a difficult time crossing the cell membrane, making their distribution into target cells a significant barrier (Qiao et al., 2019; Khademi et al., 2022). It has been demonstrated that chitosan-based delivery methods may successfully overcome this obstacle and distribute CRISPR/Cas9 into target cells (Wilbie et al., 2019).

**FIGURE 3:**
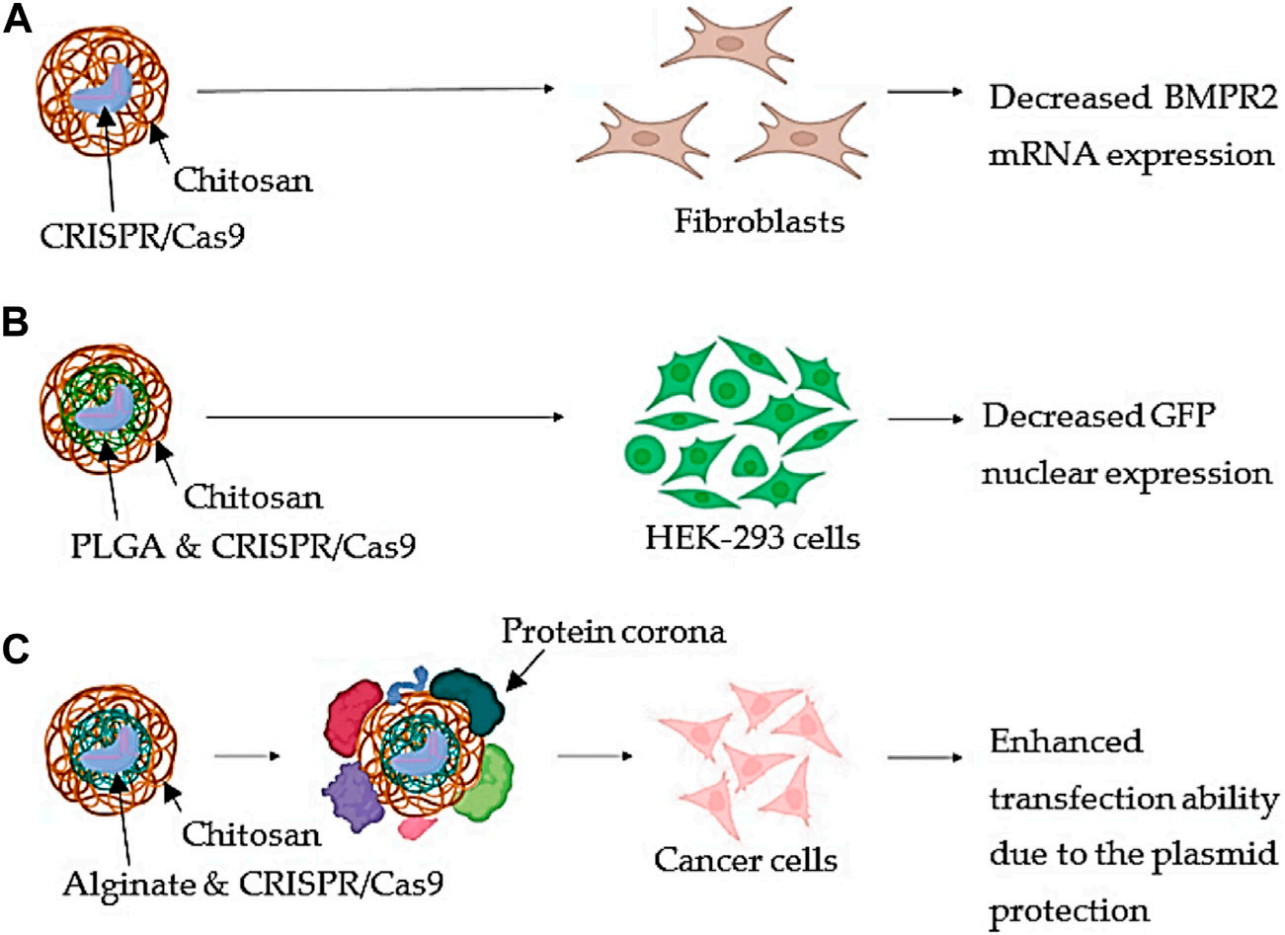
Chitosan-based tactics with immaculate backbone. **(A)** Using pristine CsNPs, fibroblasts were transfected to treat pulmonary arterial hypertension. The major gene, BMPR2, which is implicated in the condition, had its mRNA expression reduced as a consequence. **(B)** PLGA NPs complexed with CRISPR/Cas9 are coated with chitosan, and HEK-293 cells transfected with these particles revealed reduced GFP nuclear expression. **(C)** Chitosan is employed as a coating material for alginate nanoparticles that have been combined with CRISPR/Cas9. After the protein corona formed, treatment of cancer cells revealed an improved transfection capacity because the plasmid was protected from enzymatic destruction (Adapted from Caprifico et al., 2022).

Chitosan is often utilized as a cationic polymer to bind with the negatively charged CRISPR/Cas9 components in chitosan-based CRISPR/Cas9 delivery systems, which mainly include the creation of nanoparticles. Cells may quickly absorb the resultant nanoparticles and transfer them to the nucleus, where the CRISPR/Cas9 components can carry out their gene-editing task (Givens et al., 2018; Zhang et al., 2018; Rouatbi et al., 2022). The CRISPR/Cas9 combination was loaded with CsNPs by researchers to treat pulmonary arterial hypertension (PAH). The bone morphogenic protein receptor II (BMPR2) gene was proposed as the key gene implicated. BMPR2 displays functional alterations or decreased expression, which causes lung cells to proliferate less and undergo more apoptosis (Figure 3A). As a result, fibroblasts were used for the CRISPR/Cas9 transfection for altering BMPR2. The findings suggested that utilizing pure CsNPs for transfection of cells resulted in a considerable reduction in BMPR2 mRNA expression and an increase in cell proliferation. Site-specific and regulated distribution might be enabled accessible by encapsulating the surface of PLGA NPs with chitosan in order to enhance electrostatic attraction with the negatively charged membranes of the cells (Figure 3B). Additionally, using the CRISPR/Cas9 plasmid distribution technique for addressing the human embryonic kidney cell line (HEK-293), the replication of green fluorescent protein (GFP) was 80% suppressed. Even though the formation of protein corona was induced because the size of NCs increased by 1.3-fold and their transfection ability by about 20% in several cancer cell lines, the coating of AGNPs with Cs conferred a higher protection of the plasmid in the presence of serum proteins (Figure 3C). This was because the coating protected the plasmid from enzymatic degradation. (Tuder et al., 2009; Caprifico et al., 2022; Kabwe et al., 2022).

Numerous studies have shown that CRISPR/Cas9 delivery methods based on chitosan are efficient in a variety of cell types and animal models. For instance, chitosan nanoparticles may effectively transfer CRISPR/Cas9 to human lung cancer cells, which resulted in a substantial decrease in cancer cell viability (Xu et al., 2021; Huang et al., 2022a). Several formulations and preparation techniques have been used to create chitosan-based delivery systems for CRISPR/Cas9. One method uses chitosan nanoparticles, which may be created by ionic gelation, polyelectrolyte complexation, or coacervation techniques. To increase these nanoparticles’ stability, cellular absorption, and gene editing effectiveness, targeted ligands, and surface modifications can be added (Vauthier et al., 2013; Ghadi et al., 2014). Chitosan hydrogels or films, which may be loaded with CRISPR/Cas9 components and delivered directly to target tissues or cells, are a different strategy. These formulations can enhance the bioavailability and retention of the gene editing components and offer a prolonged release of those components (Mahmudi et al., 2022).

#### 3.4.2 Alginate

Another biopolymer that has been extensively employed in drug delivery applications is alginate. Drugs including antibiotics, anti-inflammatories, and growth hormones have all been delivered using hydrogels made of alginate. Applications for tissue engineering, such as the regeneration of bone and cartilage, have also made use of alginate-based systems (Lee and Mooney, 2012; Abourehab et al., 2022). There have been many studies on the use of alginate-based delivery techniques in biochemical regeneration and cellular encapsulation. Alginate has also been used to administer medications, and the results point to a delayed drug release and greater therapeutic efficacy (Zhang et al., 2021a). Alginate-based delivery systems for a range of pharmaceuticals, including proteins, peptides, and small molecules, have been developed. Making hydrogels with an alginate base that work well to deliver insulin continuously (Li and Mooney, 2016; Tomić et al., 2023).

These methods are being investigated for a variety of applications, such as skin regeneration, cancer treatment, and genetic editing. To enclose and distribute CRISPR/Cas9 components, such as plasmid DNA or ribonucleoproteins (RNPs), to target cells, alginate-based delivery approaches have been utilized (Wang et al., 2022). According to one study, the delivery of CRISPR/Cas9 RNPs to lung cancer patient cells via an alginate-based delivery method led to precise genetic alterations. An alginate-based polymer may be able to carry CRISPR/Cas9 RNPs to a murine model’s retina, enabling precise genetic manipulation and a possible cure for retinal illnesses, according to another study. Target cells may be effectively supplied utilizing alginate-based nanoparticles and CRISPR/Cas9 (Burnight et al., 2018; Behr et al., 2021; Caprifico et al., 2022).

The research discovered that the CRISPR/Cas9 system could be adequately delivered to targeted *in vitro* cultivating cells using alginate nanoparticles that were loaded with the Cas9 protein and sgRNA (single guide RNA) (Wan and Ping, 2021). In research that has been released, scientists have demonstrated that alginate hydrogels can increase the efficacy of CRISPR/Cas9 administration by halting the degradation of Cas9 protein and sgRNA by restriction enzymes in plasma. For specific applications, CRISPR/Cas9 delivery on alginate may be tailored (Guo et al., 2018; Farheen et al., 2022). In a study, researchers modified the alginate nanoparticles’ surface using a “click” chemical technique to better target and distribute CRISPR/Cas9 to certain cell types. Investigations on CRISPR/Cas9 delivery utilizing alginate have been conducted on animals (Alallam et al., 2020). In a recent study, it was shown that alginate nanoparticles loaded with the Cas9 protein and sgRNA could effectively transport the CRISPR/Cas9 system to target cells in the lungs of mice (Kim et al., 2020). Overall, these investigations indicate that alginate-based delivery methods may be useful for CRISPR/Cas9 gene editing applications, but more study is required to tailor the delivery system for uses and assess its effectiveness and safety *in vivo*.

#### 3.4.3 Collagen

A natural protein called collagen is present in large amounts in the extracellular environment of several tissues, including cartilage, skin, and bone. Because of its biological properties and capacity to promote tissue repair and cell proliferation, it has been utilized in the creation of systems for delivering drugs. Small compounds, peptides, and growth factors have all been delivered using collagen-based methods (Gelse et al., 2003; Dong and Lv, 2016; Naomi et al., 2021). Collagen-based delivery methods have been employed by investigators to successfully deliver CRISPR/Cas9 to target cells *in vitro* and *in vivo*. In one research, CRISPR/Cas9 was successfully delivered via a collagen-based delivery method to treat mice with Duchenne muscular dystrophy (Sharma et al., 2021; Javaid et al., 2022). Another research demonstrates that CRISPR/Cas9 may be delivered to neuronal cells via a collagen-based delivery method, possibly leading to novel treatment options for neurological illnesses (Abdelnour et al., 2021). Collagen-based delivery methods have also demonstrated potential in lowering CRISPR/Cas9 off-target effects, a significant problem in gene therapy. The development of collagen-based delivery methods for the effective and targeted administration of CRISPR/Cas9 to target cells still faces obstacles (Maeder and Gersbach, 2016; Li et al., 2022a).

**FIGURE 4:**
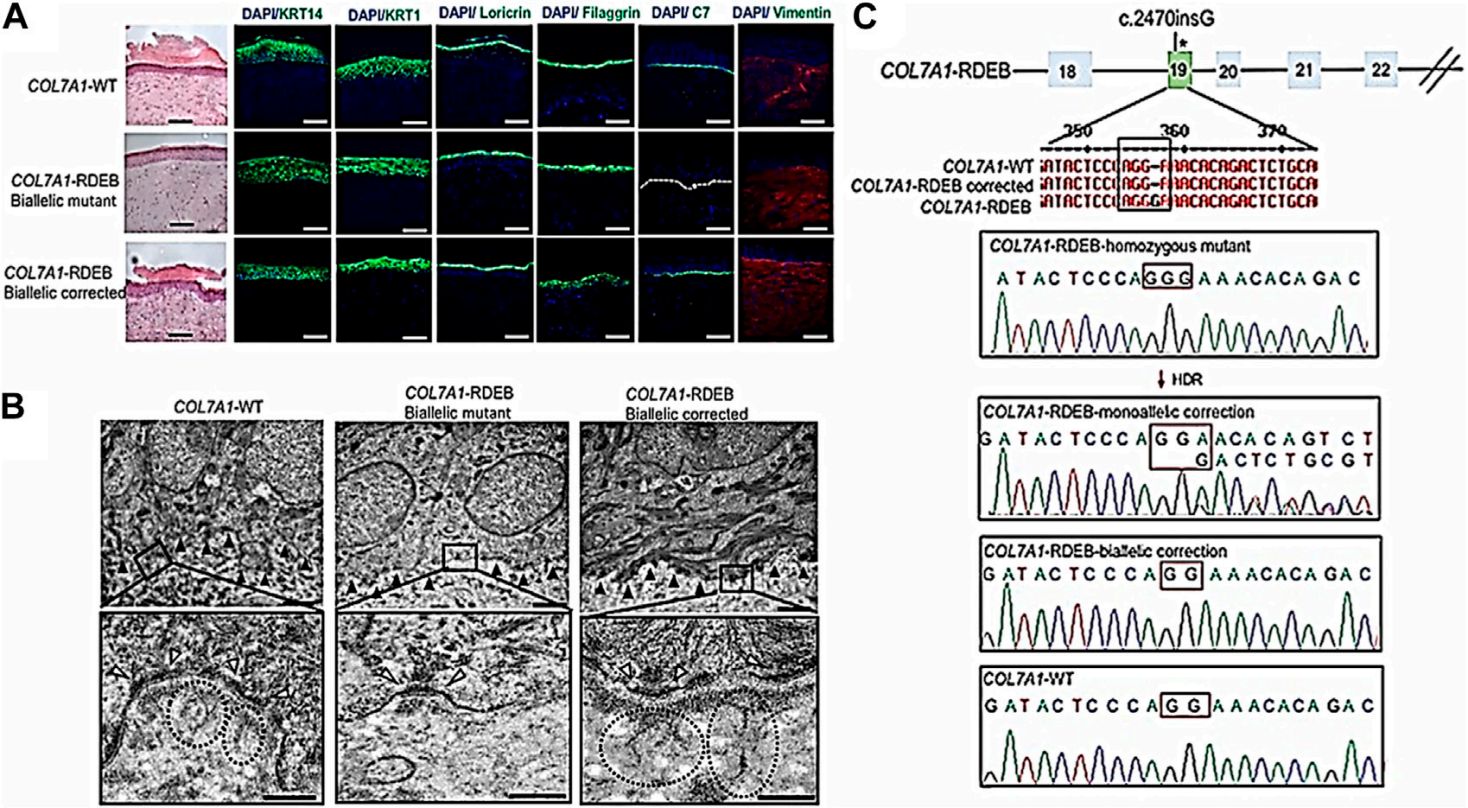
Functional validation of gene-corrected iPSC-derived FBs and HSEs from RDEB patients. **(A)** Histologically, 3D HSEs made from iPSC-derived KCs and FBs from gene-corrected RDEB patients are equivalent to those made using iPSC WT KCs/FBs, 2 months after grafting. Epidermal and dermal morphology were both normal, as shown by H&E staining. Immunofluorescence (IF) labeling (green signal), using the LH7.2 antibody, is used to show C7 deposition 2 months after grafting. On correction, mutant, and WT xenografts, further IF staining for keratin 14 and keratin 10 as well as loricrin, filaggrin, and vimentin was carried out. **(B)** Two months after grafting, transmission electron microscopy was used to examine positive iPSC WT KCs/FBs skin grafts, negative homozygous mutant COL7A1-RDEB skin grafts, COL7A1-RDEB gene-corrected KCs/FBs skin grafts, and gene-corrected RDEB skin grafts. **(C)** Sanger sequencing verified multiple genotypes in COL7A1-RDEB homozygous mutant iPSCs from designated colonies. (Adapted from Jacków et al., 2019).

In an investigation, researchers have developed a collagen-based hydrogel that may deliver CRISPR/Cas9 to the brain to treat neurological problems. Another study increased the efficiency of gene editing and improved the targeting of CRISPR/Cas9 to specific liver cells while reducing off-target effects by using a growth factor delivery technique. To treat skin diseases, CRISPR/Cas9 has also been administered via collagen-based delivery systems (Cota-Coronado et al., 2019; Zhou et al., 2022). In a scientific report, epidermolysis bullosa, a hereditary skin condition, was successfully treated using a hydrogel made of collagen that was utilized to transport CRISPR/Cas9 to skin cells. Xenografts made from biallelic corrected induced pluripotent stem cell (iPSC)-derived recessive dystrophic epidermolysis bullosa (RDEB), keratinocytes (KCs) and fibroblasts (FBs) were immunofluorescently labeled for C7, epidermal development markers such keratin 14, keratin 10, loricrin, filaggrin, and vimentin, and they were compared to iPSC-derived xenografts made from WT and mutant cells 2 months after grafting (Figures 4A, B). The fact that the xenografts from iPSCs that had their genes corrected generated C7 and looked exactly like WT skin suggests that the iPSC-derived iKCs and iFBs had their protein functionality recovered. The COL7A1 mutation in exon 19 was corrected in 10% of the selected clones by biallelic correction and in 40% of the clones through monoallelic correction. About 80% of the selected clones showed Cas9 activity. The hereditary mutation (c.2470insG) in exon 19 of COL7A1 was corrected by 58% biallelic and 42% monoallelic correction, whereas the heterozygous mutation (c.2470insG/c.3948insT) in exons 19 and 32 of COL7A1 was corrected by 19% biallelic and 48% monoallelic correction, respectively (Figure 4C). (Jacków et al., 2019). The effectiveness and security of CRISPR/Cas9 gene editing might be enhanced by collagen-based delivery methods, and current research in this area is expected to result in future developments (Prodinger et al., 2019; du Rand et al., 2022; Wu et al., 2017). Collagen-based therapeutic approaches must nevertheless be improved to distribute CRISPR/Cas9 precisely and effectively to target cells. In conclusion, collagen-based CRISPR/Cas9 delivery systems represent a fascinating and promising area of research with potential applications in the treatment of several inherited disorders (Kang et al., 2022).

#### 3.4.4 Gelatin

Gelatin is a protein made from collagen that has been employed in tissue engineering and medication delivery. Proteins, peptides, and medicines have all been delivered using gelatin-based delivery methods. They have also been employed in the creation of scaffolds for tissue engineering, such as those used to regenerate cartilage and skin. (Thein-Han et al., 2009; Afewerki et al., 2018). Gelatin may be used to administer a wide range of drugs, including proteins, peptides, and small compounds. The efficient synthesis of gelatin-based solid lipid nanoparticles for the delivery of the widely used chemotherapy drug paclitaxel (Ma et al., 2020; Wong et al., 2020).

**FIGURE 5:**
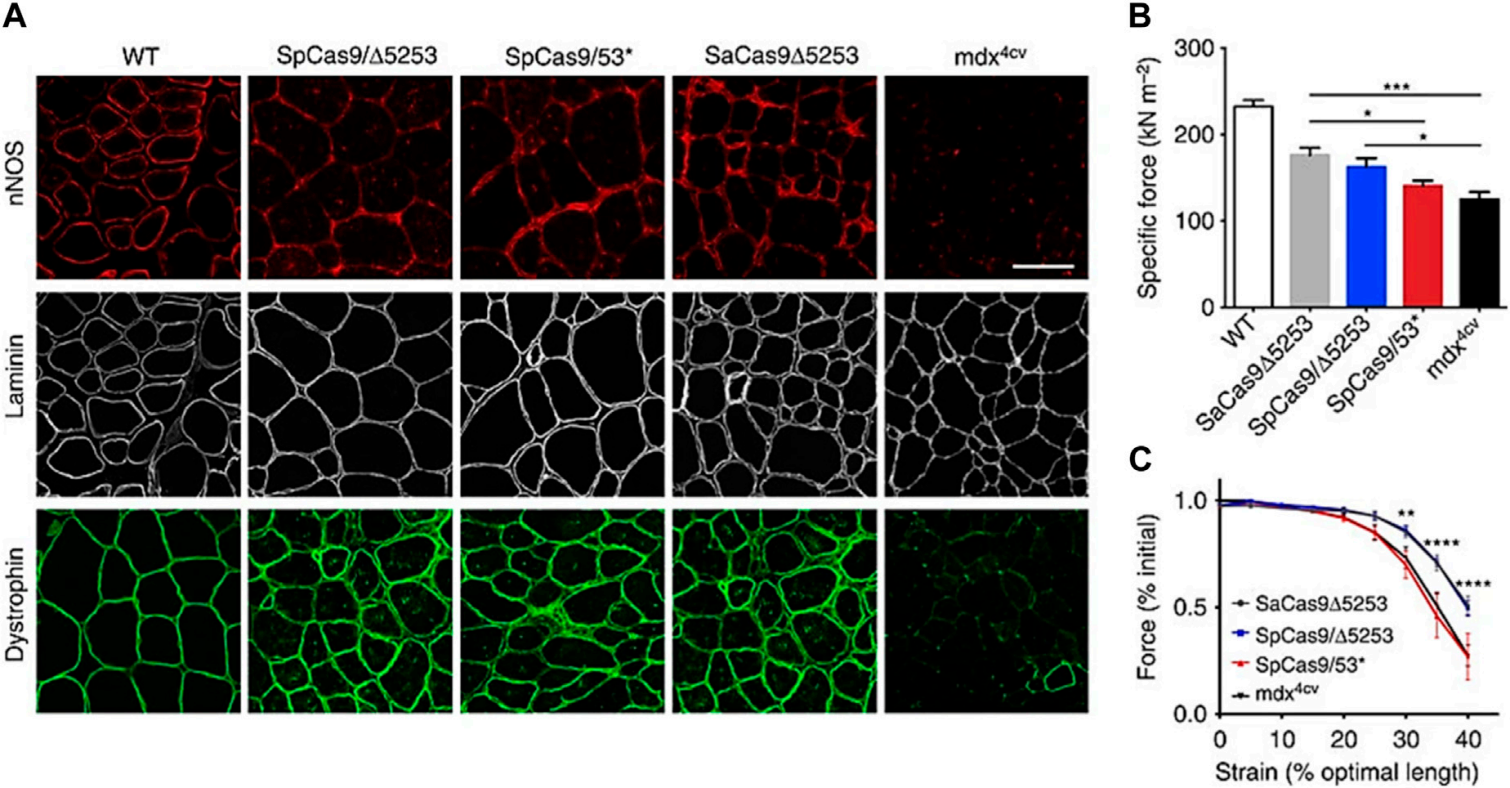
The localization of nNOS to the sarcolemma by dystrophin correction by CRISPR/Cas9 enhances muscular performance. **(A)** IM-treated and control muscles were immunofluorescent stained for nNOS, laminin, and dystrophin. **(B)** Specific force producing capacities of treated mdx4cv mouse TA muscles following IM transduction with each vector SaCas9/5,253, SpCas9/5,253, and SpCas9/53 as well as of untreated age-matched WT and mdx4cv muscles. **(C)** Resistance to damage caused by eccentric contraction, determined by evaluating contractile function in the moments before lengthening alterations during maximal force generation in the TA muscles of naive *versus* IM-treated mdx4cv mice SaCas95253, SpCas9/5,253, and SpCas9/53. (Adapted from Bengtsson et al., 2017, under the terms of the Creative Commons CC BY license).

To cure Duchenne muscular dystrophy in a mouse model, CRISPR/Cas9 has been delivered via gelatin nanoparticles, which has improved muscle function and raised dystrophin expression. The key enzyme neuronal nitric oxide synthase (nNOS), which is a part of the dystrophin-glycoprotein complex that regulates muscle function, was able to localize to the sarcolemmal region as a result of the induction of dystrophin expression (Figure 5). The detected dystrophin levels within muscles remained sustained, leading to notable gains in specific force-producing ability as well as protection from contraction-induced damage (Bengtsson et al., 2017).

Gelatin hydrogels were used as a delivery mechanism to target the carcinogenic KRAS gene by CRISPR/Cas9 in pancreatic cancer cells *in vitro*, which led to the reduction of cell proliferation and colony formation (Kim et al., 2018). Improved airway epithelial function and a decrease in bacterial load were the results of delivering CRISPR/Cas9 via gelatin-based nano complexes in a pig model of cystic fibrosis. To deliver CRISPR/Cas9 to glioblastoma cells *in vitro*, gelatin nanoparticles functionalized with tumor-targeting peptides have been created. This reduces cell survival and promotes tumor development (Zhou et al., 2019; Piotrowski-Daspit et al., 2022).

Gelatin-based delivery systems for CRISPR/Cas gene editing have become a viable tool for therapeutic uses because of their biomedical applications, good biocompatibility, and capacity to incorporate and preserve genetic information (Xu et al., 2020b). Gelatin nanoparticles equipped with Cas9 protein and guide RNA were shown to effectively transfer the elements to human embryonic kidney cells (HEK293T) and trigger gene editing, demonstrating the higher effectiveness with which gelatin nanoparticles can deliver CRISPR/Cas components to target cells (Rouet et al., 2018; Andrée et al., 2022).

It has been demonstrated that gelatin hydrogels can boost the long-term stability of the Cas9 protein and guide RNA by protecting it from nuclease degradation. Substantial levels of synthetic biology were also maintained by the gelatin hydrogels in HEK293T cells, demonstrating that gelatin hydrogels may improve the toughness and stop CRISPR/Cas components from deteriorating (Kim and Liu, 2019; Boucard et al., 2022; Li et al., 2022b). Another study created gelatin-based microcapsules that could deliver the Cas9 protein and RNA guide to certain mice organs, including the hepatic and pulmonary. Given how effectively the microparticles edited the genes in these tissues, gelatin-based microparticles might also successfully transport CRISPR/Cas components to specific tissues *in vivo* (Wang et al., 2016; Sahu et al., 2019). According to different research, gelatin-based nanoparticles have been created that can be tailored to deliver CRISPR/Cas to certain cells. Gelatin-based nanoparticles may be designed to target certain cells for CRISPR/Cas delivery, according to the investigators’ *in vitro* and *in vivo* experiments showing the nanoparticles could effectively carry Cas9 protein and guide RNA to cancer cells (Naeem et al., 2021; Xu et al., 2021; Saw et al., 2022).

#### 3.4.5 Silk protein

Because of its physiological differences, high functional properties, and capacity to create hydrogels, silk protein, generated from the silkworm, has been explored as a way of delivering pharmaceuticals (Huang et al., 2018). Silk-based distribution techniques have been developed for a variety of medications, including proteins, peptides, and small compounds. Silk-based hydrogels are being developed for the prolonged release of bone morphogenetic protein-2, a growth factor that stimulates bone repair (Yucel et al., 2014; Nguyen et al., 2019).

It has been demonstrated that silk fibroin nanoparticles effectively transport CRISPR/Cas9 to human glioblastoma cells and human embryonic kidney cells (HEK293T) (U87). The research established both biocompatibility and lack of toxicity of the silk fibroin nanoparticles in the cells. Gene editing was also demonstrated to be successful when CRISPR/Cas9 was supplied using silk fibroin nanoparticles (Zhao et al., 2015). In a different scenario, silk nanoparticles were used to administer CRISPR/Cas9 for genome - editing in the retina. Silk nanoparticles, according to the study’s findings, may effectively transport CRISPR/Cas9 to retinal pigment epithelium (RPE) cells both *in vitro* and *in vivo*. The research demonstrated that the silk nanoparticles were biocompatible and had no adverse effects on the cells (Salman et al., 2022). A hydrogel based on silk fibroin was created recently for the delivery of CRISPR/Cas9 for cartilage gene editing. The scientists discovered that the silk fibroin-based hydrogel could effectively transport CRISPR/Cas9 to the chondrocyte cells both *in vitro* and *in vivo*. The research revealed that the hydrogel made of silk fibroin was biocompatible and had no harmful effects on the cells (Yu et al., 2023a).

A study showed that silk fibroin nanoparticles may successfully edit genes *in vitro* and *in vivo* by effectively delivering CRISPR/Cas9 complexes into cells. In a different study, silk-based nano complexes were developed for the delivery of the CRISPR/Cas9 ribonucleoprotein (RNP) into cells. The authors established *in vitro* and *in vivo* effective gene editing with little off-target side effects (Wei et al., 2014). In the current research, a peptide was used to functionalize silk fibroin nanoparticles to improve their uptake by cells and endosomal escape. The authors successfully delivered CRISPR/Cas9 RNP into cells and successfully carried out both *in vitro* and *in vivo* genome engineering experiments (Esser et al., 2021; Pirota et al., 2023). The most recent developments in silk-based gene editing delivery technologies, including CRISPR/Cas9, were detailed in a review paper. The biodegradability, adjustable mechanical characteristics, and biocompatibility of silk fibroin were emphasized by scientists as advantages for gene transfer (Kochhar et al., 2021).

#### 3.4.6 Hyaluronic acid

Targeted medication delivery to cancer cells that overexpress hyaluronan receptors has been accomplished using hyaluronic acid-based delivery systems. It has been demonstrated that hyaluronic acid nanoparticles improve the effectiveness of anticancer medications like paclitaxel and doxorubicin (Maruyama, 2011; Lee et al., 2020). Sustained-release medication delivery systems have been created using cellulose derivatives such as methylcellulose and hydroxypropyl methylcellulose. With better drug release profiles and fewer side effects, these devices have been employed to administer medications including diclofenac and metformin (Hoare and Kohane, 2008; Wadher et al., 2011; Roy et al., 2013).

The CRISPR/Cas delivery mechanism hyaluronic acid (HA) has lately gained popularity. HA, a natively abundant glycoprotein found in the extracellular matrix of many tissues, is safe and well-tolerated in humans (Kelkar et al., 2016). In addition, HA is a top choice for the targeted delivery of CRISPR/Cas to cancerous cells because of its high affinity for CD44, a cell surface receptor that is upregulated in many cancerous cells (Wang and Jia, 2016; Xu and Li, 2020). The efficacy of HA-based delivery methods for CRISPR/Cas has been shown in numerous investigations. One research, for instance, delivered CRISPR/Cas9 to ovarian cancer cells both *in vitro* and *in vivo* using HA nanoparticles, which significantly inhibited tumor development (Vaghari-Tabari et al., 2022). Another study successfully achieved effective gene editing and the activation of death by delivering CRISPR/Cas9 to human lung cancer cells *in vitro* using HA-coated polymeric nanoparticles (Fang et al., 2022).

As a potential CRISPR/Cas delivery method, HA-based hydrogels have also been examined in other investigations. One experiment, for instance, used a hydrogel based on HA to deliver CRISPR/Cas9 to mice’s retinas while preventing retinal degeneration (Yang et al., 2021). A system of hyaluronic acid-based nanoparticles was used in one study to deliver CRISPR/Cas9 to cancer cells. The researchers showed that the CRISPR/Cas9 system could be effectively delivered to cancer cells *in vitro* using hyaluronic acid-based nanoparticles, resulting in precise gene editing and the induction of death (Liang et al., 2015; Qiu et al., 2016; Lin et al., 2022).

**FIGURE 6:**
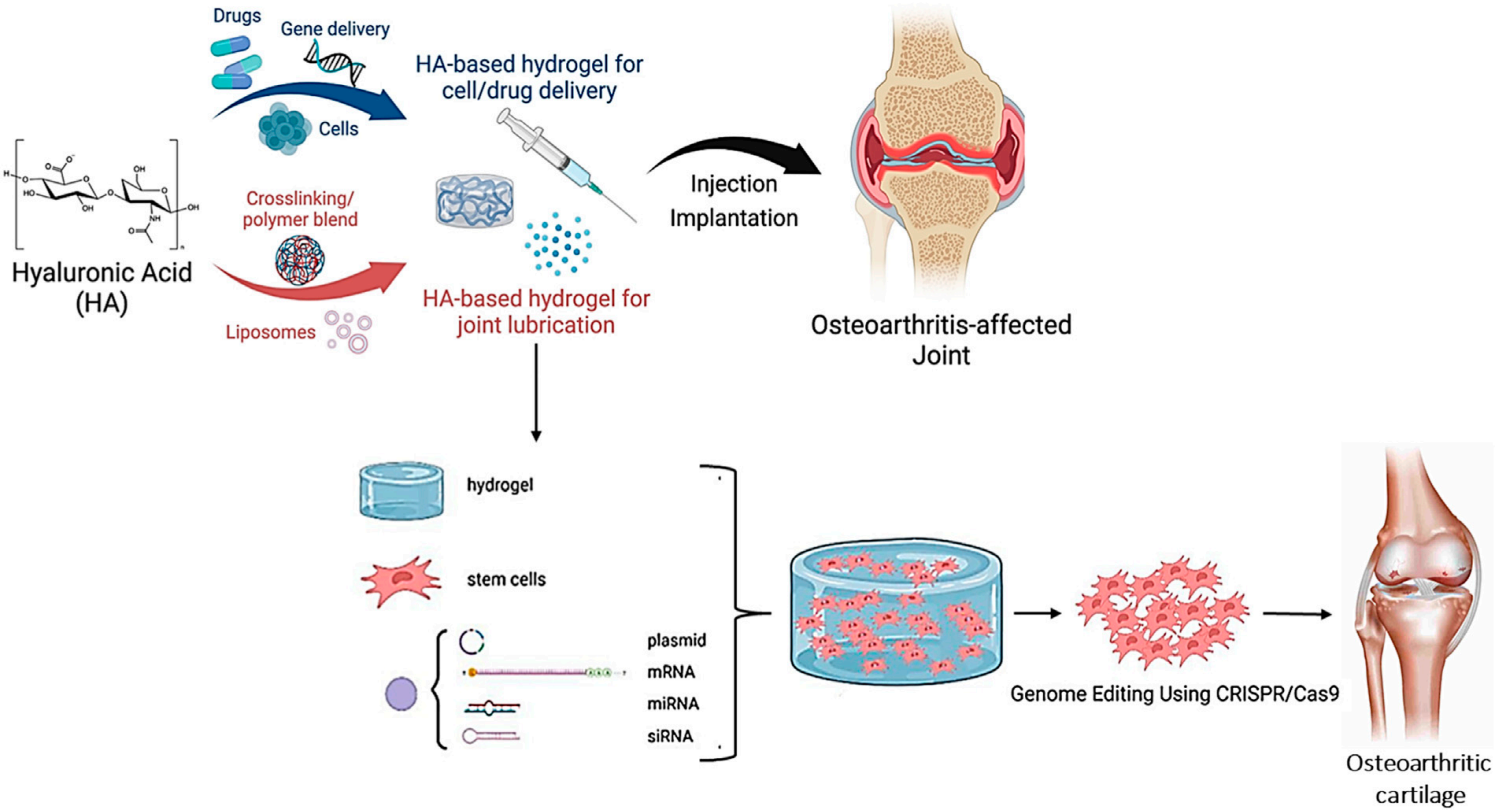
Diagrammatic description of hyaluronic acid-mediated CRISPR/Cas9 for osteoarthritis-affected joint and cartilage healing (Adapted from Liu et al., 2021a under the open access Creative Commons Attribution (CC BY) license).

Another study administered CRISPR/Cas9 to treat osteoarthritis using a hyaluronic acid hydrogel. The research showed that the CRISPR/Cas9 system could be successfully introduced into mesenchymal stem cells *in vitro* via the hydrogel, resulting in gene editing and cartilage regeneration (Figure 6) (Mehta et al., 2021; Kim and Guilak, 2022). A recent study demonstrated how to deliver CRISPR/Cas13a to the SARS-CoV-2 RNA using hyaluronic acid-based nanoparticles. The researchers showed that the CRISPR/Cas13a system could be successfully introduced into SARS-CoV-2-infected cells *in vitro* through hyaluronic acid-based nanoparticles, resulting in precise RNA cleavage and inhibition of viral growth (Chen et al., 2022; Deol et al., 2022).

#### 3.4.7 Lactose-derived branched cationic biopolymer

Branched cationic biopolymers generated from lactose are one class of biopolymers that have demonstrated potential as CRISPR/Cas9 delivery vehicles. These biopolymers are created from the disaccharide lactose, which is found in large quantities in milk (Qi et al., 2020). The positive charge and cationic nature of the lactose-derived biopolymers enable them to associate with negatively charged nucleic acids like DNA and RNA. These biopolymers’ branching structure offers the CRISPR/Cas9 complex several locations for attachment, enhancing delivery effectiveness. The lactose-derived biopolymers are also safe for *in vivo* applications since they are biocompatible and biodegradable (Moradali and Rehm, 2020; Xavier et al., 2021; Shtykalova et al., 2023).

Highly branched-charged biomaterials generated from lactose have recently been investigated as possible CRISPR/Cas9 delivery systems. Researchers demonstrated how lactose-derived branching cationic biopolymers may effectively carry CRISPR/Cas9 into cells, leading to considerable gene editing (Zhuo et al., 2021). Its effectiveness was ascribed by the researchers to the biopolymer’s capacity to create stable nanoparticles that can enter cell membranes (Sundar et al., 2010). Researchers examined the biocompatibility and toxicity of lactose-derived branched cationic biopolymers *in vitro* and *in vivo* in another investigation. The biopolymer was found to be non-toxic and did not cause any major inflammatory reaction, indicating that it might be a safe and effective CRISPR/Cas9 delivery vehicle (Rouatbi et al., 2022).

In a published paper, researchers showed how targeting receptors may be incorporated into lactose-derived branching cationic biopolymers to specifically transport CRISPR/Cas9 to cancer cells. The researchers’ *in vitro* and *in vivo* tests revealed extensive use of genetic engineering strategies and tumor growth inhibition (Kong et al., 2021; Chakraborty and Sarkar, 2022). In a thorough study that has been published, researchers asserted the potential of lactose-derived branched cationic biopolymers as CRISPR/Cas9 delivery vehicles for therapeutic purposes. The scientists talked about the biocompatibility, non-toxicity, targeting, and ability of the biopolymer to avoid issues with CRISPR/Cas9 distribution *in vivo* (Jie et al., 2021).

Several studies have concentrated on creating biopolymer-based CRISPR/Cas9 delivery systems as a potential replacement for viral vectors, which have drawbacks and safety issues. A lactose-derived branching cationic biopolymer known as lactose-grafted polyethyleneimine (Lac-PEI) is one intriguing possibility (Li et al., 2018; Iqbal et al., 2023). Target cells can effectively receive CRISPR/Cas9 plasmids using Lac-PEI. Researchers demonstrated in a study that Lac-PEI-mediated CRISPR/Cas9 delivery caused a considerable suppression of the target gene in human lung cancer A549 cells and human embryonic kidney (HEK293T) cells (Ryu et al., 2018).

According to a study, targeting peptide-coated Lac-PEI nanoparticles may effectively deliver CRISPR/Cas9 to breast cancer cells both *in vitro* and *in vivo*. Lac-PEI can be changed to increase delivery effectiveness and lessen toxicity (Nie et al., 2022). Researchers transformed Lac-PEI in a study that was published with the addition of a zwitterionic polymer, which increased the effectiveness of its gene transport and decreased both *in vitro* and *in vivo* cytotoxicity (Liu et al., 2022a). Multicarrier gene editing is now possible with Lac-PEI, wherein the researchers created a Lac-PEI-based CRISPR/Cas9 system in a study that allowed three genes to be edited in HEK293T cells simultaneously (Rohiwal et al., 2020). Furthermore, the development of more efficient and secure genome editing therapeutics shows promise with the use of lactose-derived branched cationic biopolymers for CRISPR/Cas9 delivery. To completely describe their features, enhance the effectiveness of their distribution, and improve their usage in therapeutic interventions, more study is required.

#### 3.4.8 Polyhydroxyalkanoates

Bacteria that experience inconsistent reproduction synthesize polyhydroxyalkanoates (PHA), a class of organic, biodegradable polyesters that operate as internal carbon- and energy-storing components. They have greater biodegradability, biocompatibility, and the capability to produce harmless byproducts, making them suitable replacements for implantable devices such as sutures, repair patches, slings, orthopedic pins, scaffold, stents, and adherence barriers (Lee, 1996; Guzik, 2021). Despite their great features as indicated above, unmodified PHAs are inappropriate for use in many biological applications due to the presence of large aggregates, which leads to poor biomechanical properties, poor thermodynamic stability, excessive hydrophobicity, and a sluggish disintegration rate (Ray and Kalia, 2017; Riaz et al., 2021). PHAs’ intrinsic hydrophobicity hinders them from being employed in biomedical applications as many biomedical devices require greater hydrophilicity (Pulingam et al., 2022).

PHAs are now being researched for their effectiveness as a CRISPR/Cas-based biomedical research framework. The CRISPR/Cas system is a potent genomic tool that enables precise editing of DNA sequences but getting it to cells correctly and effectively is still a big barrier (Jacinto et al., 2020). For instance, positively charged amino acids and negatively charged carboxyl groups can facilitate cell uptake of the CRISPR/Cas system. PHAs can encapsulate these functional groups and may also be modified to decay at predetermined rates, which is essential for managing the duration of CRISPR/Cas activity (Imamoğlu et al., 2022; Liu et al., 2022b).

It has been demonstrated that PHA-based nanoparticles may successfully distribute CRISPR/Cas systems *in vitro* and *in vivo*. One research, for instance, showed that Cas9 and sgRNA-loaded poly (3-hydroxybutyrate-co-3-hydroxy hexanoate) (PHBHHx) nanoparticles could effectively transport the CRISPR/Cas system to tumor cells *in vitro* and a mouse tumor model (Kılıçay et al., 2011; Chang et al., 2014). PHA scaffolds can also be used to distribute CRISPR/Cas systems locally. According to the study, gene modification was readily achieved in a bone-deficient mice model employing poly (3-hydroxybutyrate-co-3-hydroxy valerate) (PHBV) scaffolds loaded with Cas9 and sgRNA (Lv et al., 2015; Qin et al., 2018). Some traits can be included in PHAs to increase their potency as CRISPR-Cas delivery vehicles. For example, PHA nanoparticles may be functionalized with targeting ligands to improve their selectivity for specific cell types. A targeting peptide was added to PHA nanoparticles in a recently published study to enable the delivery of Cas9 protein and guide RNA to mouse liver cells in a targeted manner (Shrivastav et al., 2013; Prakash et al., 2022). PHAs may be used with other materials to create blended packaging solutions that have additional advantages. For instance, researchers have developed PHA-based hydrogels (embedding the Cas9 protein and guide RNA) that may be injected at a target site for permanent and precise genetic editing. Researchers developed a PHA-based hydrogel that, when implanted at the site of the tumor in mice, significantly inhibited the growth of tumors (Liu et al., 2021b).

PHAs have the potential to be used as a CRISPR/Cas delivery method, according to one published research, wherein to improve cellular absorption, the researchers created a PHA-based nanoparticle that was functionalized with amino groups. The CRISPR/Cas system was also included in the nanoparticle, and its capacity to trigger gene editing in human cells was examined. The outcomes demonstrated that the PHA-based nanoparticle efficiently and effectively introduced the CRISPR/Cas system into the cells and triggered gene editing (Allemailem et al., 2022; Xi et al., 2022). It is now possible to regulate the expression of endogenous or heterologous genes via the advent of transcriptional and/or configurable hereditary circuits, such as T7 polymerase-based expression vectors, programmable T7-based polymeric transposable elements, the RiboTite system, vector technology, and CRISPR-Cas tools. CRISPR/Cas9 has also been successfully used to change many genes simultaneously. CRISPR interference effectively directs metabolic flow toward PHA synthesis (Lv et al., 2015; Chen and Jiang, 2018).

In research, CRISPR/Cas9 ribonucleoprotein (RNP) complexes were shown to be transported into human cells using PHA nanoparticles. The study found that PHA nanoparticles effectively activate targeted gene editing and transport CRISPR/Cas9 RNP complexes in primary T cells and neurons (Cheng et al., 2022). Researchers also considered PHA-based nanomaterials for the *in vivo* delivery of CRISPR/Cas9 plasmids. The researchers successfully carried CRISPR/Cas9 plasmids to target cells and started gene editing in a mouse model using PHA nanoparticles. In addition to being studied as a delivery method for CRISPR-Cas systems, PHAs are also being investigated as a scaffold material for those systems (Zhang et al., 2022). In a different study, scientists demonstrated how to use a scaffold made of PHA and CRISPR/Cas9 plasmids to treat bone defects. The researchers found that the PHA-based scaffold could effectively distribute the CRISPR/Cas9 plasmids and promote bone regeneration in a rat model (Liu et al., 2023).

#### 3.4.9 Exosomes

Exosomes are extracellular vesicles that may be separated from a variety of physiological fluids, including blood, urine, and saliva. They are released by practically all cell types. They are rapidly being researched for their prospective use as drug carriers for pharmacological medicines, including CRISPR/Cas gene editing tools. They play a significant part in signaling pathways (Muthu et al., 2021; Rajput et al., 2022). Potential therapeutic uses for exosome-based CRISPR/Cas delivery include the treatment of cancer, genetic abnormalities, and viral infections. According to researchers, for instance, exosomes can carry siRNA to cancer cells and prevent the development of tumors in mice; Cas9 protein and sgRNA to cure Duchenne muscular dystrophy in a mouse model (Kooijmans, et al., 2013; Ahmadi et al., 2023).

If CRISPR/Cas is administered via exosomes, gene therapy might undergo a radical transformation. By separating exosomes from diverse sources, including cells generated by patients, CRISPR/Cas components may be produced and directed toward specific genes or mutations connected to certain diseases (Zhu et al., 2023). Studies have demonstrated promising results for the delivery of CRISPR/Cas for the treatment of several diseases, including cancer and genetic disorders. Exosome-mediated CRISPR/Cas delivery is superior to other delivery methods, including viral vectors and lipid-based transfection. This is owing to exosomes’ capacity to identify certain tissues and shield the payload from destruction by proteolytic enzymes (McAndrews et al., 2021; Wan et al., 2022). CRISPR/Cas administration through exosomes has been utilized successfully in experimental animals to cure hereditary mutations. In a mouse model of Duchenne muscular dystrophy, for example, exosomes containing CRISPR/Cas were capable of targeting muscle fibers and fixing the dystrophin mutated gene, resulting in enhanced neuromuscular activity (Erkut and Yokota, 2022; Zhang et al., 2023).

**FIGURE 7:**
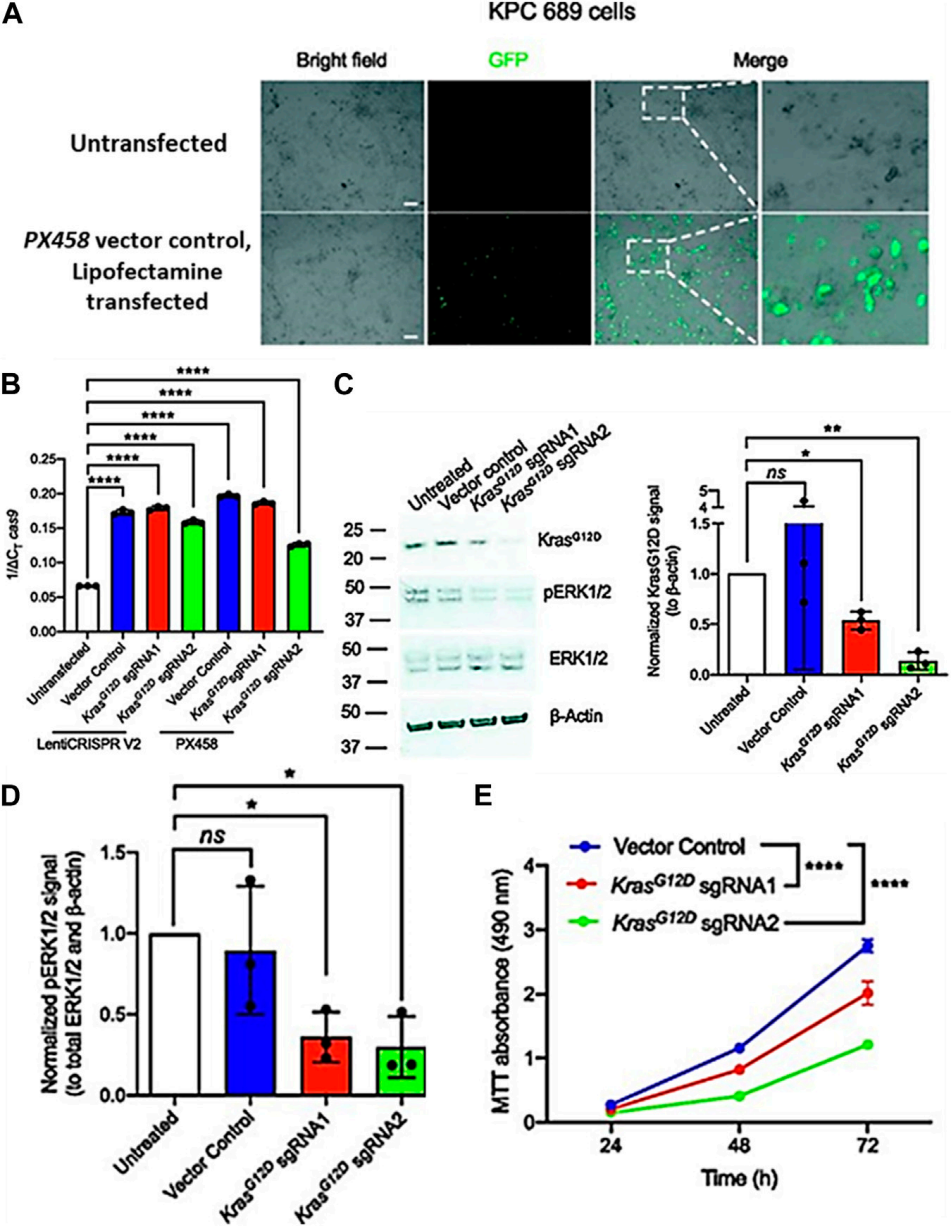
Exosome-mediated delivery of CRISPR/Cas9 disrupts oncogenic KrasG12D *in vitro* and inhibits proliferation. **(A)** Epifluorescence microscopy imaging was used to evaluate the transfection efficiency of lipofectamine 2000 by using GFP/Cas9 vector control (PX458) plasmid. **(B)** Quantitative PCR was used to evaluate mRNA expression levels of Cas9. **(C)** Western blot for KrasG12D, pERK1/2, total ERK1/2, and β-actin of KPC689 cells following treatment with exosomes containing CRISPR/Cas9 plasmid DNA. **(D)** Knockdown of mutant Kras signaling at the protein level was confirmed via Western blot for KrasG12D and its downstream effector pERK1/2. **(E)** KPC689 cells were treated with exosomes carrying CRISPR/Cas9 plasmid DNA for 72 h, and cell survival and proliferation rates were assessed using the MTT test. (Adapted from McAndrews et al., 2021).

Several studies have shown that exosomes may successfully transport CRISPR/Cas components, such as sgRNA and Cas9 protein, to target cells *in vitro* and *in vivo*. For example, researchers discovered that exosomes generated by dendritic cells may transport siRNA to T cells and reduce the expression of a target gene (Haney et al., 2015). Similarly, researchers also demonstrated that exosomes may transport Cas9 protein and sgRNA to target cells and produce targeted genome editing (Chen et al., 2019). By directing the distribution of the CRISPR/Cas components to particular cell types and preventing off-target effects, exosomes can increase the precision and safety of CRISPR/Cas gene editing. For instance, research has shown that mesenchymal stem cell-derived exosomes may carry siRNA to breast cancer cells specifically and decrease the expression of a target gene (Lamichhane et al., 2016). Similar to this, another research showed that exosomes may cause targeted genome editing by only delivering Cas9 protein and sgRNA to pancreatic beta cells while having no negative effects on the function of other cell types (Chen et al., 2021). When KPC689 cells were treated with exosomes containing the KrasG12D sgRNA1 and KrasG12D sgRNA2 but not with exosomes containing the vector control or untreated cells, the T7/Surveyor test revealed indications of effective gene editing (Figure 7) (McAndrews et al., 2021).

In the context of research on infectious diseases, exosome-based CRISPR/Cas9 delivery has been suggested as a potential strategy for producing innovative therapies for viral infections including HIV and hepatitis B. For instance, a 2018 study discovered that exosomes containing CRISPR/Cas9 may target and harm HIV proviral DNA *in vitro*, decreasing the virus’ ability to produce genetic material (Dubey et al., 2022; Mazurov et al., 2023). Like this, exosome-based CRISPR/Cas9 delivery was shown to effectively target the hepatitis B virus (HBV) genome *in vitro* and *in vivo*, leading to a reduction in viral replication and gene expression (Martinez et al., 2022). Moreover, tissue engineering applications using exosome-based CRISPR/Cas9 dissemination have been studied, notably in the context of regenerative medicine. For instance, exosomes containing CRISPR/Cas9 were able to accurately target and modify the genes governing osteogenic differentiation in human mesenchymal stem cells, improving bone creation *in vivo* (Guo and Huang, 2022) Like this, it was demonstrated that exosomal delivery of CRISPR/Cas9 enhanced the capacity of human umbilical vein endothelial cells to produce angiogenic factors, suggesting the possibility of their use in tissue engineering and wound healing (Liu et al., 2021c; Zhao et al., 2023).

#### 3.4.10 Liposomes

Lipid bilayers in the liquid phase comprise the globular entities known as liposomes. Since both nucleic acids and biological membranes have negative charges, nucleic acids cannot pass through the membrane owing to the attraction between both (Yip, 2020). In this regard, positively charged liposomes encase negatively charged nucleic acids, making it easier for the complexes to pass cell membranes and enter cells. DNA, mRNA (Cas9 and sgRNA), or protein can all be used to deliver the CRISPR/Cas9 system (RNP) (Pensado et al., 2014). The spherical liposomes, made of a lipid bilayer, may store both hydrophilic and hydrophobic medications. Polymeric nanoparticles, made of natural or synthetic polymers, can be engineered to release medications in response to various stimuli, such as pH or temperature changes. Viral shape and functionality can be mimicked by protein-based nanoparticles, such as virus-like particles (VLPs), which are made of proteins (Sun et al., 2014; Bulbake et al., 2017; Nsairat et al., 2022).

A mouse model of Duchenne muscular dystrophy (DMD) was employed in a study to deliver CRISPR Cas9 to the dystrophin gene using liposomes. The scientists demonstrated that the liposome-delivered CRISPR Cas9 could correct the dystrophin gene mutation and enhance mouse muscle function (McGreevy et al., 2015). According to a different study, CRISPR Cas9 was delivered using liposomes to target the β-catenin gene in colorectal cancer cells. The scientists proved that CRISPR Cas9 administered by liposomes might stop tumor development both *in vitro* and *in vivo* (Chen et al., 2017; Jubair et al., 2021).

A mouse model of hypercholesterolemia was employed in another work to deliver CRISPR Cas9 to the PCSK9 gene via liposomes. The scientists demonstrated that the liposome-delivered CRISPR Cas9 could drastically lower the mice’s blood cholesterol levels (Ding et al., 2014; Chadwick and Musunuru, 2017). In a mouse model of acute myeloid leukemia (AML), liposomes were employed to carry CRISPR Cas9 to target the CXCR4 gene in a recent study. The scientists demonstrated that CRISPR Cas9 supplied by liposomes might cause apoptosis in AML cells and dramatically increase the longevity of the mice (Hou et al., 2015; Freen-van Heeren, 2022; Li et al., 2022c).

**FIGURE 8:**
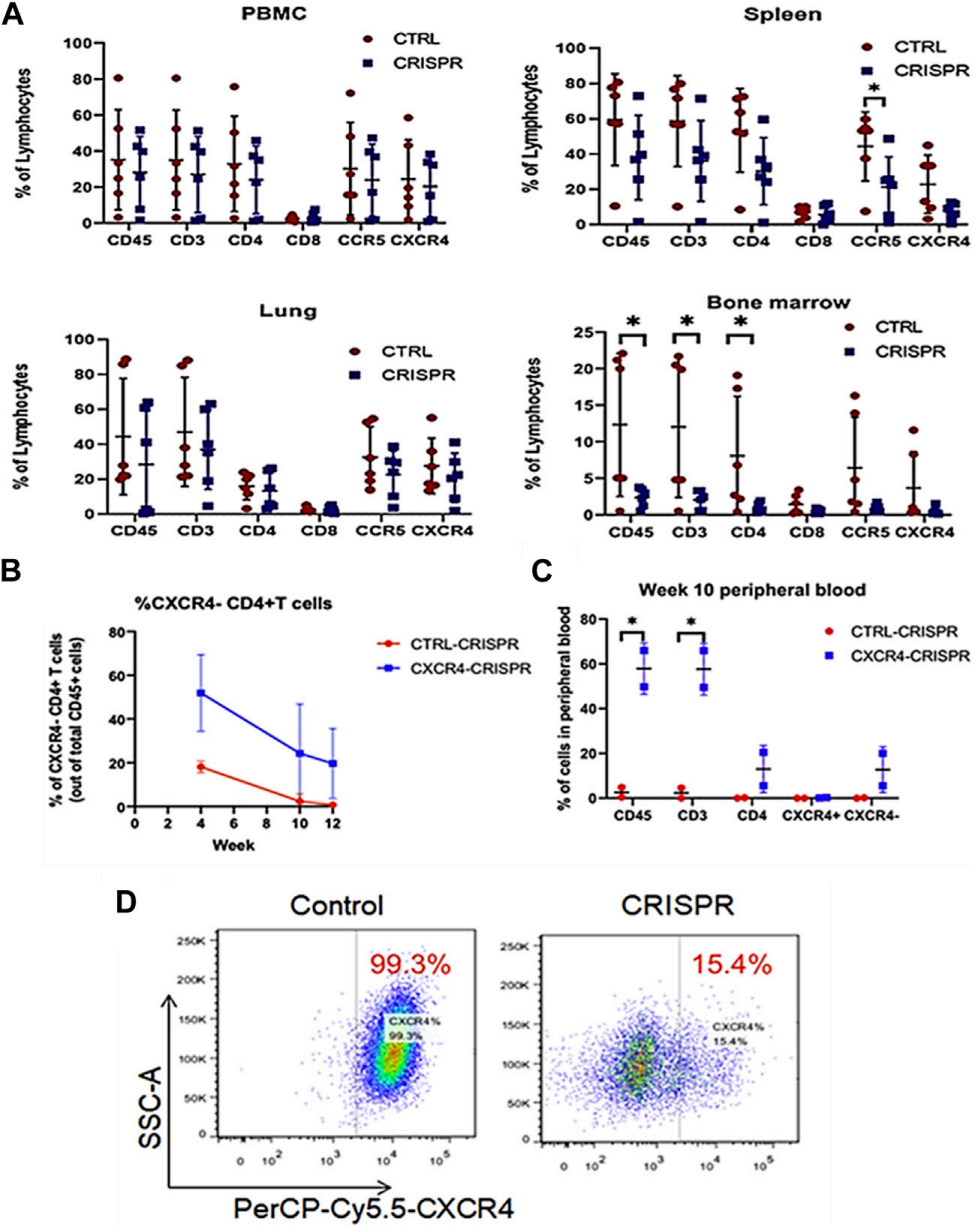
CCR5 and CXCR4-CRISPR deletion CD4^+^ T cell biodistribution in Hu-PBMC murine tissues **(A)** Human PBMCs combined with CRISPR-modified or unmodified CD4^+^ T cells were injected into NSG mice. All of the animals from each group’s entire PBMCs, spleens, lungs, and bone marrow were collected, and cells were examined using a flow cytometer. **(B**, **C)** Utilizing CXCR4 CRISPR-modified or unmodified cells (control), flow cytometry study of human CD3^+^ T lymphocytes in mice whole blood following transplantation. **(D)** Using flow cytometry, CXCR4 surface expression was examined in Jurkat cells that had either been transduced with the CXCR4 CRISPR or control vector. (Adapted from Li et al., 2022a).

Although animals retained their percentages of gene-modified cells across time (Figure 8A), the CRISPR-modified cells in the hu-PBMC NSG mouse model were well tolerated. Additionally, X4-tropic HIV-1 resistance in CXCR4-CRISPR humanized mice led to selective enrichment of CD4^+^ T cells in spleen tissue compared to non-CRISPR animals. Despite the fact that CRISPR-mediated disruption of CXCR4 was effective in lowering viremia and safeguarding CD4^+^ T cells *in vivo* (Figures 8B, C), researchers noticed the numbers of R5X4-CRISPR-modified CD4^+^ T cells were much lower than unmodified controls in the bone marrow. To halt Jurkat CD4^+^ T cells from expressing CXCR4 on their surface, researchers first tested the efficiency of various sgRNAs for each target through lentiviral vectors. Flow cytometry analysis revealed a significant reduction in surface CXCR4 expression, with 15.4% CXCR4+ cells transduced with CXCR4-CRISPR compared to 99.3% CXCR4+ cells in control-CRISPR cells (Figure 8D).

According to a scientific report, liposomes successfully delivered CRISPR/Cas9 to mice’s livers. The Cas9 mRNA and guide RNA (gRNA) targeting the PCSK9 gene were delivered by the investigators using a lipid nanoparticle (LNP) formulation. When contrasted with the control group, they discovered that the mice treated had much lower levels of PCSK9 protein and LDL cholesterol (Chadwick et al., 2017; Carreras et al., 2019). In another work, mRNA, and gRNA for Cas9 were delivered to primary human T cells using a dual-lipid nanoparticles method. In addition to effective gene editing, proliferation and differentiation, good cell vitality, and reduced toxicity were all noted by the researchers. Using the same delivery method, they also showed effective deletion of the target gene *in vivo* (Rosenblum et al., 2020; Han et al., 2022b).

Investigators designed a liposomal delivery mechanism in a study that targets the oncogenic Bcl-2 in leukemia cells using Cas9 and sgRNA. The technique was discovered to effectively transport Cas9/sgRNA and cause apoptosis in the leukemia cells, pointing to its potential as a leukemia treatment method (Li et al., 2020). A lipid nanoparticle-based delivery method for Cas9 mRNA and sgRNA targeting Angptl3 in mice was created by researchers in a different study. The technique was discovered to effectively carry the gene editing apparatus to the liver, leading to a reduction in Angptl3 expression and an improvement in the mice’s lipid metabolism (Qiu et al., 2021; Syaharani et al., 2021).

Another subject that has been studied is CRISPR/Cas9 delivery using cationic liposomes. In a recently published work, bio-reducible cationic lipids nanoparticles (CLN) were developed for the delivery of Cas9 and sgRNA (Li et al., 2019). Targeted mutagenesis knockout and correction were observed *in vitro* and *in vivo*, and this was due to the efficient transport of the gene editing machinery to cells via the bCLN. The administration of CRISPR/Cas9 using liposomes for cancer therapy has also been studied. (Zuris et al., 2015). The oncogene KRAS in pancreatic cancer cells was targeted using a liposomal delivery system developed by researchers for Cas9 and sgRNA. The system’s ability to deliver gene editing tools and cause cancer cells to die suggests that it may be useful as a pancreatic cancer therapy strategy (Won et al., 2021; Katti et al., 2022).

### 3.5 Nanobiotechnology-based delivery systems for CRISPR/Cas9 delivery in gene therapy and infectious diseases management

The CRISPR/Cas system has become a strong candidate for the development of future-oriented antimicrobial medications to treat infectious diseases, notably the ones brought on by antimicrobial resistance (AMR) microbes, as a result of its remarkable efficacy of RNA-guided nucleic acid destruction (Duan et al., 2021b; Getahun et al., 2022). Furthermore, the CRISPR/Cas system’s various adaptability permits it to accurately eradicate a particular bacteria isolate species from among an extensive community, enabling CRISPR/Cas bacteriocins to alter the structure of a diverse bacterial species (Shabbir et al., 2019). Due to this, CRISPR/Cas antimicrobial compounds are especially beneficial for treating diseases in complex endogenous microbiological associations, such as the gut microbiome (Gholizadeh et al., 2020).

Targeting microorganisms that are highly pathogenic and/or resistant to antibiotics is a therapeutic advantage of CRISPR technology (Palacios Araya et al., 2021). Because they can eliminate bacteria depending on their sequence, CRISPR-mediated antimicrobials have a clear advantage over existing antimicrobial techniques. This might be helpful in circumstances when only a few bacteria from a genus need to be removed, which is challenging to perform with present methods (Gholizadeh et al., 2020; Uribe et al., 2021). It clarifies the fundamental host-microbe connections, advances in quick and accurate diagnostic techniques, and enhances measures for the prevention and treatment of infectious illnesses (Caliendo et al., 2013).

The emergence of more harmful and aggressive bacterial strains is significantly aided by the transfer of virulence and drug resistance by foreign DNA. It has been demonstrated that CRISPR/Cas systems cause toxicity and antimicrobial resistance in plasmids and phages (Tao et al., 2022). For medical therapy to be most effective and for the appropriate creation of tailored treatments and vaccines, strategies to understand the microorganisms (bacteria, fungi, and viruses) that cause human infection are required. This is done by reporting the gene and protein that contributes to biological pathogens in CRISPR/Cas9-based gene editing using a variety of pathogens (Sydnor and Perl, 2011; Binnie et al., 2021). A tabular summarization of the diseases with their associated genes and clinical trials is described in Table 2.

**TABLE 2:** Diseases and their associated genes in the CRISPR/Cas 9 therapeutic approach.

Disease	Gene Target	CRISPR/Cas9 Approach	Outcome	Clinical Stage/Trial	Reference
β-Thalassemia	HBB	*Ex vivo* editing of patient’s cells	Increased hemoglobin production	CTX001 by CRISPR Therapeutics and Vertex Pharmaceuticals (Phase 1/2)	Frangoul et al. (2021); NCT03655678
Sickle Cell Anemia	HBB	*Ex vivo* editing of patient’s cells	Increased hemoglobin production	CTX001 by CRISPR Therapeutics and Vertex Pharmaceuticals (Phase 1/2)	Frangoul et al. (2021); NCT03745287
Leber Congenital Amaurosis	CEP290	*In vivo* delivery to retinal cells	Improved vision	EDIT-101 by Editas Medicine and Allergan (Phase 1/2)	Leroy et al. (2021); NCT03872479
Usher Syndrome	USH2A	*In vivo* delivery to retinal cells	Improved vision	EDIT-101 by Editas Medicine and Allergan (Phase 1/2)	Whatley et al. (2020); NCT03780257
Hereditary Transthyretin Amyloidosis	TTR	*In vivo* delivery to liver cells	Reduced amyloid deposition	NTLA-2001 by Intellia Therapeutics (Phase 1)	Gillmore et al. (2021); NCT04601051
Duchenne Muscular Dystrophy	DMD	*In vivo*/*ex vivo* delivery to muscle cells	Restored dystrophin expression	SRP-9001 by Sarepta Therapeutics (Phase 1/2)	Mendell et al. (2023); NCT05096221
Cystic Fibrosis	CFTR	*In vivo* delivery to lung cells	Restored CFTR function	VX-814 by Vertex Pharmaceuticals (Phase 2)	Dumas et al. (2021); NCT04167345
HIV Infection	CCR5	*Ex vivo* editing of patient’s cells	Reduced HIV viral load	adenovirus-associated virus vector serotype 9 (AAV9) (EBT-101) by Excision BioTherapeutics	Freen-van Heeren (2022); NCT05144386; NCT05143307
Autosomal Dominant Hyper-cholesterolemia	PCSK9	*In vivo* delivery to liver cells	Reduced LDL cholesterol levels	NTLA-2002 by Intellia Therapeutics (Preclinical)	Sufian and Ilies. (2023)
Duchenne Muscular Dystrophy	DMD, PF	Intravenous Infusion	Dystrophin expression & distribution	PF-06939926 gene therapy by Pfizer (Phase 1/Phase 3)	Deng et al. (2022) NCT03362502; NCT04281485

Gene therapy medications differ in drug transport and gene knockdown efficacy in distinct cell lines targeting different genes depending on their physical characteristics and mode of action (Pan et al., 2021). As a result, it's important to pick gene therapy medications that are appropriate for particular cell lines and target genes. Compared to other gene medications, the CRISPR/Cas9 technology can achieve gene deletion without any mRNA background by removing the target gene’s DNA molecules, whereas other gene drugs can only target mRNA molecules and intervene at the RNA level with unknown consequences (El-Kenawy et al., 2019; Abdelnour et al., 2021).

The ability to alter the genome is becoming more and more accessible thanks to recent developments in CRISPR, TALEN, and ZFN technologies, which are being widely used in biomedical studies, pharmaceutical development, and therapeutic gene therapy (Yin et al., 2017). Considering that less than 5% of rare medical conditions have access to efficient therapies, this is significant in terms of personalized medicine. More than 3,000 human genes have been linked to Mendelian disorders. Many of these uncommon disorders can now be cured because of developments in genome manipulation (Goetz and Schork, 2018; Šimić, 2019). But for genome-editing technologies to successfully target and infiltrate targeted organs and cells, whilst simultaneously avoiding toxicities, safe and reliable administration is still required (Mitchell et al., 2021).

The promise of CRISPR/Cas9 delivery methods based on nanobiotechnology in diverse gene therapy applications has recently been shown in research. In a mouse model of hereditary tyrosinemia type 1, researchers demonstrated the effective transport of CRISPR/Cas9 to the liver using lipid-based nanoparticles. The scientists demonstrated how effectively the lipid nanoparticles carried CRISPR/Cas9 to the liver, leading to a substantial decrease in the concentrations of deleterious byproducts (Alves-Bezerra et al., 2019; Huang et al., 2022b; Xu et al., 2022). Corresponding to this, researchers also reported the application of PLGA nanoparticles to deliver CRISPR/Cas9 to cancer cells with precision *in vitro*. The researchers demonstrated how CRISPR/Cas9 was successfully administered to cancer cells using PLGA nanoparticles, which also significantly increased the amount of cellular apoptosis (Rezvantalab et al., 2018; Nie et al., 2022).

Pluripotent stem cells (IPSCs) generated from patients with Duchenne muscular dystrophy (DMD) have been altered and corrected to correct various DMD mutations. Researchers developed the CRISPR/Cas9 method, which is employed to fix 60% of the severe readings in the coding abnormalities of DMD individuals, to repair the ∼700 kb mutant segment using non-homologous end joining (NHEJ) (Danisovic et al., 2018; Min et al., 2019). This approach is the most recent DMD deletion strategy based on the biggest segment mended by NHEJ using CRISPR/Cas9. Cardiomyocytes and skeletal muscles produced from IPSC cell clones using the aforementioned gene editing technique exhibit dystrophin transcription and functioning, thereby enhancing the integrity of the membrane and the complexity of glycoproteins, according to *in vivo* investigations in mice (Young et al., 2016; Pan et al., 2021).

Exosome RNPs, a brand-new approach to gene editing delivery, were developed by Wan et al. In mouse models, they cured hepatocellular cancer, chronic liver fibrosis, and severe liver injury. They electroporated Cas9 and sgRNA targeting KAT5, p53, and cyclin E1, and enhanced the regulation of apoptosis into exosomes produced by the LX-2 cell line. They discovered that RNPs exhibited an increased medicinal impact when delivered in EVs as opposed to RNPs alone and that the exosomes’ capacity to target liver cells improved tissue selectivity. This study demonstrated that exosomes from LX-2 cell lines might be used in liver tumor therapies (Ding et al., 2021; Wan et al., 2022; Sahel et al., 2023).

As young rodents synthesize mutant peptides with an intrinsic expression comparable to PARK17, Ishizu and colleagues, employed Vps35 D620N knock-in mice to study the physiological relevance of PARK17 *in vivo*. The mouse PARK17 gene’s exon 15 is removed using the CRISPR/Cas9 method. Significantly lowering dopamine production from the substantia nigra in the midbrain, has a specific impact on lessening Parkinson’s Disease symptoms (Ishizu et al., 2016). A novel CRISPR/Cas9 gene editing system was developed by Merienne and colleagues. It contains gRNA addressing the HTT gene (sgHTT) and gRNA targeting the Cas9 gene (sgCas9), allowing the technique to delete the Cas9 gene via sgCas9 whilst deleting the HTT gene. This method can decrease the expression of mHTT while considerably reducing the off-target impact (Merienne et al., 2017).

By modifying LNPs and incorporating phenylboronic acid (PBA) into the cationic lipid PBA-BADP, researchers reported creating a cell-selective CRISPR/Cas9 genome-altering delivery mechanism that focuses on malignant tumor cells that excessively express sialic acid (SA) via the interface PBA/SA association (Tang et al., 2019). Such LNPs can target malignant cells with high specificity while delivering the tumor suppressor p53 mRNA to stop the growth of those cells dramatically. PBA-BADP/Cas9 mRNA LNPs were delivered specifically and were more effective in suppressing the expression of genes in HeLa cancer cells than in non-malignant cells. These results demonstrated the development of an innovative lipid nanocarrier for tumor-targeted gene therapy (Rosenblum et al., 2020; Zhang et al., 2021b).

To administer CRISPR/Cas9-mediated gene therapies to treat non-small-cell lung cancer (NSCLC), Wang and colleagues created a multifunctional nonviral vector. Investigators added protamine sulfate to the Cas9/sgMTH1 plasmid to give it a negative charge before coating it with cationic liposomes (Wang et al., 2022). To improve longevity in circulatory and tumor selectivity, DSPE-PEG-hyaluronic acid (HA) was added to the liposomes. To enable regional administration of CRISPR/Cas9-based gene therapy, LNP delivery mechanisms can be altered. Lung cancer can be effectively treated with a dry powder formulation of LNP-embedded microparticles equipped with the CRISPR/Cas9 gene therapy tool when administered locally (Balon et al., 2022; Carneiro et al., 2023).

To modify the activity of glucagon-like peptide 1 for the treatment of type 2 diabetes mellitus (T2DM), researchers produced lecithin nano liposomal particles complexing sgRNA sequences, particularly for dipeptidyl peptidase-4 gene/Cas9 protein. SNU398 human liver cancer cells were used for *in vitro* research on the complex, while T2DM db/db mice were used for *in vivo* research (Cho et al., 2019). It was discovered that *in vitro* genome editing has a 31% and 39% *in vivo* effectiveness with little off-target consequences. By utilizing lipid nanoparticles as carriers, researchers showed that >97% of the target protein (transthyretin) was knocked down and they increased the longevity of genome editing to at least 12 months. The scientists showed that in contrast to viral vectors, multi-dosing using lipid nanoparticles is feasible to generate the best gene editing results (Finn et al., 2018; Gillmore et al., 2021; Taha et al., 2022).

## 4 Challenges and future prospects

**FIGURE 9:**
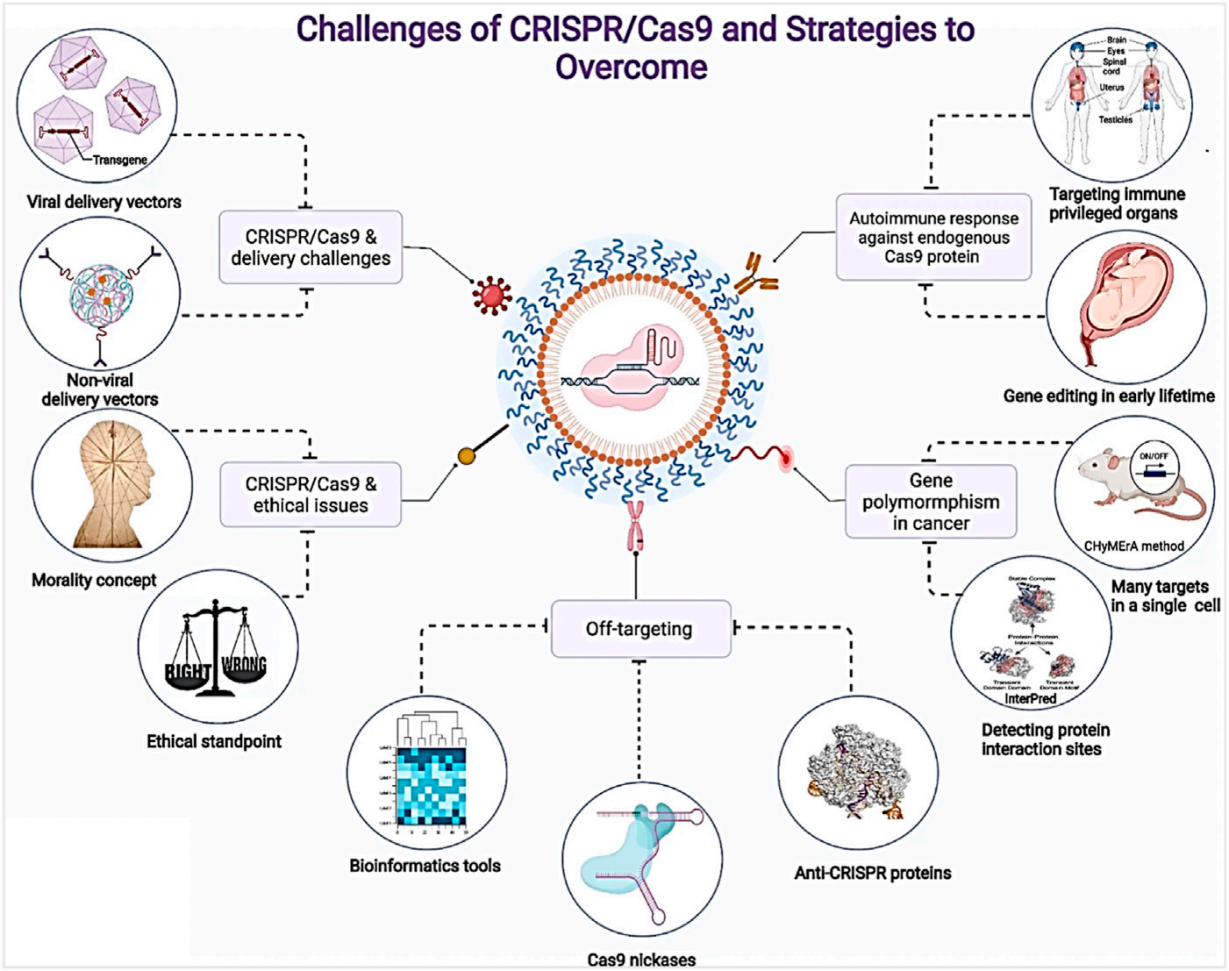
Challenges associated with CRISPR/Cas9 delivery and strategies to overcome them. (Adapted from Rasul et al., 2022, under a Creative Commons Attribution 4.0 International License).

The convergence of nanotechnology, biomaterials, and personalized medicine offers an opportunity to progress within both fields. Although nanoparticles are presently being assessed in unspecified patient groups, the implementation of biomaterials tailored to particular populations of patients could provide opportunities for the rapid translational application of a variety of nanomaterials (Salata, 2004; De Matteis et al., 2018). The effectiveness of personalized medicine, on the other hand, is dependent on firmly categorized clinical populations, and the utilization of nanoparticles to enhance the absorption throughout diverse physiological barriers may enhance the beneficial effects of precision medications, permitting additional individuals to be encompassed in the stratified population as well as expanding the possibility of a positive transformation to the center (Seyhan and Carini, 2019; Mitchell et al., 2021; Alghamdi et al., 2022; Joseph et al., 2023). Even though this technology has shown significant promise in diagnoses and other site-specific functions, several problems such as its immunogenicity, off-target site delivery techniques, and cytotoxicity must be resolved before it can be employed for *in vivo* gene therapy on humans (Figure 9) (Rasul et al., 2022). While this strategy has several advantages and prospects, it has certain challenges that need to be addressed for successful implementation.

### 4.1 Challenges

1. Off-Target Effects: Cas9 has been shown in several studies to attach to unexpected genomic locations for cleavage, a process known as off-target effects. Off-target CRISPR/Cas-mediated genome treatment would severely limit its utility since it might induce genetic instability and raise the risk of certain illnesses by adding undesired mutations in non-target regions. CRISPR/Cas9 gene editing has the potential to cause off-target effects, which are unexpected changes in the genome (Zhang et al., 2015; Dubey et al., 2022; Guo et al., 2023). By enhancing the specificity and precision of CRISPR/Cas9 delivery, biomaterials should be engineered to reduce off-target effects. This might include improving the design of the biomaterials or using improved CRISPR/Cas9 variants with increased selectivity (Feng et al., 2022; Iqbal et al., 2023).
2. Efficient Delivery: The successful delivery of particular cells, tissues, and organs for precise genome editing using CRISPR/Cas components is still a key barrier in gene therapy (Zou et al., 2022). High delivery efficiency must be attained to provide successful gene editing results. To comprehend the negative impacts of viral vectors, care should be taken while choosing the type of delivery vector. Getting CRISPR/Cas9 components to the target cells or tissues efficiently is one of the main problems. Biomaterials must be designed to effectively encapsulate and protect the CRISPR/Cas9 components during delivery, and also promote their efficient release and internalization into the target cells (Ramamoorth and Narvekar, 2015; Maeder and Gersbach, 2016; Asmamaw Mengstie, 2022).
3. Immunogenicity and Biocompatibility: To reduce negative immune reactions and increase their safety profile, CRISPR/Cas9 delivery biomaterials should be biocompatible and non-immunogenic. It is critical to carefully investigate the biocompatibility of the biomaterials and determine whether their usage may cause immunological responses, inflammation, or cytotoxicity (Behr et al., 2021; Dubey et al., 2022; Han et al., 2022a).
4. Targeting Specific Cell Types: For gene editing, biomaterials should be able to target just certain cell types or tissues. This necessitates the creation of targeting techniques that allow biomaterials to detect and attach to target cells with precision while minimizing off-target effects. To accomplish cell-specific targeting, particular ligands or antibodies can be added to the biomaterials (Deng et al., 2021; Zhuo et al., 2021).
5. Stability and Controlled Release: During storage and transit, biomaterials should retain their stability and safeguard the CRISPR/Cas9 components. For long-lasting gene editing effects, the regulated release of the CRISPR/Cas9 components from the biomaterials at the target region is crucial. It might be difficult to get the biomaterials to release kinetically and steadily in the right manner (Xu et al., 2020a; Huang et al., 2022a).
6. Scalability and Manufacturing: For clinical translation, it is crucial to develop scalable and reproducible manufacturing processes for biomaterials-mediated CRISPR/Cas9 delivery systems. Manufacturing considerations, such as scalability, cost-effectiveness, and quality control, need to be addressed to ensure consistent and reliable production of biomaterials (Abdeen and Saha, 2017; Abdeen et al., 2021).
7. Regulatory and Ethical Considerations: The administration of CRISPR/Cas9 through biomaterials raises legal and ethical concerns. Regulatory bodies need to conduct thorough safety analyses and compile supporting evidence before approving the use of biomaterials in clinical applications. It's crucial to thoroughly examine the moral dilemmas related to using gene editing technologies on individuals (Ayanoğlu et al., 2020; Kalidasan and Theva Das, 2021).

### 4.2 Future directions

1. Enhanced Delivery Efficiency: By shielding the genetic material from deterioration, encouraging cellular uptake, and aiding endosomal escape, biomaterials can increase the delivery effectiveness of CRISPR/Cas9 systems. To increase the effectiveness and specificity of CRISPR/Cas9 delivery, a variety of biomaterial-based delivery systems, including nanoparticles, liposomes, and hydrogels, have been investigated (Vargason et al., 2021; Bhattacharjee et al., 2022).
2. Tissue-Specific Targeting: The range of organs and cell types that genome-editing medicines can target will increase with the development of biomaterials that can distribute CRISPR components selectively. Currently, systemic distribution of LNPs to target hepatocytes in the liver is the most sophisticated method, although other studies have documented LNP delivery to non-hepatic organs such as the endothelium and immune cells (Swetha et al., 2023; Xu and Xia, 2023). Biomaterials can enhance the delivery efficiency of CRISPR/Cas9 systems by protecting the genetic material from degradation, promoting cellular absorption, and assisting endosomal escape. Numerous biomaterial-based delivery technologies, like hydrogels, nanoparticles, and liposomes, have been studied to improve the efficacy and specificity of CRISPR/Cas9 delivery (Ma and Mumper, 2013; Kim et al., 2020).
3. Long-Term Gene Editing: To offer overtime genetic modification and therapeutic advantages, biomaterials can allow the continuous release of CRISPR/Cas9 elements. Hydrogels or polymeric nanoparticles are examples of biodegradable biomaterials that may be made to release CRISPR/Cas9 components over a prolonged period to ensure continuous gene editing and therapeutic results (Hirakawa et al., 2020; Yang et al., 2023).
4. Immune Response Modulation: Gene-editing treatments can be made safer and less immunogenic by using biomaterials to modify the immune response to CRISPR/Cas9 delivery. Biomaterials can be modified on the surface and made with immunomodulatory substances to reduce immune cell activation and avoid unfavorable immunological reactions (Crudele and Chamberlin, 2018; Ewaisha and Anderson, 2023).
5. Clinical Translation: The delivery of CRISPR/Cas9 using biomaterials has a lot of potential for therapeutic use in the future. The creation of risk-free and efficient gene-editing medicines is being facilitated by developments in biomaterial design, formulation optimization, and safety evaluations. Preclinical research in animal models has shown encouraging findings, suggesting the possibility of clinical use in the future (Bashor et al., 2022; Yu et al., 2023b).

## 5 Conclusion

Delivering CRISPR/Cas9 using biomaterials has the potential to be a game-changing method for gene therapy for the treatment of infectious illnesses. With enhanced safety profiles, this cutting-edge technology combines the special benefits of CRISPR/Cas9 with biomaterials to provide accurate, efficient gene editing capabilities. To successfully distribute CRISPR/Cas9 components to the target cells and tissues during gene therapy, biomaterials play a crucial role in protecting the components (Foley et al., 2022). Encapsulating the genome-editing mechanism into biocompatible materials such as nanoparticles of lipids or viral vectors protects it from immunological processes and deterioration, improving its longevity and effectiveness. Investigators may build specialized systems for administration to address unique issues linked with certain genetic illnesses by using the plasticity of biomaterials, opening the door for individualized and optimal therapeutics (Musielak et al., 2022; Parambi et al., 2022).

Another potential method for battling viral illnesses is CRISPR/Cas9 delivery using biomaterials. Infectious agents may be rendered non-viable or less pathogenic by the targeted modification of the pathogen’s genomes using CRISPR/Cas9, greatly minimizing their influence on the wellbeing of humans (Dubey et al., 2022; Javed et al., 2023). Biomaterials enable efficient site-specific modification of genes by delivering CRISPR/Cas9 technology directly to infected cells while reducing the impact of off-target genes. This strategy may completely alter how viruses, bacteria, and other infectious agents are treated, paving the way for the creation of brand-new, more effective antiviral and antimicrobial treatments (Huang et al., 2023).

Nevertheless, given its enormous promise, CRISPR/Cas9 delivery via biomaterials has several difficulties that need to be resolved before it can be extensively used in clinical settings. Optimization of delivery effectiveness, reducing off-target effects, assuring long-term safety, and creating scalable and affordable production techniques for clinical-grade biomaterials and CRISPR/Cas9 components are some of the challenges involved. To successfully transfer this technology from the lab to the clinic, comprehensive preliminary and pre-clinical research and thorough safety evaluations are required (Liu et al., 2021d).

Finally, the delivery of CRISPR/Cas9 using biomaterials is a revolutionary development in the treatment of infectious diseases and gene therapy. Through the development of focused, accurate, and safe gene editing techniques, the combination of biomaterials and CRISPR/Cas9 has the potential to change medicine by tackling a variety of genetic illnesses and infectious diseases. As this field of study develops, CRISPR/Cas9 delivery via biomaterials may turn out to be a revolutionary therapeutic platform, opening up fresh opportunities for enhancing the wellbeing of humans and their quality of life.

## References

[B1] Abdeen A. A. Cosgrove B. D. Gersbach C. A. Saha K. (2021). Integrating biomaterials and genome editing approaches to advance biomedical science. Annu. Rev. Biomed. Eng. 23, 493–516. 10.1146/annurev-bioeng-122019-121602 33909475 PMC13087914

[B2] Abdeen A. A. Saha K. (2017). Manufacturing cell therapies using engineered biomaterials. Trends Biotechnol. 35 (10), 971–982. 10.1016/j.tibtech.2017.06.008 28711155 PMC5621598

[B3] Abdelnour S. A. Xie L. Hassanin A. A. Zuo E. Lu Y. (2021). The potential of CRISPR/Cas9 gene editing as a treatment strategy for inherited diseases. Front. Cell Dev. Biol. 9, 699597. 10.3389/fcell.2021.699597 34977000 PMC8715006

[B4] Abourehab M. A. S. Rajendran R. R. Singh A. Pramanik S. Shrivastav P. Ansari M. J. (2022). Alginate as a promising biopolymer in drug delivery and wound healing: A review of the state-of-the-art. Int. J. Mol. Sci. 23 (16), 9035. 10.3390/ijms23169035 36012297 PMC9409034

[B5] Abpeikar Z. Alizadeh A. A. Ahmadyousefi Y. Najafi A. A. Safaei M. (2022). Engineered cells along with smart scaffolds: Critical factors for improving tissue engineering approaches. Regen. Med. 17 (11), 855–876. 10.2217/rme-2022-0059 36065834

[B6] Afewerki S. Sheikhi A. Kannan S. Ahadian S. Khademhosseini A. (2018). Gelatin-polysaccharide composite scaffolds for 3D cell culture and tissue engineering: Towards natural therapeutics. Bioeng. Transl. Med. 4 (1), 96–115. 10.1002/btm2.10124 30680322 PMC6336672

[B7] Ahmadi S. E. Soleymani M. Shahriyary F. Ofoghi M. Fattahi M. D. Safa M. (2023). Viral vectors and extracellular vesicles: Innate delivery systems utilized in CRISPR/Cas-mediated cancer therapy. Cancer Gene Ther. 30 10.1038/s41417-023-00597-z PMC9971689 36854897

[B8] Alallam B. Altahhan S. Taher M. Mohd Nasir M. H. Doolaanea A. A. (2020). Electrosprayed alginate nanoparticles as CRISPR plasmid DNA delivery carrier: Preparation, optimization, and characterization. Pharm. (Basel, Switz. 13 (8), 158. 10.3390/ph13080158 PMC7465179 32707857

[B9] Alghamdi M. A. Fallica A. N. Virzì N. Kesharwani P. Pittalà V. Greish K. (2022). The promise of nanotechnology in personalized medicine. J. personalized Med. 12 (5), 673. 10.3390/jpm12050673 PMC9142986 35629095

[B10] Allemailem K. S. Alsahli M. A. Almatroudi A. Alrumaihi F. Alkhaleefah F. K. Rahmani A. H. (2022). Current updates of CRISPR/Cas9-mediated genome editing and targeting within tumor cells: An innovative strategy of cancer management. Cancer Commun. Lond. Engl. 42 (12), 1257–1287. 10.1002/cac2.12366 PMC9759771 36209487

[B11] Alves-Bezerra M. Furey N. Johnson C. G. Bissig K. D. (2019). Using CRISPR/Cas9 to model human liver disease. JHEP Rep. Innovation hepatology 1 (5), 392–402. 10.1016/j.jhepr.2019.09.002 PMC7005665 32039390

[B12] Andrée L. Oude Egberink R. Dodemont J. Hassani Besheli N. Yang F. Brock R. (2022). Gelatin nanoparticles for complexation and enhanced cellular delivery of mRNA. Nanomater. (Basel, Switz. 12 (19), 3423. 10.3390/nano12193423 PMC9565693 36234551

[B13] Armstrong J. P. K. Stevens M. M. (2019). Emerging technologies for tissue engineering: From gene editing to personalized medicine. Tissue Eng. 25(9-10), 688–692. 10.1089/ten.TEA.2019.0026 PMC6606435 30794069

[B14] Asmamaw Mengstie M. (2022). Viral vectors for the *in vivo* delivery of CRISPR components: Advances and challenges. Front. Bioeng. Biotechnol. 10, 895713. 10.3389/fbioe.2022.895713 35646852 PMC9133430

[B15] Asmamaw M. Zawdie B. (2021). Mechanism and applications of CRISPR/Cas-9-Mediated genome editing. Biol. Targets & Ther. 15, 353–361. 10.2147/BTT.S326422 PMC8388126 34456559

[B16] Ayanoğlu F. B. Elçin A. E. Elçin Y. M. (2020). Bioethical issues in genome editing by CRISPR-Cas9 technology. Turkish J. Biol. = Turk biyoloji dergisi 44 (2), 110–120. 10.3906/biy-1912-52 PMC7129066 32256147

[B17] Baci G. M. Cucu A. A. Giurgiu A. I. Muscă A. S. Bagameri L. Moise A. R. (2021). Advances in editing silkworms (*Bombyx mori*) genome by using the CRISPR-cas system. Insects 13 (1), 28. 10.3390/insects13010028 35055871 PMC8777690

[B18] Balon K. Sheriff A. Jacków J. Łaczmański Ł. (2022). Targeting cancer with CRISPR/Cas9-Based therapy. Int. J. Mol. Sci. 23 (1), 573. 10.3390/ijms23010573 35008996 PMC8745084

[B19] Barrangou R. Fremaux C. Deveau H. Richards M. Boyaval P. Moineau S. (2007). CRISPR provides acquired resistance against viruses in prokaryotes. Sci. (New York, N.Y.) 315 (5819), 1709–1712. 10.1126/science.1138140 17379808

[B20] Bashor C. J. Hilton I. B. Bandukwala H. Smith D. M. Veiseh O. (2022). Engineering the next generation of cell-based therapeutics. Nat. Rev. Drug Discov. 21 (9), 655–675. 10.1038/s41573-022-00476-6 35637318 PMC9149674

[B21] Behr M. Zhou J. Xu B. Zhang H. (2021). *In vivo* delivery of CRISPR/Cas9 therapeutics: Progress and challenges. Acta Pharm. Sin. B 11 (8), 2150–2171. 10.1016/j.apsb.2021.05.020 34522582 PMC8424283

[B22] Bengtsson N. E. Hall J. K. Odom G. L. Phelps M. P. Andrus C. R. Hawkins R. D. (2017). Muscle-specific CRISPR/Cas9 dystrophin gene editing ameliorates pathophysiology in a mouse model for Duchenne muscular dystrophy. Nat. Commun. 8, 14454. 10.1038/ncomms14454 28195574 PMC5316861

[B23] Bhattacharjee R. Jana A. Nandi A. Sinha A. Bhattacharjee A. Mitra S. (2022). The synergy of nanocarriers with CRISPR-Cas9 in an emerging technology platform for biomedical appliances: Current insights and perspectives. Mater. Des. 224, 111415. 10.1016/j.matdes.2022.111415

[B24] Binnie A. Fernandes E. Almeida-Lousada H. de Mello R. A. Castelo-Branco P. (2021). CRISPR-based strategies in infectious disease diagnosis and therapy. Infection 49 (3), 377–385. 10.1007/s15010-020-01554-w 33393066 PMC7779109

[B25] Boucard E. Vidal L. Coulon F. Mota C. Hascoët J. Y. Halary F. (2022). The degradation of gelatin/alginate/fibrin hydrogels is cell-type dependent and can be modulated by targeting fibrinolysis. Front. Bioeng. Biotechnol. 10, 920929. 10.3389/fbioe.2022.920929 35935486 PMC9355319

[B26] Bulbake U. Doppalapudi S. Kommineni N. Khan W. (2017). Liposomal formulations in clinical use: An updated review. Pharmaceutics 9 (2), 12. 10.3390/pharmaceutics9020012 28346375 PMC5489929

[B27] Bulcha J. T. Wang Y. Ma H. Tai P. W. L. Gao G. (2021). Viral vector platforms within the gene therapy landscape. Signal Transduct. Target. Ther. 6 (1), 53. 10.1038/s41392-021-00487-6 33558455 PMC7868676

[B28] Burnight E. R. Giacalone J. C. Cooke J. A. Thompson J. R. Bohrer L. R. Chirco K. R. (2018). CRISPR/Cas9 genome engineering: Treating inherited retinal degeneration. Prog. Retin. eye Res. 65, 28–49. 10.1016/j.preteyeres.2018.03.003 29578069 PMC8210531

[B29] Cai L. Fisher A. L. Huang H. Xie Z. (2016). CRISPR-mediated genome editing and human diseases. Genes & Dis. 3 (4), 244–251. 10.1016/j.gendis.2016.07.003 PMC6150104 30258895

[B30] Caliendo A. M. Gilbert D. N. Ginocchio C. C. Hanson K. E. May L. Quinn T. C. (2013). Better tests, better care: Improved diagnostics for infectious diseases. Clin. Infect. Dis. 57, S139–S170. 10.1093/cid/cit578 24200831 PMC3820169

[B31] Cao Y. Tan Y. F. Wong Y. S. Liew M. W. J. Venkatraman S. (2019). Recent advances in chitosan-based carriers for gene delivery. Mar. drugs 17 (6), 381. 10.3390/md17060381 31242678 PMC6627531

[B32] Caprifico A. E. Foot P. J. S. Polycarpou E. Calabrese G. (2022). Advances in chitosan-based CRISPR/Cas9 delivery systems. Pharmaceutics 14 (9), 1840. 10.3390/pharmaceutics14091840 36145588 PMC9505239

[B33] Carneiro S. P. Greco A. Chiesa E. Genta I. Merkel O. M. (2023). Shaping the future from the small scale: Dry powder inhalation of CRISPR/Cas9 lipid nanoparticles for the treatment of lung diseases. Expert Opin. drug Deliv. 20 (4), 471–487. 10.1080/17425247.2023.2185220 36896650 PMC7614984

[B34] Carreras A. Pane L. S. Nitsch R. Madeyski-Bengtson K. Porritt M. Akcakaya P. (2019). *In vivo* genome and base editing of a human PCSK9 knock-in hypercholesterolemic mouse model. BMC Biol. 17 (1), 4. 10.1186/s12915-018-0624-2 30646909 PMC6334452

[B35] Chadwick A. C. Musunuru K. (2017). Treatment of dyslipidemia using CRISPR/Cas9 genome editing. Curr. Atheroscler. Rep. 19 (7), 32. 10.1007/s11883-017-0668-8 28550381 PMC5832020

[B36] Chadwick A. C. Wang X. Musunuru K. (2017). Vivo base editing of PCSK9 (proprotein convertase subtilisin/kexin type 9) as a therapeutic alternative to genome editing. Arteriosclerosis, thrombosis, Vasc. Biol. 37 (9), 1741–1747. 10.1161/ATVBAHA.117.309881 PMC5570639 28751571

[B37] Chakraborty E. Sarkar D. (2022). Emerging therapies for hepatocellular carcinoma (HCC). Cancers 14 (11), 2798. 10.3390/cancers14112798 35681776 PMC9179883

[B38] Chan B. P. Leong K. W. (2008). Scaffolding in tissue engineering: General approaches and tissue-specific considerations. Eur. Spine J. 17, 467–479. 10.1007/s00586-008-0745-3 19005702 PMC2587658

[B39] Chang H. M. Wang Z. H. Luo H. N. Xu M. Ren X. Y. Zheng G. X. (2014). Poly(3-hydroxybutyrate-co-3-hydroxyhexanoate)-based scaffolds for tissue engineering. Braz. J. Med. Biol. Res. = Revista brasileira de pesquisas medicas e Biol. 47 (7), 533–539. 10.1590/1414-431x20143930 PMC4123831 25003631

[B40] Chen C. Zhong W. Du S. Li Y. Zeng Y. Liu K. (2022). Intelligent nanotherapeutic strategies for the delivery of CRISPR system. Acta Pharm. Sin. B. 13 10.1016/j.apsb.2022.12.013 PMC10326264 37425051

[B41] Chen G. Abdeen A. A. Wang Y. Shahi P. K. Robertson S. Xie R. (2019). A biodegradable nanocapsule delivers a Cas9 ribonucleoprotein complex for *in vivo* genome editing. Nat. Nanotechnol. 14 (10), 974–980. 10.1038/s41565-019-0539-2 31501532 PMC6778035

[B42] Chen G. Q. Jiang X. R. (2018). Engineering microorganisms for improving polyhydroxyalkanoate biosynthesis. Curr. Opin. Biotechnol. 53, 20–25. 10.1016/j.copbio.2017.10.008 29169056

[B43] Chen H. Wang L. Zeng X. Schwarz H. Nanda H. S. Peng X. (2021). Exosomes, a new star for targeted delivery. Front. Cell Dev. Biol. 9, 751079. 10.3389/fcell.2021.751079 34692704 PMC8531489

[B44] Chen Z. Liu F. Chen Y. Liu J. Wang X. Chen A. T. (2017). Targeted delivery of CRISPR/Cas9-Mediated cancer gene therapy via liposome-templated hydrogel nanoparticles. Adv. Funct. Mater. 27 (46), 1703036. 10.1002/adfm.201703036 29755309 PMC5939593

[B45] Cheng H. Zhang F. Ding Y. (2021). CRISPR/Cas9 delivery system engineering for genome editing in therapeutic applications. Pharmaceutics 13 (10), 1649. 10.3390/pharmaceutics13101649 34683943 PMC8538656

[B46] Cheng Q. Xia J. Wang K. Zhang Y. Chen Y. Zhong Q. (2022). CRISPR/Cas9 ribonucleoprotein (RNP) complex enables higher viability of transfected cells in genome editing of acute myeloid cells. Ann. Transl. Med. 10 (16), 862. 10.21037/atm-22-3279 36111017 PMC9469150

[B47] Cheung R. C. Ng T. B. Wong J. H. Chan W. Y. (2015). Chitosan: An update on potential biomedical and pharmaceutical applications. Mar. drugs 13 (8), 5156–5186. 10.3390/md13085156 26287217 PMC4557018

[B48] Chin J. S. Chooi W. H. Wang H. Ong W. Leong K. W. Chew S. Y. (2019). A scaffold-mediated non-viral delivery platform for CRISPR/Cas9-based genome editing. Acta biomater. 90, 60–70. 10.1016/j.actbio.2019.04.020 30978509

[B49] Cho E. Y. Ryu J. Y. Lee H. A. R. Hong S. H. Park H. S. Hong K. S. (2019). Lecithin nano-liposomal particle as a CRISPR/Cas9 complex delivery system for treating type 2 diabetes. J. Nanobiotechnology 17 (1), 19. 10.1186/s12951-019-0452-8 30696428 PMC6350399

[B50] Chong Z. X. Yeap S. K. Ho W. Y. (2021). Transfection types, methods, and strategies: A technical review. PeerJ 9, e11165. 10.7717/peerj.11165 33976969 PMC8067914

[B51] Chooi W. H. Chin J. S. Chew S. Y. (2021). Scaffold-based delivery of CRISPR/Cas9 ribonucleoproteins for genome editing. Methods Mol. Biol. Clift. N.J.) 2211, 183–191. 10.1007/978-1-0716-0943-9_13 33336278

[B52] Clément F. Grockowiak E. Zylbersztejn F. Fossard G. Gobert S. Maguer-Satta V. (2017). Stem cell manipulation, gene therapy, and the risk of cancer stem cell emergence. Stem Cell Investig. 4, 67. 10.21037/sci.2017.07.03 PMC5539392 28815178

[B53] Cong L. Ran F. A. Cox D. Lin S. Barretto R. Habib N. (2013). Multiplex genome engineering using CRISPR/Cas systems. Sci. (New York, N.Y.) 339 (6121), 819–823. 10.1126/science.1231143 PMC3795411 23287718

[B54] Cota-Coronado A. Díaz-Martínez N. F. Padilla-Camberos E. Díaz-Martínez N. E. (2019). Editing the central nervous system through CRISPR/Cas9 systems. Front. Mol. Neurosci. 12, 110. 10.3389/fnmol.2019.00110 31191241 PMC6546027

[B55] Cribbs A. P. Perera S. M. W. (2017). Science and bioethics of CRISPR/Cas9 gene editing: An analysis towards separating facts and fiction. Yale J. Biol. Med. 90 (4), 625–634. 29259526 PMC5733851

[B56] Crudele J. M. Chamberlain J. S. (2018). Cas9 immunity creates challenges for CRISPR gene editing therapies. Nat. Commun. 9 (1), 3497. 10.1038/s41467-018-05843-9 30158648 PMC6115392

[B57] Cyranoski D. (2016). CRISPR gene-editing tested in a person for the first time. Nature 539 (7630), 479. 10.1038/nature.2016.20988 27882996

[B58] Danisovic L. Culenova M. Csobonyeiova M. (2018). Induced pluripotent stem cells for Duchenne muscular dystrophy modeling and therapy. Cells 7 (12), 253. 10.3390/cells7120253 30544588 PMC6315586

[B59] De Matteis L. Martín-Rapún R. de la Fuente J. M. (2018). Nanotechnology in personalized medicine: A promising tool for alzheimer's disease treatment. Curr. Med. Chem. 25 (35), 4602–4615. 10.2174/0929867324666171012112026 29022501

[B60] Deltcheva E. Chylinski K. Sharma C. M. Gonzales K. Chao Y. Pirzada Z. A. (2011). CRISPR RNA maturation by trans-encoded small RNA and host factor RNase III. Nature 471 (7340), 602–607. 10.1038/nature09886 21455174 PMC3070239

[B61] Deng J. Zhang J. Shi K. Liu Z. (2022). Drug development progress in Duchenne muscular dystrophy. Front. Pharmacol. 13, 950651. 10.3389/fphar.2022.950651 35935842 PMC9353054

[B62] Deng Z. Kalin G. T. Shi D. Kalinichenko V. V. (2021). Nanoparticle delivery systems with cell-specific targeting for pulmonary diseases. Am. J. Respir. Cell Mol. Biol. 64 (3), 292–307. 10.1165/rcmb.2020-0306TR 33095997 PMC7909340

[B63] Deol P. Madhwal A. Sharma G. Kaushik R. Malik Y. S. (2022). CRISPR use in diagnosis and therapy for COVID-19. Methods Microbiol. 50, 123–150. 10.1016/bs.mim.2022.03.002 PMC9073596 38013928

[B64] Ding J. Wang J. Chen J. (2021). Exosomes as therapeutic vehicles in liver diseases. Ann. Transl. Med. 9 (8), 735. 10.21037/atm-20-5422 33987433 PMC8106083

[B65] Ding Q. Strong A. Patel K. M. Ng S. L. Gosis B. S. Regan S. N. (2014). Permanent alteration of PCSK9 with *in vivo* CRISPR/Cas9 genome editing. Circulation Res. 115 (5), 488–492. 10.1161/CIRCRESAHA.115.304351 24916110 PMC4134749

[B66] Dong C. Lv Y. (2016). Application of collagen scaffold in tissue engineering: Recent advances and new perspectives. Polymers 8 (2), 42. 10.3390/polym8020042 30979136 PMC6432532

[B67] Driehuis E. Clevers H. (2017). CRISPR/Cas 9 genome editing and its applications in organoids. Am. J. Physiology. Gastrointest. liver physiology 312(3), G257–G265. 10.1152/ajpgi.00410.2016 28126704

[B68] du Rand A. Hunt J. M. T. Feisst V. Sheppard H. M. (2022). Epidermolysis bullosa: A review of the tissue-engineered skin substitutes used to treat wounds. Mol. diagnosis Ther. 26 (6), 627–643. 10.1007/s40291-022-00613-2 PMC9626425 36251245

[B69] Duan C. Cao H. Zhang L. H. Xu Z. (2021a). Harnessing the CRISPR-cas systems to combat antimicrobial resistance. Front. Microbiol. 12, 716064. 10.3389/fmicb.2021.716064 34489905 PMC8418092

[B70] Duan L. Ouyang K. Xu X. Xu L. Wen C. Zhou X. (2021b). Nanoparticle delivery of CRISPR/Cas9 for genome editing. Front. Genet. 12, 673286. 10.3389/fgene.2021.673286 34054927 PMC8149999

[B71] Dubey A. K. Kumar Gupta V. Kujawska M. Orive G. Kim N. Y. Li C. Z. (2022). Exploring nano-enabled CRISPR-Cas-powered strategies for efficient diagnostics and treatment of infectious diseases. J. nanostructure Chem. 12, 1–32. 10.1007/s40097-022-00472-7 PMC8853211 35194511

[B72] Dumas M. P. Xia S. Bear C. E. Ratjen F. (2021). Perspectives on the translation of *in-vitro* studies to precision medicine in Cystic Fibrosis. EBioMedicine 73, 103660. 10.1016/j.ebiom.2021.103660 34740114 PMC8577330

[B73] Dumontel B. Conejo-Rodríguez V. Vallet-Regí M. Manzano M. (2023). Natural biopolymers as smart coating materials of mesoporous silica nanoparticles for drug delivery. Pharmaceutics 15 (2), 447. 10.3390/pharmaceutics15020447 36839771 PMC9965229

[B74] Dzobo K. Thomford N. E. Senthebane D. A. Shipanga H. Rowe A. Dandara C. (2018). Advances in regenerative medicine and tissue engineering: Innovation and transformation of medicine. Stem Cells Int. 2018, 2495848. 10.1155/2018/2495848 30154861 PMC6091336

[B75] El-Kenawy A. Benarba B. Neves A. F. de Araujo T. G. Tan B. L. Gouri A. (2019). Gene surgery: Potential applications for human diseases. EXCLI J. 18, 908–930. 10.17179/excli2019-1833 31762718 PMC6868916

[B76] Erkut E. Yokota T. (2022). CRISPR therapeutics for Duchenne muscular dystrophy. Int. J. Mol. Sci. 23 (3), 1832. 10.3390/ijms23031832 35163754 PMC8836469

[B77] Esser T. U. Trossmann V. T. Lentz S. Engel F. B. Scheibel T. (2021). Designing of spider silk proteins for human induced pluripotent stem cell-based cardiac tissue engineering. Mater. today 11, 100114. 10.1016/j.mtbio.2021.100114 PMC8209670 34169268

[B78] Ewaisha R. Anderson K. S. (2023). Immunogenicity of CRISPR therapeutics-Critical considerations for clinical translation. Front. Bioeng. Biotechnol. 11, 1138596. 10.3389/fbioe.2023.1138596 36873375 PMC9978118

[B79] Fang T. Cao X. Ibnat M. Chen G. (2022). Stimuli-responsive nanoformulations for CRISPR/Cas9 genome editing. J. Nanobiotechnology 20 (1), 354. 10.1186/s12951-022-01570-y 35918694 PMC9344766

[B80] Farheen J. Hosmane N. S. Zhao R. Zhao Q. Iqbal M. Z. Kong X. (2022). Nanomaterial-assisted CRISPR gene-engineering - a hallmark for triple-negative breast cancer therapeutics advancement. Mater. Today. Bio 16, 100450. 10.1016/j.mtbio.2022.100450 PMC9576993 36267139

[B81] Feng S. Wang Z. Li A. Xie X. Liu J. Li S. (2022). Strategies for high-efficiency mutation using the CRISPR/Cas system. Front. Cell Dev. Biol. 9, 803252. 10.3389/fcell.2021.803252 35198566 PMC8860194

[B82] Ferracini R. Martínez Herreros I. Russo A. Casalini T. Rossi F. Perale G. (2018). Scaffolds as structural tools for bone-targeted drug delivery. Pharmaceutics 10 (3), 122. 10.3390/pharmaceutics10030122 30096765 PMC6161191

[B83] Fichter K. M. Setayesh T. Malik P. (2023). Strategies for precise gene edits in mammalian cells. Molecular therapy. Nucleic acids. 32, 536–552. 10.1016/j.omtn.2023.04.012 37215153 PMC10192336

[B84] Finn J. D. Smith A. R. Patel M. C. Shaw L. Youniss M. R. van Heteren J. (2018). A single administration of CRISPR/Cas9 lipid nanoparticles achieves robust and persistent *in vivo* genome editing. Cell Rep. 22 (9), 2227–2235. 10.1016/j.celrep.2018.02.014 29490262

[B85] Foley R. A. Sims R. A. Duggan E. C. Olmedo J. K. Ma R. Jonas S. J. (2022). Delivering the CRISPR/Cas9 system for engineering gene therapies: Recent cargo and delivery approaches for clinical translation. Front. Bioeng. Biotechnol. 10, 973326. 10.3389/fbioe.2022.973326 36225598 PMC9549251

[B86] Frangoul H. Altshuler D. Cappellini M. D. Chen Y. S. Domm J. Eustace B. K. (2021). CRISPR-Cas9 gene editing for sickle cell disease and β-thalassemia. N. Engl. J. Med. 384 (3), 252–260. 10.1056/NEJMoa2031054 33283989

[B87] Freen-van Heeren J. J. (2022). Closing the door with CRISPR: Genome editing of CCR5 and CXCR4 as a potential curative solution for HIV. BioTech 11 (3), 25. 10.3390/biotech11030025 35892930 PMC9326690

[B88] Fuoco C. Petrilli L. L. Cannata S. Gargioli C. (2016). Matrix scaffolding for stem cell guidance toward skeletal muscle tissue engineering. J. Orthop. Surg. Res. 11 (1), 86. 10.1186/s13018-016-0421-y 27460672 PMC4962357

[B89] Fus-Kujawa A. Prus P. Bajdak-Rusinek K. Teper P. Gawron K. Kowalczuk A. (2021). An overview of methods and tools for transfection of eukaryotic cells *in vitro* . Front. Bioeng. Biotechnol. 9, 701031. 10.3389/fbioe.2021.701031 34354988 PMC8330802

[B90] Gaj T. Sirk S. J. Shui S. L. Liu J. (2016). Genome-editing technologies: Principles and applications. Cold Spring Harb. Perspect. Biol. 8 (12), a023754. 10.1101/cshperspect.a023754 27908936 PMC5131771

[B91] Gao Q. Dong X. Xu Q. Zhu L. Wang F. Hou Y. (2019). Therapeutic potential of CRISPR/Cas9 gene editing in engineered T-cell therapy. Cancer Med. 8 (9), 4254–4264. 10.1002/cam4.2257 31199589 PMC6675705

[B92] Gelse K. Pöschl E. Aigner T. (2003). Collagens--structure, function, and biosynthesis. Adv. drug Deliv. Rev. 55 (12), 1531–1546. 10.1016/j.addr.2003.08.002 14623400

[B93] Getahun Y. A. Ali D. A. Taye B. W. Alemayehu Y. A. (2022). Multidrug-resistant microbial therapy using antimicrobial peptides and the CRISPR/Cas9 system. Veterinary Med. Auckl. N.Z.) 13, 173–190. 10.2147/VMRR.S366533 PMC9379109 35983086

[B94] Ghadi A. Mahjoub S. Tabandeh F. Talebnia F. (2014). Synthesis and optimization of chitosan nanoparticles: Potential applications in nanomedicine and biomedical engineering. Casp. J. Intern. Med. 5 (3), 156–161. PMC4143737 25202443

[B95] Gheorghita R. Anchidin-Norocel L. Filip R. Dimian M. Covasa M. (2021). Applications of biopolymers for drugs and probiotics delivery. Polymers 13 (16), 2729. 10.3390/polym13162729 34451268 PMC8399127

[B96] Gholizadeh P. Köse Ş. Dao S. Ganbarov K. Tanomand A. Dal T. (2020). How CRISPR-cas system could Be used to combat antimicrobial resistance. Infect. drug Resist. 13, 1111–1121. 10.2147/IDR.S247271 32368102 PMC7182461

[B97] Gillmore J. D. Gane E. Taubel J. Kao J. Fontana M. Maitland M. L. (2021). CRISPR-Cas9 *in vivo* gene editing for transthyretin amyloidosis. N. Engl. J. Med. 385 (6), 493–502. 10.1056/NEJMoa2107454 34215024

[B98] Givens B. E. Naguib Y. W. Geary S. M. Devor E. J. Salem A. K. (2018). Nanoparticle-based delivery of CRISPR/Cas9 genome-editing therapeutics. AAPS J. 20 (6), 108. 10.1208/s12248-018-0267-9 30306365 PMC6398936

[B99] Goetz L. H. Schork N. J. (2018). Personalized medicine: Motivation, challenges, and progress. Fertil. Steril. 109 (6), 952–963. 10.1016/j.fertnstert.2018.05.006 29935653 PMC6366451

[B100] Gopal S. Rodrigues A. L. Dordick J. S. (2020). Exploiting CRISPR Cas9 in three-dimensional stem cell cultures to model disease. Front. Bioeng. Biotechnol. 8, 692. 10.3389/fbioe.2020.00692 32671050 PMC7326781

[B101] Gostimskaya I. (2022). CRISPR-Cas9: A history of its discovery and ethical considerations of its use in genome editing. Biochemistry 87(8), 777–788. 10.1134/S0006297922080090 36171658 PMC9377665

[B102] Grath A. Dai G. (2019). Direct cell reprogramming for tissue engineering and regenerative medicine. J. Biol. Eng. 13, 14. 10.1186/s13036-019-0144-9 30805026 PMC6373087

[B103] Guo C. Ma X. Gao F. Guo Y. (2023). Off-target effects in CRISPR/Cas9 gene editing. Front. Bioeng. Biotechnol. 11, 1143157. 10.3389/fbioe.2023.1143157 36970624 PMC10034092

[B104] Guo H. Huang X. (2022). Engineered exosomes for future gene-editing therapy. Biomater. Transl. 3 (4), 240–242. 10.12336/biomatertransl.2022.04.003 36846508 PMC9947735

[B105] Guo P. Liu D. Subramanyam K. Wang B. Yang J. Huang J. (2018). Nanoparticle elasticity directs tumor uptake. Nat. Commun. 9 (1), 130. 10.1038/s41467-017-02588-9 29317633 PMC5760638

[B106] Guzik M. W. (2021). Polyhydroxyalkanoates, bacterially synthesized polymers, as a source of chemical compounds for the synthesis of advanced materials and bioactive molecules. Appl. Microbiol. Biotechnol. 105 (20), 7555–7566. 10.1007/s00253-021-11589-0 34536102 PMC8502142

[B107] Han J. P. Kim M. Choi B. S. Lee J. H. Lee G. S. Jeong M. (2022a). *In vivo*, delivery of CRISPR/Cas9 using lipid nanoparticles enables antithrombin gene editing for sustainable hemophilia A and B therapy. Sci. Adv. 8 (3), eabj6901. 10.1126/sciadv.abj6901 35061543 PMC8782450

[B108] Han X. Alu A. Liu H. Shi Y. Wei X. Cai L. (2022b). Biomaterial-assisted biotherapy: A brief review of biomaterials used in drug delivery, vaccine development, gene therapy, and stem cell therapy. Bioact. Mater. 17, 29–48. 10.1016/j.bioactmat.2022.01.011 35386442 PMC8958282

[B109] Haney M. J. Klyachko N. L. Zhao Y. Gupta R. Plotnikova E. G. He Z. (2015). Exosomes as drug delivery vehicles for Parkinson's disease therapy. J. Control. release official J. Control. Release Soc. 207, 18–30. 10.1016/j.jconrel.2015.03.033 PMC4430381 25836593

[B110] Hazafa A. Mumtaz M. Farooq M. F. Bilal S. Chaudhry S. N. Firdous M. (2020). CRISPR/Cas9: A powerful genome editing technique for the treatment of cancer cells with present challenges and future directions. Life Sci. 263, 118525. 10.1016/j.lfs.2020.118525 33031826 PMC7533657

[B111] Herdiana Y. Wathoni N. Shamsuddin S. Joni I. M. Muchtaridi M. (2021a). Chitosan-based nanoparticles of targeted drug delivery system in breast cancer treatment. Polymers 13 (11), 1717. 10.3390/polym13111717 34074020 PMC8197416

[B112] Herdiana Y. Wathoni N. Shamsuddin S. Muchtaridi M. (2021b). Drug release study of the chitosan-based nanoparticles. Heliyon 8 (1), e08674. 10.1016/j.heliyon.2021.e08674 35028457 PMC8741465

[B113] Hirakawa M. P. Krishnakumar R. Timlin J. A. Carney J. P. Butler K. S. (2020). Gene editing and CRISPR in the clinic: Current and future perspectives. Biosci. Rep. 40 (4), BSR20200127. 10.1042/BSR20200127 32207531 PMC7146048

[B114] Ho B. X. Loh S. J. H. Chan W. K. Soh B. S. (2018). *In vivo* genome editing as a therapeutic approach. Int. J. Mol. Sci. 19 (9), 2721. 10.3390/ijms19092721 30213032 PMC6163904

[B115] Ho T. C. Kim H. S. Chen Y. Li Y. LaMere M. W. Chen C. (2021). Scaffold-mediated CRISPR/Cas9 delivery system for acute myeloid leukemia therapy. Sci. Adv. 7 (21), eabg3217. 10.1126/sciadv.abg3217 34138728 PMC8133753

[B116] Hoare T. R. Kohane D. S. (2008). Hydrogels in drug delivery: Progress and challenges. Polymer 49 (8), 1993–2007. 10.1016/j.polymer.2008.01.027

[B117] Hong S. Choi D. W. Kim H. N. Park C. G. Lee W. Park H. H. (2020). Protein-based nanoparticles as drug delivery systems. Pharmaceutics 12 (7), 604. 10.3390/pharmaceutics12070604 32610448 PMC7407889

[B118] Hou P. Chen S. Wang S. Yu X. Chen Y. Jiang M. (2015). Genome editing of CXCR4 by CRISPR/Cas9 confers cells resistant to HIV-1 infection. Sci. Rep. 5, 15577. 10.1038/srep15577 26481100 PMC4612538

[B119] Howard D. Buttery L. D. Shakesheff K. M. Roberts S. J. (2008). Tissue engineering: Strategies, stem cells and scaffolds. J. Anat. 213 (1), 66–72. 10.1111/j.1469-7580.2008.00878.x 18422523 PMC2475566

[B120] Hsu P. D. Lander E. S. Zhang F. (2014). Development and applications of CRISPR/Cas9 for genome engineering. Cell 157 (6), 1262–1278. 10.1016/j.cell.2014.05.010 24906146 PMC4343198

[B121] Huang G. Liu Y. Chen L. (2017). Chitosan and its derivatives as vehicles for drug delivery. Drug Deliv. 24 (1), 108–113. 10.1080/10717544.2017.1399305 29124981 PMC8812576

[B122] Huang J. Zhou Y. Li J. Lu A. Liang C. (2022a). CRISPR/Cas systems: Delivery and application in gene therapy. Front. Bioeng. Biotechnol. 10, 942325. 10.3389/fbioe.2022.942325 36483767 PMC9723151

[B123] Huang K. Zapata D. Tang Y. Teng Y. Li Y. (2022b). Vivo delivery of CRISPR-Cas9 genome editing components for therapeutic applications. Biomaterials 291, 121876. 10.1016/j.biomaterials.2022.121876 36334354 PMC10018374

[B124] Huang W. Ling S. Li C. Omenetto F. G. Kaplan D. L. (2018). Silkworm silk-based materials and devices generated using bio-nanotechnology. Chem. Soc. Rev. 47 (17), 6486–6504. 10.1039/c8cs00187a 29938722 PMC6113080

[B125] Huang X. Li A. Xu P. Yu Y. Li S. Hu L. (2023). Current and prospective strategies for advancing the targeted delivery of CRISPR/Cas system via extracellular vesicles. J. Nanobiotechnology 21 (1), 184. 10.1186/s12951-023-01952-w 37291577 PMC10249948

[B126] Hwang C. M. Sant S. Masaeli M. Kachouie N. N. Zamanian B. Lee S. H. (2010). Fabrication of three-dimensional porous cell-laden hydrogel for tissue engineering. Biofabrication 2 (3), 035003. 10.1088/1758-5082/2/3/035003 20823504 PMC3282162

[B127] Imamoğlu R. Kaplan Ö. Gök M. K. Gökçe İ. (2022). Polymer-based transfection agents used in CRISPR/CAS9 system. Türk Doğa ve Fen Derg. 11 (1), 151–156. 10.46810/tdfd.795053

[B128] Ikada Y. (2006). Challenges in tissue engineering. J. R. Soc. Interface 3 (10), 589–601. 10.1098/rsif.2006.0124 16971328 PMC1664655

[B129] Iqbal Z. Rehman K. Xia J. Shabbir M. Zaman M. Liang Y. (2023). Biomaterial-assisted targeted and controlled delivery of CRISPR/Cas9 for precise gene editing. Biomaterials Sci. 2023. 10.1039/d2bm01636b 37102700

[B130] Ishino Y. Shinagawa H. Makino K. Amemura M. Nakata A. (1987). Nucleotide sequence of the iap gene, responsible for alkaline phosphatase isozyme conversion in *Escherichia coli*, and identification of the gene product. J. Bacteriol. 169 (12), 5429–5433. 10.1128/jb.169.12.5429-5433.1987 3316184 PMC213968

[B131] Ishizu N. Yui D. Hebisawa A. Aizawa H. Cui W. Fujita Y. (2016). Impaired striatal dopamine release in homozygous Vps35 D620N knock-in mice. Hum. Mol. Genet. 25 (20), 4507–4517. 10.1093/hmg/ddw279 28173004

[B132] Iwasaki R. S. Ozdilek B. A. Garst A. D. Choudhury A. Batey R. T. (2020). Small molecule-regulated sgRNAs enable control of genome editing in *E. coli* by Cas9. Nat. Commun. 11 (1), 1394. 10.1038/s41467-020-15226-8 32170140 PMC7070018

[B133] Jacinto F. V. Link W. Ferreira B. I. (2020). CRISPR/Cas9-mediated genome editing: From basic research to translational medicine. J. Cell. Mol. Med. 24 (7), 3766–3778. 10.1111/jcmm.14916 32096600 PMC7171402

[B134] Jacków J. Guo Z. Hansen C. Abaci H. E. Doucet Y. S. Shin J. U. (2019). CRISPR/Cas9-based targeted genome editing for correction of recessive dystrophic epidermolysis bullosa using IPS cells. Proc. Natl. Acad. Sci. 116 (52), 26846–26852. 10.1073/pnas.1907081116 31818947 PMC6936361

[B135] Jang J. H. Bengali Z. Houchin T. L. Shea L. D. (2006). Surface adsorption of DNA to tissue engineering scaffolds for efficient gene delivery. J. Biomed. Mater. Res. Part A 77 (1), 50–58. 10.1002/jbm.a.30643 PMC2648387 16353173

[B136] Javaid D. Ganie S. Y. Hajam Y. A. (2022). CRISPR/Cas9 system: A reliable and facile genome editing tool in modern biology. Mol. Biol. Rep. 49, 12133–12150. 10.1007/s11033-022-07880-6 36030476 PMC9420241

[B137] Javed M. U. Hayat M. T. Mukhtar H. Imre K. (2023). CRISPR-Cas9 system: A prospective pathway toward combatting antibiotic resistance. Antibiot. (Basel, Switz. 12 (6), 1075. 10.3390/antibiotics12061075 PMC10295005 37370394

[B138] Jiang F. Doudna J. A. (2017). CRISPR-Cas9 structures and mechanisms. Annu. Rev. biophysics 46, 505–529. 10.1146/annurev-biophys-062215-010822 28375731

[B139] Jie W. Dong W. Yixian Z. (2021). Synthesis and biopharmaceutical applications of sugar-based polymers: New advances and future prospects. ACS Biomaterials Sci. Eng. 7 (3). 10.1021/acsbiomaterials.0c01710 33523642

[B140] Jinek M. Chylinski K. Fonfara I. Hauer M. Doudna J. A. Charpentier E. (2012). A programmable dual-RNA-guided DNA endonuclease in adaptive bacterial immunity. Sci. (New York, N.Y.) 337 (6096), 816–821. 10.1126/science.1225829 PMC6286148 22745249

[B141] Joseph T. M. Kar Mahapatra D. Esmaeili A. Piszczyk Ł. Hasanin M. S. Kattali M. (2023). Nanoparticles: Taking a unique position in medicine. Nanomaterials 13 (3), 574. 10.3390/nano13030574 36770535 PMC9920911

[B142] Jubair L. Lam A. K. Fallaha S. McMillan N. A. J. (2021). CRISPR/Cas9-loaded stealth liposomes effectively cleared established HPV16-driven tumors in syngeneic mice. PloS one 16 (1), e0223288. 10.1371/journal.pone.0223288 33411765 PMC7790238

[B143] Jung H. N. Lee S. Y. Lee S. Youn H. Im H. J. (2022). Lipid nanoparticles for delivery of RNA therapeutics: Current status and the role of *in vivo* imaging. Theranostics 12 (17), 7509–7531. 10.7150/thno.77259 36438494 PMC9691360

[B144] Kabwe J. C. Sawada H. Mitani Y. Oshita H. Tsuboya N. Zhang E. (2022). CRISPR-mediated Bmpr2 point mutation exacerbates late pulmonary vasculopathy and reduces survival in rats with experimental pulmonary hypertension. Respir. Res. 23 (1), 87. 10.1186/s12931-022-02005-w 35395852 PMC8994407

[B145] Kalidasan V. Theva Das K. (2021). Is Malaysia ready for human gene editing: A regulatory, biosafety and biosecurity perspective? Front. Bioeng. Biotechnol. 9, 649203. 10.3389/fbioe.2021.649203 33777918 PMC7992004

[B146] Kang K. Song Y. Kim I. Kim T.-J. (2022). Therapeutic applications of the CRISPR-cas system. Bioengineering 9 (9), 477. 10.3390/bioengineering9090477 36135023 PMC9495783

[B147] Katti A. Diaz B. J. Caragine C. M. Sanjana N. E. Dow L. E. (2022). CRISPR in cancer biology and therapy. Nat. Rev. Cancer 22 (5), 259–279. 10.1038/s41568-022-00441-w 35194172

[B148] Kaushik I. Ramachandran S. Srivastava S. K. (2019). CRISPR/Cas9: A multifaceted therapeutic strategy for cancer treatment. Seminars Cell & Dev. Biol. 96, 4–12. 10.1016/j.semcdb.2019.04.018 PMC6829064 31054324

[B149] Kelkar S. S. Hill T. K. Marini F. C. Mohs A. M. (2016). Near-infrared fluorescent nanoparticles based on hyaluronic acid: Self-assembly, optical properties, and cell interaction. Acta biomater. 36, 112–121. 10.1016/j.actbio.2016.03.024 26995504 PMC4846482

[B150] Khademi Z. Ramezani M. Alibolandi M. Zirak M. R. Salmasi Z. Abnous K. (2022). A novel dual-targeting delivery system for specific delivery of CRISPR/Cas9 using hyaluronic acid, chitosan, and AS1411. Carbohydr. Polym. 292, 119691. 10.1016/j.carbpol.2022.119691 35725215

[B151] Khalil A. M. (2020). The genome editing revolution: Review. J. Genet. Eng. Biotechnol. 18 (1), 68. 10.1186/s43141-020-00078-y 33123803 PMC7596157

[B152] Khwatenge C. N. Nahashon S. N. (2021). Recent advances in the application of CRISPR/Cas9 gene editing system in poultry species. Front. Genet. 12, 627714. 10.3389/fgene.2021.627714 33679892 PMC7933658

[B153] Kılıçay E. Demirbilek M. Türk M. Güven E. Hazer B. Denkbas E. B. (2011). Preparation and characterization of poly (3-hydroxybutyrate-co-3-hydroxy hexanoate) (PHBHHX) based nanoparticles for targeted cancer therapy. Eur. J. Pharm. Sci. 44 (3), 310–320. 10.1016/j.ejps.2011.08.013 21884788

[B154] Kim D. Le Q. V. Wu Y. Park J. Oh Y. K. (2020a). Nanovesicle-mediated delivery systems for CRISPR/Cas genome editing. Pharmaceutics 12 (12), 1233. 10.3390/pharmaceutics12121233 33353099 PMC7766488

[B155] Kim J. Koo B. K. Knoblich J. A. (2020b). Human organoids: Model systems for human biology and medicine. Nat. Rev. Mol. Cell Biol. 21 (10), 571–584. 10.1038/s41580-020-0259-3 32636524 PMC7339799

[B156] Kim S. Hupperetz C. Lim S. Kim C. H. (2021). Genome editing of immune cells using CRISPR/Cas9. BMB Rep. 54 (1), 59–69. 10.5483/BMBRep.2021.54.1.245 33298251 PMC7851445

[B157] Kim T. Lu T. K. (2019). CRISPR/Cas-based devices for mammalian synthetic biology. Curr. Opin. Chem. Biol. 52, 23–30. 10.1016/j.cbpa.2019.04.015 31136835

[B158] Kim W. Lee S. Kim H. S. Song M. Cha Y. H. Kim Y. H. (2018). Targeting mutant KRAS with CRISPR/Cas9 controls tumor growth. Genome Res. 28 (3), 374–382. 10.1101/gr.223891.117 29326299 PMC5848616

[B159] Kim Y. S. Guilak F. (2022). Engineering hyaluronic acid for the development of new treatment strategies for osteoarthritis. Int. J. Mol. Sci. 23 (15), 8662. 10.3390/ijms23158662 35955795 PMC9369020

[B160] Kochhar D. DeBari M. K. Abbott R. D. (2021). The materiobiology of silk: Exploring the biophysical influence of silk biomaterials on directing cellular behaviors. Front. Bioeng. Biotechnol. 9, 697981. 10.3389/fbioe.2021.697981 34239865 PMC8259510

[B161] Komor A. C. Badran A. H. Liu D. R. (2018). Editing the genome without double-stranded DNA breaks. ACS Chem. Biol. 13 (2), 383–388. 10.1021/acschembio.7b00710 28957631 PMC5891729

[B162] Kong H. Ju E. Yi K. Xu W. Lao Y. H. Cheng D. (2021). Advanced nanotheranostics of CRISPR/Cas for viral hepatitis and hepatocellular carcinoma. Adv. Sci. (Weinheim, Baden-Wurttemberg, Ger. 8 (24), e2102051. 10.1002/advs.202102051 PMC8693080 34665528

[B163] Kooijmans S. A. A. Stremersch S. Braeckmans K. de Smedt S. C. Hendrix A. Wood M. J. A. (2013). Electroporation-induced siRNA precipitation obscures the efficiency of siRNA loading into extracellular vesicles. J. Control. release official J. Control. Release Soc. 172 (1), 229–238. 10.1016/j.jconrel.2013.08.014 23994516

[B164] Kosicki M. Allen F. Steward F. Tomberg K. Pan Y. Bradley A. (2022). Cas9-induced large deletions and small indels are controlled in a convergent fashion. Nat. Commun. 13 (1), 3422. 10.1038/s41467-022-30480-8 35701408 PMC9197861

[B165] Krishna L. Dhamodaran K. Jayadev C. (2016). Nanostructured scaffold as a determinant of stem cell fate. Stem Cell Res. Ther. 7, 188. 10.1186/s13287-016-0440-y 28038681 PMC5203716

[B166] Lamichhane T. N. Jeyaram A. Patel D. B. Parajuli B. Livingston N. K. Arumugasaamy N. (2016). Oncogene knockdown via active loading of small RNAs into extracellular vesicles by sonication. Cell. Mol. Bioeng. 9 (3), 315–324. 10.1007/s12195-016-0457-4 27800035 PMC5084850

[B167] Lapinaite A. Knott G. J. Palumbo C. M. Lin-Shiao E. Richter M. F. Zhao K. T. (2020). DNA capture by a CRISPR-Cas9-guided adenine base editor. Sci. (New York, N.Y.) 369 (6503), 566–571. 10.1126/science.abb1390 PMC8598131 32732424

[B168] Lawhorn I. E. Ferreira J. P. Wang C. L. (2014). Evaluation of sgRNA target sites for CRISPR-mediated repression of TP53. PloS one 9 (11), e113232. 10.1371/journal.pone.0113232 25398078 PMC4232525

[B169] Lee K. Y. Mooney D. J. (2012). Alginate: Properties and biomedical applications. Prog. Polym. Sci. 37 (1), 106–126. 10.1016/j.progpolymsci.2011.06.003 22125349 PMC3223967

[B170] Lee S. Y. (1996). Bacterial polyhydroxyalkanoates. Biotechnol. Bioeng. 49 (1), 1–14. 10.1002/(SICI)1097-0290(19960105)49:1<1::AID-BIT1>3.0.CO;2-P 18623547

[B171] Lee S. Y. Kang M. S. Jeong W. Y. Han D. W. Kim K. S. (2020). Hyaluronic acid-based theranostic nanomedicines for targeted cancer therapy. Cancers 12 (4), 940. 10.3390/cancers12040940 32290285 PMC7226393

[B172] Lehmann R. Lee C. M. Shugart E. C. Benedetti M. Charo R. A. Gartner Z. (2019). Human organoids: A new dimension in cell biology. Mol. Biol. Cell 30 (10), 1129–1137. 10.1091/mbc.E19-03-0135 31034354 PMC6724519

[B173] Leroy B. P. Birch D. G. Duncan J. L. Lam B. L. Koenekoop R. K. Porto F. B. O. (2021). Leber congenital amaurosis due to Cep290 mutations-severe vision impairment with A high unmet medical need: A review. Retina 41 (5), 898–907. 10.1097/IAE.0000000000003133 33595255 PMC8078118

[B174] Li C. Du Y. Zhang T. Wang H. Hou Z. Zhang Y. (2022a). Genetic scissors" CRISPR/Cas9 genome editing cutting-edge biocarrier technology for bone and cartilage repair. Bioact. Mater. 22, 254–273. 10.1016/j.bioactmat.2022.09.026 36263098 PMC9554751

[B175] Li H. Yang Y. Hong W. (2020). Applications of genome editing technology in the targeted therapy of human diseases: Mechanisms, advances, and prospects. Sig Transduct. Target Ther. 5, 1. 10.1038/s41392-019-0089-y PMC6946647 32296011

[B176] Li J. Mooney D. J. (2016). Designing hydrogels for controlled drug delivery. Nat. Rev. Mater. 1 (12), 16071. 10.1038/natrevmats.2016.71 29657852 PMC5898614

[B177] Li L. Hu S. Chen X. (2018). Non-viral delivery systems for CRISPR/Cas9-based genome editing: Challenges and opportunities. Biomaterials 171, 207–218. 10.1016/j.biomaterials.2018.04.031 29704747 PMC5944364

[B178] Li S. Holguin L. Burnett J. C. (2022b). CRISPR-Cas9-mediated gene disruption of HIV-1 co-receptors confers broad resistance to infection in human T cells and humanized mice. Mol. Ther. - Methods & Clin. Dev. 24, 321–331. 10.1016/j.omtm.2022.01.012 35229006 PMC8847835

[B179] Li T. Yang Y. Qi H. Cui W. Zhang L. Fu X. (2023a). CRISPR/Cas9 therapeutics: Progress and prospects. Signal Transduct. Target. Ther. 8 (1), 36. 10.1038/s41392-023-01309-7 36646687 PMC9841506

[B180] Li W. Huang C. Chen J. (2022c). The application of CRISPR/Cas mediated gene editing in synthetic biology: Challenges and optimizations. Front. Bioeng. Biotechnol. 10, 890155. 10.3389/fbioe.2022.890155 36091445 PMC9452635

[B181] Li Y. Bolinger J. Yu Y. Glass Z. Shi N. Yang L. (2019). Intracellular delivery and biodistribution study of CRISPR/Cas9 ribonucleoprotein loaded bioreducible lipidoid nanoparticles. Biomaterials Sci. 7 (2), 596–606. 10.1039/c8bm00637g 30062347

[B182] Li Z. H. Wang J. Xu J. P. Wang J. Yang X. (2023b). Recent advances in CRISPR-based genome editing technology and its applications in cardiovascular research. Mil. Med. Res. 10 (1), 12. 10.1186/s40779-023-00447-x 36895064 PMC9999643

[B183] Liang D. S. Su H. T. Liu Y. J. Wang A. T. Qi X. R. (2015). Tumor-specific penetrating peptides-functionalized hyaluronic acid-d-α-tocopheryl succinate-based nanoparticles for multi-task delivery to invasive cancers. Biomaterials 71, 11–23. 10.1016/j.biomaterials.2015.08.035 26310359

[B184] Lim K. R. Q. Yoon C. Yokota T. (2018). Applications of CRISPR/Cas9 for the treatment of Duchenne muscular dystrophy. J. personalized Med. 8 (4), 38. 10.3390/jpm8040038 PMC6313657 30477208

[B185] Lin Y. Wagner E. Lächelt U. (2022). Non-viral delivery of the CRISPR/Cas system: DNA versus RNA versus RNP. Biomaterials Sci. 10 (5), 1166–1192. 10.1039/d1bm01658j 35103261

[B186] Lino C. A. Harper J. C. Carney J. P. Timlin J. A. (2018). Delivering CRISPR: A review of the challenges and approaches. Drug Deliv. 25 (1), 1234–1257. 10.1080/10717544.2018.1474964 29801422 PMC6058482

[B187] Liu B. Saber A. Haisma H. J. (2019). CRISPR/Cas9: A powerful tool for the identification of new targets for cancer treatment. Drug Discov. Today 24 (4), 955–970. 10.1016/j.drudis.2019.02.011 30849442

[B188] Liu F. Su H. Li M. Xie W. Yan Y. Shuai Q. (2022a). Zwitterionic modification of polyethyleneimine for efficient *in vitro* siRNA delivery. Int. J. Mol. Sci. 23 (9), 5014. 10.3390/ijms23095014 35563405 PMC9100541

[B189] Liu J. Yan Z. Yang F. Huang Y. Yu Y. Zhou L. (2021a). Exosomes derived from human umbilical cord mesenchymal stem cells accelerate cutaneous wound healing by enhancing angiogenesis through delivering angiopoietin-2. Stem Cell Rev. Rep. 17 (2), 305–317. 10.1007/s12015-020-09992-7 32613452

[B190] Liu S. Narancic T. Davis C. O'Connor K. E. (2022b). CRISPR/Cas9 editing of the synthesis of biodegradable polyesters polyhydroxyalkanaotes (PHA) in Pseudomonas putida KT2440. Methods Mol. Biol. Clift. N.J.) 2397, 341–358. 10.1007/978-1-0716-1826-4_17 34813072

[B191] Liu S. Yu J. M. Gan Y. C. Qiu X. Z. Gao Z. C. Wang H. (2023). Biomimetic natural biomaterials for tissue engineering and regenerative medicine: New biosynthesis methods, recent advances, and emerging applications. Mil. Med. Res. 10 (1), 16. 10.1186/s40779-023-00448-w 36978167 PMC10047482

[B192] Liu W. Li L. Jiang J. Wu M. Lin P. (2021b). Applications and challenges of CRISPR-Cas gene-editing to disease treatment in clinics. Precis. Clin. Med. 4 (3), 179–191. 10.1093/pcmedi/pbab014 34541453 PMC8444435

[B193] Liu W. Wang S. Lin B. (2021c). Applications of CRISPR/Cas9 in the research of malignant musculoskeletal tumors. BMC Musculoskelet. Disord. 22, 149. 10.1186/s12891-021-04020-2 33546657 PMC7866880

[B194] Liu Y. Wang M. Luo Y. Liang Q. Yu Y. Chen F. (2021d). Enhancing stem cell therapy for cartilage repair in osteoarthritis—a hydrogel focused approach. Gels 7 (4), 263. 10.3390/gels7040263 34940323 PMC8701810

[B195] Lu Y. Xue J. Deng T. Zhou X. Yu K. Deng L. (2020). Safety and feasibility of CRISPR-edited T cells in patients with refractory non-small-cell lung cancer. Nat. Med. 26 (5), 732–740. 10.1038/s41591-020-0840-5 32341578

[B196] Lundstrom K. (2018). Viral vectors in gene therapy. Dis. (Basel, Switz. 6 (2), 42. 10.3390/diseases6020042 PMC6023384 29883422

[B197] Lv L. Ren Y. L. Chen J. C. Wu Q. Chen G. Q. (2015). Application of CRISPRi for prokaryotic metabolic engineering involving multiple genes, a case study: Controllable P(3HB-co-4HB) biosynthesis. Metab. Eng. 29, 160–168. 10.1016/j.ymben.2015.03.013 25838211

[B198] Ma H. Marti-Gutierrez N. Park S. W. Wu J. Lee Y. Suzuki K. (2017). Correction of a pathogenic gene mutation in human embryos. Nature 548 (7668), 413–419. 10.1038/nature23305 28783728

[B199] Ma P. Mumper R. J. (2013). Paclitaxel nano-delivery systems: A comprehensive review. J. nanomedicine Nanotechnol. 4 (2), 1000164. 10.4172/2157-7439.1000164 PMC3806207 24163786

[B200] Maeder M. L. Gersbach C. A. (2016). Genome-editing technologies for gene and cell therapy. Mol. Ther. J. Am. Soc. Gene Ther. 24 (3), 430–446. 10.1038/mt.2016.10 PMC4786923 26755333

[B201] Mahmudi H. Adili-Aghdam M. A. Shahpouri M. Jaymand M. Amoozgar Z. Jahanban-Esfahlan R. (2022). Tumor microenvironment penetrating chitosan nanoparticles for the elimination of cancer relapse and minimal residual disease. Front. Oncol. 12, 1054029. 10.3389/fonc.2022.1054029 36531004 PMC9751059

[B202] Mali P. Yang L. Esvelt K. M. Aach J. Guell M. DiCarlo J. E. (2013). RNA-guided human genome engineering via Cas9. Sci. (New York, N.Y.) 339 (6121), 823–826. 10.1126/science.1232033 PMC3712628 23287722

[B203] Martinez M. G. Smekalova E. Combe E. Gregoire F. Zoulim F. Testoni B. (2022). Gene editing technologies to target HBV cccDNA. Viruses 14 (12), 2654. 10.3390/v14122654 36560658 PMC9787400

[B204] Maruyama K. (2011). Intracellular targeting delivery of liposomal drugs to solid tumors based on EPR effects. Adv. drug Deliv. Rev. 63 (3), 161–169. 10.1016/j.addr.2010.09.003 20869415

[B205] Mazurov D. Ramadan L. Kruglova N. (2023). Packaging and uncoating of CRISPR/Cas ribonucleoproteins for efficient gene editing with viral and non-viral extracellular nanoparticles. Viruses 15 (3), 690. 10.3390/v15030690 36992399 PMC10056905

[B206] McAndrews K. M. Xiao F. Chronopoulos A. LeBleu V. S. Kugeratski F. G. Kalluri R. (2021). Exosome-mediated delivery of CRISPR/Cas9 for targeting of oncogenic KrasG12D in pancreatic cancer. Life Sci. alliance 4 (9), e202000875. 10.26508/lsa.202000875 34282051 PMC8321670

[B207] McGreevy J. W. Hakim C. H. McIntosh M. A. Duan D. (2015). Animal models of Duchenne muscular dystrophy: From basic mechanisms to gene therapy. Dis. models Mech. 8 (3), 195–213. 10.1242/dmm.018424 PMC4348559 25740330

[B208] Mehta S. He T. Bajpayee A. G. (2021). Recent advances in targeted drug delivery for the treatment of osteoarthritis. Curr. Opin. Rheumatology 33 (1), 94–109. 10.1097/BOR.0000000000000761 PMC8101446 33229973

[B209] Meissner T. B. Schulze H. S. Dale S. M. (2022). Immune editing: Overcoming immune barriers in stem cell transplantation. Curr. stem Cell Rep. 8 (4), 206–218. 10.1007/s40778-022-00221-0 36406259 PMC9643905

[B210] Mendell J. R. Shieh P. B. McDonald C. M. Sahenk Z. Lehman K. J. Lowes L. P. (2023). Expression of SRP-9001 dystrophin and stabilization of motor function up to 2 years post-treatment with delandistrogene moxeparvovec gene therapy in individuals with Duchenne muscular dystrophy. Front. Cell Dev. Biol. 11, 1167762. 10.3389/fcell.2023.1167762 37497476 PMC10366687

[B211] Merienne N. Vachey G. de Longprez L. Meunier C. Zimmer V. Perriard G. (2017). The self-inactivating KamiCas9 system for the editing of CNS disease genes. Cell Rep. 20 (12), 2980–2991. 10.1016/j.celrep.2017.08.075 28930690

[B212] Min Y. L. Bassel-Duby R. Olson E. N. (2019). CRISPR correction of Duchenne muscular dystrophy. Annu. Rev. Med. 70, 239–255. 10.1146/annurev-med-081117-010451 30379597 PMC6415693

[B213] Mingozzi F. High K. A. (2017). Overcoming the host immune response to adeno-associated virus gene delivery vectors: The race between clearance, tolerance, neutralization, and escape. Annu. Rev. virology 4 (1), 511–534. 10.1146/annurev-virology-101416-041936 28961410

[B214] Mitchell M. J. Billingsley M. M. Haley R. M. Wechsler M. E. Peppas N. A. Langer R. (2021). Engineering precision nanoparticles for drug delivery. Nat. Rev. Drug Discov. 20 (2), 101–124. 10.1038/s41573-020-0090-8 33277608 PMC7717100

[B215] Mitragotri S. Lahann J. (2009). Physical approaches to biomaterial design. Nat. Mater. 8 (1), 15–23. 10.1038/nmat2344 19096389 PMC2793340

[B216] Moeini A. Pedram P. Fattahi E. Cerruti P. Santagata G. (2022). Edible polymers and secondary bioactive compounds for food packaging applications: Antimicrobial, mechanical, and gas barrier properties. Polymers 14 (12), 2395. 10.3390/polym14122395 35745971 PMC9229000

[B217] Mohammed M. A. Syeda J. T. M. Wasan K. M. Wasan E. K. (2017). An overview of chitosan nanoparticles and its application in non-parenteral drug delivery. Pharmaceutics 9 (4), 53. 10.3390/pharmaceutics9040053 29156634 PMC5750659

[B218] Moradali M. F. Rehm B. H. A. (2020). Bacterial biopolymers: From pathogenesis to advanced materials. Nat. Rev. Microbiol. 18 (4), 195–210. 10.1038/s41579-019-0313-3 31992873 PMC7223192

[B219] Musielak E. Feliczak-Guzik A. Nowak I. (2022). Synthesis and potential applications of lipid nanoparticles in medicine. Mater. (Basel, Switz. 15 (2), 682. 10.3390/ma15020682 PMC8780297 35057398

[B220] Muthu S. Bapat A. Jain R. Jeyaraman N. Jeyaraman M. (2021). Exosomal therapy new frontier in regenerative medicine. Stem Cell Investig. 8, 7. 10.21037/sci-2020-037 PMC8100822 33969112

[B221] Muzzio N. Moya S. Romero G. (2021). Multifunctional scaffolds and synergistic strategies in tissue engineering and regenerative medicine. Pharmaceutics 13 (6), 792. 10.3390/pharmaceutics13060792 34073311 PMC8230126

[B222] Nadakuduti S. S. Enciso-Rodríguez F. (2021). Advances in genome editing with CRISPR systems and transformation technologies for plant DNA manipulation. Front. plant Sci. 11, 637159. 10.3389/fpls.2020.637159 33519884 PMC7840963

[B223] Naeem M. Hoque M. Z. Ovais M. Basheer C. Ahmad I. (2021). Stimulus-Responsive smart nanoparticles-based CRISPR-cas delivery for therapeutic genome editing. Int. J. Mol. Sci. 22 (20), 11300. 10.3390/ijms222011300 34681959 PMC8540563

[B224] Naeem M. Majeed S. Hoque M. Z. Ahmad I. (2020). Latest developed strategies to minimize the off-target effects in CRISPR-cas-mediated genome editing. Cells 9 (7), 1608. 10.3390/cells9071608 32630835 PMC7407193

[B225] Naomi R. Ridzuan P. M. Bahari H. (2021). Current insights into collagen type I. Polymers 13 (16), 2642. 10.3390/polym13162642 34451183 PMC8399689

[B226] Nguyen T. P. Nguyen Q. V. Nguyen V. H. Le T. H. Huynh V. Q. N. Vo D. N. (2019). Silk fibroin-based biomaterials for biomedical applications: A review. Polymers 11 (12), 1933. 10.3390/polym11121933 31771251 PMC6960760

[B227] Nidhi S. Anand U. Oleksak P. Tripathi P. Lal J. A. Thomas G. (2021). Novel CRISPR-cas systems: An updated review of the current achievements, applications, and future research perspectives. Int. J. Mol. Sci. 22 (7), 3327. 10.3390/ijms22073327 33805113 PMC8036902

[B228] Nie D. Guo T. Yue M. Li W. Zong X. Zhu Y. (2022). Research progress on nanoparticles-based CRISPR/Cas9 system for targeted therapy of tumors. Biomolecules 12 (9), 1239. 10.3390/biom12091239 36139078 PMC9496048

[B229] Nitta S. K. Numata K. (2013). Biopolymer-based nanoparticles for drug/gene delivery and tissue engineering. Int. J. Mol. Sci. 14 (1), 1629–1654. 10.3390/ijms14011629 23344060 PMC3565338

[B230] Nsairat H. Khater D. Sayed U. Odeh F. Al Bawab A. Alshaer W. (2022). Liposomes: Structure, composition, types, and clinical applications. Heliyon 8 (5), e09394. 10.1016/j.heliyon.2022.e09394 35600452 PMC9118483

[B231] Pal K. Paulson A. T. Rousseau D. (2009). Biopolymers in controlled-release delivery systems. Mod. Biopolymer Sci. 2009, 519–557. 10.1016/b978-0-12-374195-0.00016-1

[B232] Palacios Araya D. Palmer K. L. Duerkop B. A. (2021). CRISPR-based antimicrobials to obstruct antibiotic-resistant and pathogenic bacteria. PLoS Pathog. 17 (7), e1009672. 10.1371/journal.ppat.1009672 34237097 PMC8266055

[B233] Pan X. Veroniaina H. Su N. Sha K. Jiang F. Wu Z. (2021). Applications and developments of gene therapy drug delivery systems for genetic diseases. Asian J. Pharm. Sci. 16 (6), 687–703. 10.1016/j.ajps.2021.05.003 35027949 PMC8737406

[B234] Parambi D. G. T. Alharbi K. S. Kumar R. Harilal S. Batiha G. E. Cruz-Martins N. (2022). Gene therapy approach with an emphasis on growth factors: Theoretical and clinical outcomes in neurodegenerative diseases. Mol. Neurobiol. 59 (1), 191–233. 10.1007/s12035-021-02555-y 34655056 PMC8518903

[B235] Pensado A. Seijo B. Sanchez A. (2014). Current strategies for DNA therapy based on lipid nanocarriers. Expert Opin. drug Deliv. 11 (11), 1721–1731. 10.1517/17425247.2014.935337 25046195

[B236] Piotrowski-Daspit A. S. Barone C. Lin C. Y. Deng Y. Wu D. Binns T. C. (2022). *In vivo* correction of cystic fibrosis mediated by PNA nanoparticles. Sci. Adv. 8 (40), eabo0522. 10.1126/sciadv.abo0522 36197984 PMC9534507

[B237] Pirota V. Bisbano G. Serra M. Torre M. L. Doria F. Bari E. (2023). cRGD-functionalized silk fibroin nanoparticles: A strategy for cancer treatment with a potent unselective naphthalene diimide derivative. Cancers 15 (6), 1725. 10.3390/cancers15061725 36980611 PMC10046852

[B238] Prakash P. Lee W. H. Loo C. Y. Wong H. S. J. Parumasivam T. (2022). Advances in polyhydroxyalkanoate nanocarriers for effective drug delivery: An overview and challenges. Nanomater. (Basel, Switz. 12 (1), 175. 10.3390/nano12010175v PMC8746483 35010124

[B239] Prodinger C. Reichelt J. Bauer J. W. Laimer M. (2019). Epidermolysis bullosa: Advances in research and treatment. Exp. Dermatol. 28 (10), 1176–1189. 10.1111/exd.13979 31140655 PMC6900197

[B240] Pulingam T. Appaturi J. N. Parumasivam T. Ahmad A. Sudesh K. (2022). Biomedical applications of polyhydroxyalkanoate in tissue engineering. Polymers 14 (11), 2141. 10.3390/polym14112141 35683815 PMC9182786

[B241] Qi Y. Liu Y. Yu B. Hu Y. Zhang N. Zheng Y. (2020). A lactose-derived CRISPR/Cas9 delivery system for efficient genome editing *in vivo* to treat orthotopic hepatocellular carcinoma. Adv. Sci. (Weinheim, Baden-Wurttemberg, Ger. 7 (17), 2001424. 10.1002/advs.202001424 PMC7507475 32995132

[B242] Qiao J. Sun W. Lin S. Jin R. Ma L. Liu Y. (2019). Cytosolic delivery of CRISPR/Cas9 ribonucleoproteins for genome editing using chitosan-coated red fluorescent protein. Chem. Commun. 55 (32), 4707–4710. 10.1039/c9cc00010k 30942216

[B243] Qin Q. Ling C. Zhao Y. Yang T. Yin J. Guo Y. (2018). CRISPR/Cas9 editing genome of extremophile Halomonas spp. Metab. Eng. 47, 219–229. 10.1016/j.ymben.2018.03.018 29609045

[B244] Qiu C. Wei W. Sun J. Zhang H.-T. Ding J.-S. Wang J.-C. (2016). Systemic delivery of siRNA by hyaluronan-functionalized calcium phosphate nanoparticles for tumor-targeted therapy. Nanoscale 8 (26), 13033–13044. 10.1039/c6nr04034a 27314204

[B245] Qiu M. Glass Z. Chen J. Haas M. Jin X. Zhao X. (2021). Lipid nanoparticle-mediated codelivery of Cas9 mRNA and single-guide RNA achieves liver-specific *in vivo* genome editing of Angptl3. Proc. Natl. Acad. Sci. U. S. A. 118 (10), e2020401118. 10.1073/pnas.2020401118 33649229 PMC7958351

[B246] Rafii S. Tashkandi E. Bukhari N. Al-Shamsi H. O. (2022). Current status of CRISPR/Cas9 application in clinical cancer research: Opportunities and challenges. Cancers 14 (4), 947. 10.3390/cancers14040947 35205694 PMC8870204

[B247] Rajput A. Varshney A. Bajaj R. Pokharkar V. (2022). Exosomes as new generation vehicles for drug delivery: Biomedical applications and future perspectives. Mol. (Basel, Switz. 27 (21), 7289. 10.3390/molecules27217289 PMC9658823 36364116

[B248] Ramamoorth M. Narvekar A. (2015). Non-viral vectors in gene therapy-an overview. J. Clin. diagnostic Res. JCDR 9 (1), GE01. 10.7860/JCDR/2015/10443.5394 PMC4347098 25738007

[B249] Ran F. A. Hsu P. D. Wright J. Agarwala V. Scott D. A. Zhang F. (2013). Genome engineering using the CRISPR-Cas9 system. Nat. Protoc. 8 (11), 2281–2308. 10.1038/nprot.2013.143 24157548 PMC3969860

[B250] Rasul M. F. Hussen B. M. Salihi A. Ismael B. S. Jalal P. J. Zanichelli A. (2022). Strategies to overcome the main challenges of the use of CRISPR/Cas9 as a replacement for cancer therapy. Mol. cancer 21 (1), 64. 10.1186/s12943-021-01487-4 35241090 PMC8892709

[B251] Ray S. Kalia V. C. (2017). Biomedical applications of polyhydroxyalkanoates. Indian J. Microbiol. 57 (3), 261–269. 10.1007/s12088-017-0651-7 28904409 PMC5574769

[B252] Rezvantalab S. Drude N. I. Moraveji M. K. Güvener N. Koons E. K. Shi Y. (2018). PLGA-based nanoparticles in cancer treatment. Front. Pharmacol. 9, 1260. 10.3389/fphar.2018.01260 30450050 PMC6224484

[B253] Riaz S. Rhee K. Y. Park S. J. (2021). Polyhydroxyalkanoates (PHAs): Biopolymers for biofuel and biorefineries. Polymers 13 (2), 253. 10.3390/polym13020253 33451137 PMC7828617

[B254] Riley M. K. Vermerris W. (2017). Recent advances in nanomaterials for gene delivery-A review. Nanomater. (Basel, Switz. 7 (5), 94. 10.3390/nano7050094 PMC5449975 28452950

[B255] Robubi A. Berger C. Schmid M. Huber K. R. Engel A. Krugluger W. (2014). Gene expression profiles induced by growth factors *in vitro* cultured osteoblasts. Bone & Jt. Res. 3 (7), 236–240. 10.1302/2046-3758.37.2000231 PMC4112778 25057185

[B256] Rodríguez-Rodríguez D. R. Ramírez-Solís R. Garza-Elizondo M. A. Garza-Rodríguez M. L. Barrera-Saldaña H. A. (2019). Genome editing: A perspective on the application of CRISPR/cas9 to study human diseases (review). Int. J. Mol. Med. 43 (4), 1559–1574. 10.3892/ijmm.2019.4112 30816503 PMC6414166

[B257] Rohiwal S. S. Dvorakova N. Klima J. Vaskovicova M. Senigl F. Slouf M. (2020). Polyethylenimine-based magnetic nanoparticles mediated non-viral CRISPR/Cas9 system for genome editing. Sci. Rep. 10 (1), 4619. 10.1038/s41598-020-61465-6 32165679 PMC7067791

[B258] Rosenblum D. Gutkin A. Kedmi R. Ramishetti S. Veiga N. Jacobi A. M. (2020). CRISPR/Cas9 genome editing using targeted lipid nanoparticles for cancer therapy. Sci. Adv. 6 (47), eabc9450. 10.1126/sciadv.abc9450 33208369 PMC7673804

[B259] Rouatbi N. McGlynn T. Al-Jamal K. T. (2022). Pre-clinical non-viral vectors exploited for *in vivo* CRISPR/Cas9 gene editing: An overview. Biomaterials Sci. 10 (13), 3410–3432. 10.1039/d1bm01452h 35604372

[B260] Rouet R. Thuma B. A. Roy M. D. Lintner N. G. Rubitski D. M. Finley J. E. (2018). Receptor-mediated delivery of CRISPR/Cas9 endonuclease for cell-type-specific gene editing. J. Am. Chem. Soc. 140 (21), 6596–6603. 10.1021/jacs.8b01551 29668265 PMC6002863

[B261] Roy H. Brahma C. K. Nandi S. Parida K. R. (2013). Formulation and design of sustained release matrix tablets of metformin hydrochloride: Influence of hypromellose and polyacrylate polymers. Int. J. Appl. basic Med. Res. 3 (1), 55–63. 10.4103/2229-516X.112242 23776841 PMC3678683

[B262] Ryczek N. Hryhorowicz M. Zeyland J. Lipiński D. Słomski R. (2021). CRISPR/Cas technology in pig-to-human xenotransplantation research. Int. J. Mol. Sci. 22 (6), 3196. 10.3390/ijms22063196 33801123 PMC8004187

[B263] Ryu N. Kim M. A. Park D. Lee B. Kim Y. R. Kim K. H. (2018). Effective PEI-mediated delivery of CRISPR/Cas9 complex for targeted gene therapy. Nanomedicine Nanotechnol. Biol. Med. 14 (7), 2095–2102. 10.1016/j.nano.2018.06.009 29969727

[B264] Sahel D. K. Vora L. K. Saraswat A. Sharma S. Monpara J. D'Souza A. A. (2023). CRISPR/Cas9 genome editing for tissue-specific *in vivo* targeting: Nanomaterials and translational perspective. Adv. Sci. 2023, e2207512. 10.1002/advs.202207512 PMC10323670 37166046

[B265] Sahu I. Haque A. K. M. A. Weidensee B. Weinmann P. Kormann M. S. D. (2019). Recent developments in mRNA-based protein supplementation therapy to target lung diseases. Mol. Ther. J. Am. Soc. Gene Ther. 27 (4), 803–823. 10.1016/j.ymthe.2019.02.019 PMC6453549 30905577

[B266] Salata O. (2004). Applications of nanoparticles in biology and medicine. J. Nanobiotechnology 2 (1), 3. 10.1186/1477-3155-2-3 15119954 PMC419715

[B267] Salman A. Kantor A. McClements M. E. Marfany G. Trigueros S. MacLaren R. E. (2022). Non-viral delivery of CRISPR/Cas cargo to the retina using nanoparticles: Current possibilities, challenges, and limitations. Pharmaceutics 14 (9), 1842. 10.3390/pharmaceutics14091842 36145593 PMC9503525

[B268] Saw P. E. Cui G. H. Xu X. (2022). Nanoparticles-mediated CRISPR/Cas gene editing delivery system. ChemMedChem 17 (9), e202100777. 10.1002/cmdc.202100777 35261159

[B269] Seyhan A. A. Carini C. (2019). Are innovation and new technologies in precision medicine paving a new era in patient-centric care? J. Transl. Med. 17, 114. 10.1186/s12967-019-1864-9 30953518 PMC6451233

[B270] Shabbir M. A. B. Shabbir M. Z. Wu Q. Mahmood S. Sajid A. Maan M. K. (2019). CRISPR-cas system: Biological function in microbes and its use to treat antimicrobial resistant pathogens. Ann. Clin. Microbiol. Antimicrob. 18 (1), 21. 10.1186/s12941-019-0317-x 31277669 PMC6611046

[B271] Sharma G. Sharma A. R. Bhattacharya M. Lee S. S. Chakraborty C. (2021). CRISPR/Cas9: A preclinical and clinical perspective for the treatment of human diseases. Mol. Ther. J. Am. Soc. Gene Ther. 29 (2), 571–586. 10.1016/j.ymthe.2020.09.028 PMC7854284 33238136

[B272] Shirley J. L. de Jong Y. P. Terhorst C. Herzog R. W. (2020). Immune responses to viral gene therapy vectors. Mol. Ther. J. Am. Soc. Gene Ther. 28 (3), 709–722. 10.1016/j.ymthe.2020.01.001 PMC7054714 31968213

[B273] Shrivastav A. Kim H. Y. Kim Y. R. (2013). Advances in the applications of polyhydroxyalkanoate nanoparticles for novel drug delivery systems. BioMed Res. Int. 2013, 581684. 10.1155/2013/581684 23984383 PMC3741897

[B274] Shtykalova S. Deviatkin D. Freund S. Egorova A. Kiselev A. (2023). Non-viral carriers for nucleic acids delivery: Fundamentals and current applications. Life 13 (4), 903. 10.3390/life13040903 37109432 PMC10142071

[B275] Šimić G. (2019). Rare diseases and omics-driven personalized medicine. Croat. Med. J. 60 (6), 485–487. 10.3325/cmj.2019.60.485 31894912 PMC6952900

[B276] Sinclair F. Begum A. A. Dai C. C. Toth I. Moyle P. M. (2023). Recent advances in the delivery and applications of nonviral CRISPR/Cas9 gene editing. Drug Deliv. Transl. Res. 13 (5), 1500–1519. 10.1007/s13346-023-01320-z 36988873 PMC10052255

[B277] Song R. Murphy M. Li C. Ting K. Soo C. Zheng Z. (2018). Current development of biodegradable polymeric materials for biomedical applications. Drug Des. Dev. Ther. 12, 3117–3145. 10.2147/DDDT.S165440 PMC6161720 30288019

[B278] Srifa W. Kosaric N. Amorin A. Jadi O. Park Y. Mantri S. (2020). Cas9-AAV6-engineered human mesenchymal stromal cells improved cutaneous wound healing in diabetic mice. Nat. Commun. 11 (1), 2470. 10.1038/s41467-020-16065-3 32424320 PMC7235221

[B279] Srivastava A. Mallela K. Deorkar N. Brophy G. (2021). Manufacturing challenges and rational formulation development for AAV viral vectors. J. Pharm. Sci. 110 (7), 2609–2624. 10.1016/j.xphs.2021.03.024 33812887

[B280] Sufian M. A. Ilies M. A. (2023). Lipid-based nucleic acid therapeutics with *in vivo* efficacy. Wiley Interdiscip. Rev. Nanomedicine nanobiotechnology 15 (2), e1856. 10.1002/wnan.1856 36180107 PMC10023279

[B281] Sun T. Zhang Y. S. Pang B. Hyun D. C. Yang M. Xia Y. (2014). Engineered nanoparticles for drug delivery in cancer therapy. Angewandte Chemie Int. ed. Engl. 53 (46), 12320–12364. 10.1002/anie.201403036 25294565

[B282] Sundar S. Kundu J. Kundu S. C. (2010). Biopolymeric nanoparticles. Sci. Technol. Adv. Mater. 11 (1), 014104. 10.1088/1468-6996/11/1/014104 27877319 PMC5090546

[B283] Swetha K. Kotla N. G. Tunki L. Jayaraj A. Bhargava S. K. Hu H. (2023). Recent advances in the lipid nanoparticle-mediated delivery of mRNA vaccines. Vaccines 11 (3), 658. 10.3390/vaccines11030658 36992242 PMC10059764

[B284] Syaharani R. Adhima F. Nia Amru S. I'tishom R. (2021). Lipid nanoparticles delivery of CRISPR/Cas9 targeting PCSK9 and ANGTPL3 as new therapeutic gene editing modalities for potential long-lasting treatment of dyslipidemia. Int. J. Res. Publ. 92 (1). 10.47119/ijrp100921120222750

[B285] Sydnor E. R. Perl T. M. (2011). Hospital epidemiology and infection control in acute-care settings. Clin. Microbiol. Rev. 24 (1), 141–173. 10.1128/CMR.00027-10 21233510 PMC3021207

[B286] Taha E. A. Lee J. Hotta A. (2022). Delivery of CRISPR-Cas tools for *in vivo* genome editing therapy: Trends and challenges. J. Control. release official J. Control. Release Soc. 342, 345–361. 10.1016/j.jconrel.2022.01.013 35026352

[B287] Tang Q. Liu J. Jiang Y. Zhang M. Mao L. Wang M. (2019). Cell-selective messenger RNA delivery and CRISPR/Cas9 genome editing by modulating the interface of phenylboronic acid-derived lipid nanoparticles and cellular surface sialic acid. ACS Appl. Mater. Interfaces 11 (50), 46585–46590. 10.1021/acsami.9b17749 31763806

[B288] Tao S. Chen H. Li N. Liang W. (2022). The application of the CRISPR-cas system in antibiotic resistance. Infect. drug Resist. 15, 4155–4168. 10.2147/IDR.S370869 35942309 PMC9356603

[B289] Tavakoli K. Pour-Aboughadareh A. Kianersi F. Poczai P. Etminan A. Shooshtari L. (2021). Applications of CRISPR-cas9 as an advanced genome editing system in life sciences. Biotech. (Basel Switz. 10 (3), 14. 10.3390/biotech10030014 PMC9245484 35822768

[B290] Thein-Han W. W. Saikhun J. Pholpramoo C. Misra R. D. Kitiyanant Y. (2009). Chitosan-gelatin scaffolds for tissue engineering: Physicochemical properties and biological response of buffalo embryonic stem cells and transfectant of GFP-buffalo embryonic stem cells. Acta biomater. 5 (9), 3453–3466. 10.1016/j.actbio.2009.05.012 19460465

[B291] Tomić S. Lj. Babić Radić M. M. Vuković J. S. Filipović V. V. Nikodinovic-Runic J. Vukomanović M. (2023). Alginate-based hydrogels and scaffolds for biomedical applications. Mar. Drugs 21 (3), 177. 10.3390/md21030177 36976226 PMC10055882

[B292] Tuder R. M. Abman S. H. Braun T. Capron F. Stevens T. Thistlethwaite P. A. (2009). Development and pathology of pulmonary hypertension. J. Am. Coll. Cardiol. 54 (1), S3. 10.1016/j.jacc.2009.04.009 19555856

[B293] Tycko J. Myer V. E. Hsu P. D. (2016). Methods for optimizing CRISPR-cas9 genome editing specificity. Mol. Cell 63 (3), 355–370. 10.1016/j.molcel.2016.07.004 27494557 PMC4976696

[B294] Uddin F. Rudin C. M. Sen T. (2020). CRISPR gene therapy: Applications, limitations, and implications for the future. Front. Oncol. 10, 1387. 10.3389/fonc.2020.01387 32850447 PMC7427626

[B295] Uribe R. V. Rathmer C. Jahn L. J. Ellabaan M. M. H. Li S. S. Sommer M. O. A. (2021). Bacterial resistance to CRISPR-Cas antimicrobials. Sci. Rep. 11 (1), 17267. 10.1038/s41598-021-96735-4 34446818 PMC8390487

[B296] Vaghari-Tabari M. Hassanpour P. Sadeghsoltani F. Malakoti F. Alemi F. Qujeq D. (2022). CRISPR/Cas9 gene editing: A new approach for overcoming drug resistance in cancer. Cell. Mol. Biol. Lett. 27 (1), 49. 10.1186/s11658-022-00348-2 35715750 PMC9204876

[B297] Valenti M. T. Serena M. Carbonare L. D. Zipeto D. (2019). CRISPR/Cas system: An emerging technology in stem cell research. World J. Stem Cells 11 (11), 937–956. 10.4252/wjsc.v11.i11.937 31768221 PMC6851009

[B298] Vargason A. M. Anselmo A. C. Mitragotri S. (2021). The evolution of commercial drug delivery technologies. Nat. Biomed. Eng. 5 (9), 951–967. 10.1038/s41551-021-00698-w 33795852

[B299] Vauthier C. Zandanel C. Ramon A. L. (2013). Chitosan-based nanoparticles for *in vivo* delivery of interfering agents including siRNA. Curr. Opin. Colloid Interface Sci. 18 (5), 406–418. 10.1016/j.cocis.2013.06.005

[B300] Wadher K. J. Kakde R. B. Umekar M. J. (2011). Study on sustained-release metformin hydrochloride from matrix tablet: Influence of hydrophilic polymers and *in vitro* evaluation. Int. J. Pharm. investigation 1 (3), 157–163. 10.4103/2230-973X.85966 PMC3465141 23071938

[B301] Wan T. Ping Y. (2021). Delivery of genome-editing biomacromolecules for treatment of lung genetic disorders. Adv. drug Deliv. Rev. 168, 196–216. 10.1016/j.addr.2020.05.002 32416111

[B302] Wan T. Zhong J. Pan Q. Zhou T. Ping Y. Liu X. (2022). Exosome-mediated delivery of Cas9 ribonucleoprotein complexes for tissue-specific gene therapy of liver diseases. Sci. Adv. 8 (37), eabp9435. 10.1126/sciadv.abp9435 36103526 PMC9473578

[B303] Wang C. Fang S. Chen Y. Tang N. Jiao G. Hu Y. (2023). High-efficiency targeted transgene integration via primed micro-homologs. Cell Discov. 9 (1), 69. 10.1038/s41421-023-00552-0 37402729 PMC10319781

[B304] Wang L. Chen Y. Liu X. Li Z. Dai X. (2022). The application of CRISPR/Cas9 technology for cancer immunotherapy: Current status and problems. Front. Oncol. 11, 704999. 10.3389/fonc.2021.704999 35111663 PMC8801488

[B305] Wang L. Jia E. (2016). Ovarian cancer targeted hyaluronic acid-based nanoparticle system for paclitaxel delivery to overcome drug resistance. Drug Deliv. 23 (5), 1810–1817. 10.3109/10717544.2015.1101792 26530693

[B306] Wang M. Zuris J. A. Meng F. Rees H. Sun S. Deng P. (2016). Efficient delivery of genome-editing proteins using bioreducible lipid nanoparticles. Proc. Natl. Acad. Sci. U. S. A. 113 (11), 2868–2873. 10.1073/pnas.1520244113 26929348 PMC4801296

[B307] Wang Y. Tang Y. Zhao X. M. Huang G. Gong J. H. Yang S. D. (2022). A multifunctional non-viral vector for the delivery of MTH1-targeted CRISPR/Cas9 system for non-small cell lung cancer therapy. Acta biomater. 153, 481–493. 10.1016/j.actbio.2022.09.046 36162766

[B308] Wei T. Cheng Q. Farbiak L. Anderson D. G. Langer R. Siegwart D. J. (2020). Delivery of tissue-targeted scalpels: Opportunities and challenges for *in vivo* CRISPR/Cas-Based genome editing. ACS Nano 14 (8), 9243–9262. 10.1021/acsnano.0c04707 32697075 PMC7996671

[B309] Wei W. Xin H. Roy B. Dai J. Miao Y. Gao G. (2014). Heritable genome editing with CRISPR/Cas9 in the silkworm, *Bombyx mori* . PLoS ONE 9 (7), e101210. 10.1371/journal.pone.0101210 25013902 PMC4094479

[B310] Westermann L. Neubauer B. Köttgen M. (2021). Nobel prize 2020 in chemistry honors CRISPR: A tool for rewriting the code of life. Pflugers Archiv Eur. J. physiology 473 (1), 1–2. 10.1007/s00424-020-02497-9 33244639 PMC7782372

[B311] Whatley M. Francis A. Ng Z. Y. Khoh X. E. Atlas M. D. Dilley R. J. (2020). Usher syndrome: Genetics and molecular links of hearing loss and directions for therapy. Front. Genet. 11, 565216. 10.3389/fgene.2020.565216 33193648 PMC7642844

[B312] Wilbie D. Walther J. Mastrobattista E. (2019). Delivery aspects of CRISPR/Cas for *in vivo* genome editing. Accounts Chem. Res. 52 (6), 1555–1564. 10.1021/acs.accounts.9b00106 PMC6584901 31099553

[B313] Won E. J. Park H. Chang S. H. Kim J. H. Kwon H. Cho Y. S. (2021). One-shot dual gene editing for drug-resistant pancreatic cancer therapy. Biomaterials 279, 121252. 10.1016/j.biomaterials.2021.121252 34781244

[B314] Wong K. H. Lu A. Chen X. Yang Z. (2020). Natural ingredient-based polymeric nanoparticles for cancer treatment. Molecules 25 (16), 3620. 10.3390/molecules25163620 32784890 PMC7463484

[B315] Wu W. Lu Z. Li F. Wang W. Qian N. Duan J. (2017). Efficient *in vivo* gene editing using ribonucleoproteins in skin stem cells of recessive dystrophic epidermolysis bullosa mouse model. Proc. Natl. Acad. Sci. U. S. A. 114 (7), 1660–1665. 10.1073/pnas.1614775114 28137859 PMC5321012

[B316] Xavier M. Farez N. Salvatierra P. L. Jardini A. L. Kharmandayan P. Feldman S. (2021). Biological performance of a bioabsorbable poly (L-Lactic acid) produced in polymerization unit: *In vivo* studies. F1000Research 10, 1275. 10.12688/f1000research.73754.1 35035900 PMC8729025

[B317] Xi S. Yang Y. G. Suo J. Sun T. (2022). Research progress on gene editing based on nano-drug delivery vectors for tumor therapy. Front. Bioeng. Biotechnol. 10, 873369. 10.3389/fbioe.2022.873369 35419357 PMC8996155

[B318] Xia W. Tao Z. Zhu B. Zhang W. Liu C. Chen S. (2021). Targeted delivery of drugs and genes using polymer nanocarriers for cancer therapy. Int. J. Mol. Sci. 22 (17), 9118. 10.3390/ijms22179118 34502028 PMC8431379

[B319] Xie J. Michael P. L. Zhong S. Ma B. MacEwan M. R. Lim C. T. (2012). Mussel-inspired protein-mediated surface modification to electrospun fibers and their potential biomedical applications. J. Biomed. Mater. Res. Part A 100 (4), 929–938. 10.1002/jbm.a.34030 22275174

[B320] Xu C. L. Ruan M. Mahajan V. B. Tsang S. H. (2019a). Viral delivery systems for CRISPR. Viruses 11 (1), 28. 10.3390/v11010028 30621179 PMC6356701

[B321] Xu H. Niu M. Yuan X. Wu K. Liu A. (2020a). CD44 as a tumor biomarker and therapeutic target. Exp. Hematol. Oncol. 9 (1), 36. 10.1186/s40164-020-00192-0 33303029 PMC7727191

[B322] Xu T. Li L. Liu Y. C. Cao W. Chen J. S. Hu S. (2020b). CRISPR/Cas9-related technologies in liver diseases: From feasibility to future diversity. Int. J. Biol. Sci. 16 (13), 2283–2295. 10.7150/ijbs.33481 32760197 PMC7378651

[B323] Xu W. Song W. Yang Y. Wu Y. Lv X. Yuan S. (2019b). Multiplex nucleotide editing by high-fidelity Cas9 variants with improved efficiency in rice. BMC Plant Biol. 19 (1), 511. 10.1186/s12870-019-2131-1 31752697 PMC6873407

[B324] Xu X. Liu C. Wang Y. Koivisto O. Zhou J. Shu Y. (2021). Nanotechnology-based delivery of CRISPR/Cas9 for cancer treatment. Adv. drug Deliv. Rev. 176, 113891. 10.1016/j.addr.2021.113891 34324887

[B325] Xu X. Xia T. (2023). Recent advances in site-specific lipid nanoparticles for mRNA delivery. ACS Nanosci. Au 3 (3), 192–203. 10.1021/acsnanoscienceau.2c00062 37360845 PMC10288611

[B326] Xu Y. Li Z. (2020). CRISPR-Cas systems: Overview, innovations, and applications in human disease research and gene therapy. Comput. Struct. Biotechnol. J. 18, 2401–2415. 10.1016/j.csbj.2020.08.031 33005303 PMC7508700

[B327] Xu Z. Wang Q. Zhong H. Jiang Y. Shi X. Yuan B. (2022). Carrier strategies boost the application of the CRISPR/Cas system in gene therapy. Explor. (Beijing, China) 2 (2), 20210081. 10.1002/EXP.20210081 PMC10190933 37323878

[B328] Yang B. Li G. Liu J. Li X. Zhang S. Sun F. (2021). Nanotechnology for age-related macular degeneration. Pharmaceutics 13 (12), 2035. 10.3390/pharmaceutics13122035 34959316 PMC8705006

[B329] Yang K. Qian J. Zhang C. Wang Z. Huang Q. Shi G. (2023). Biogenic materials for CRISPR delivery and therapeutics. Biomaterials Sci. 11 (9), 3016–3033. 10.1039/d2bm02169b 36897609

[B330] Yin H. Kauffman K. J. Anderson D. G. (2017). Delivery technologies for genome editing. Nat. Rev. Drug Discov. 16 (6), 387–399. 10.1038/nrd.2016.280 28337020

[B331] Yin X. Mead B. E. Safaee H. Langer R. Karp J. M. Levy O. (2016). Engineering stem cell organoids. Cell stem Cell 18 (1), 25–38. 10.1016/j.stem.2015.12.005 26748754 PMC4728053

[B332] Yip B. H. (2020). Recent advances in CRISPR/Cas9 delivery strategies. Biomolecules 10 (6), 839. 10.3390/biom10060839 32486234 PMC7356196

[B333] Young C. S. Hicks M. R. Ermolova N. V. Nakano H. Jan M. Younesi S. (2016). A single CRISPR/Cas9 deletion strategy that targets the majority of DMD patients restores dystrophin function in hiPSC-derived muscle cells. Cell stem Cell 18 (4), 533–540. 10.1016/j.stem.2016.01.021 26877224 PMC4826286

[B334] Yu B. Li Y. Lin Y. Zhu Y. Hao T. Wu Y. (2023a). Research progress of natural silk fibroin and its application for drug delivery in chemotherapies. Front. Pharmacol. 13, 1071868. 10.3389/fphar.2022.1071868 36686706 PMC9845586

[B335] Yu Y. Gao Y. He L. Fang B. Ge W. Yang P. (2023b). Biomaterial-based gene therapy. MedComm 4 (3), e259. 10.1002/mco2.259 37284583 PMC10239531

[B336] Yucel T. Lovett M. L. Kaplan D. L. (2014). Silk-based biomaterials for sustained drug delivery. J. Control. release official J. Control. Release Soc. 190, 381–397. 10.1016/j.jconrel.2014.05.059 PMC4142080 24910193

[B337] Yun Y. R. Won J. E. Jeon E. Lee S. Kang W. Jo H. (2010). Fibroblast growth factors: Biology, function, and application for tissue regeneration. J. tissue Eng. 2010, 218142. 10.4061/2010/218142 21350642 PMC3042641

[B338] Zhang F. Zhang C. Fu S. Liu H. Han M. Fan X. (2022). Amphiphilic cationic peptide-coated PHA nanosphere as an efficient vector for multiple-drug delivery. Nanomater. (Basel, Switz. 12 (17), 3024. 10.3390/nano12173024 PMC9457696 36080060

[B339] Zhang H. Bahamondez-Canas T. F. Zhang Y. Leal J. Smyth H. D. C. (2018). PEGylated chitosan for nonviral aerosol and mucosal delivery of the CRISPR/Cas9 system *in vitro* . Mol. Pharm. 15 (11), 4814–4826. 10.1021/acs.molpharmaceut.8b00434 30222933 PMC6453125

[B340] Zhang H. Cheng J. Ao Q. (2021a). Preparation of alginate-based biomaterials and their applications in biomedicine. Mar. drugs 19 (5), 264. 10.3390/md19050264 34068547 PMC8150954

[B341] Zhang S. Shen J. Li D. Cheng Y. (2021b). Strategies in the delivery of Cas9 ribonucleoprotein for CRISPR/Cas9 genome editing. Theranostics 11 (2), 614–648. 10.7150/thno.47007 33391496 PMC7738854

[B342] Zhang X. H. Tee L. Y. Wang X. G. Huang Q. S. Yang S. H. (2015). Off-target effects in CRISPR/Cas9-mediated genome engineering. Molecular therapy. Nucleic acids. 4 (11), e264. 10.1038/mtna.2015.37 26575098 PMC4877446

[B343] Zhang X. Lei T. Du H. (2021c). The prospect of cell-penetrating peptides in stem cell tracking. Stem Cell Res. Ther. 12 (1), 457. 10.1186/s13287-021-02522-3 34391472 PMC8364034

[B344] Zhang Y. Bassel-Duby R. Olson E. N. (2023). CRISPR/Cas9 correction of Duchenne muscular dystrophy in mice by a self-complementary AAV delivery system. Methods Mol. Biol. Clift. N.J.) 2587, 411–425. 10.1007/978-1-0716-2772-3_21 PMC10069557 36401041

[B345] Zhang Y. Sun C. Wang C. Jankovic K. E. Dong Y. (2021d). Lipids and lipid derivatives for RNA delivery. Chem. Rev. 121 (20), 12181–12277. 10.1021/acs.chemrev.1c00244 34279087 PMC10088400

[B346] Zhang Y. Zhang Z. (2020). The history and advances in cancer immunotherapy: Understanding the characteristics of tumor-infiltrating immune cells and their therapeutic implications. Cell. Mol. Immunol. 17 (8), 807–821. 10.1038/s41423-020-0488-6 32612154 PMC7395159

[B347] Zhang Z. Zhang Y. Gao F. Han S. Cheah K. S. Tse H. F. (2017). CRISPR/Cas9 genome-editing system in human stem cells: Current status and future prospects. Molecular therapy. Nucleic acids. 9, 230–241. 10.1016/j.omtn.2017.09.009 29246302 PMC5651489

[B348] Zhao H. Li Z. Wang Y. Zhou K. Li H. Bi S. (2023). Bioengineered MSC-derived exosomes in skin wound repair and regeneration. Front. Cell Dev. Biol. 11, 1029671. 10.3389/fcell.2023.1029671 36923255 PMC10009159

[B349] Zhao Z. Li Y. Xie M.-B. (2015). Silk fibroin-based nanoparticles for drug delivery. Int. J. Mol. Sci. 16 (3), 4880–4903. 10.3390/ijms16034880 25749470 PMC4394455

[B350] Zhou H. He Y. Xiong W. Jing S. Duan X. Huang Z. (2022). MSC-based gene delivery methods and strategies improve the therapeutic efficacy of neurological diseases. Bioact. Mater. 23, 409–437. 10.1016/j.bioactmat.2022.11.007 36474656 PMC9713256

[B351] Zhou Z. P. Yang L. L. Cao H. Chen Z. R. Zhang Y. Wen X. Y. (2019). *In vitro* validation of a CRISPR-mediated CFTR correction strategy for preclinical translation in pigs. Hum. gene Ther. 30 (9), 1101–1116. 10.1089/hum.2019.074 31099266

[B352] Zhu X. Gao M. Yang Y. Li W. Bao J. Li Y. (2023). The CRISPR/Cas9 system delivered by extracellular vesicles. Pharmaceutics 15 (3), 984. 10.3390/pharmaceutics15030984 36986843 PMC10053467

[B353] Zhuo C. Zhang J. Lee J. H. Jiao J. Cheng D. Liu L. (2021). Spatiotemporal control of CRISPR/Cas9 gene editing. Signal Transduct. Target. Ther. 6 (1), 238. 10.1038/s41392-021-00645-w 34148061 PMC8214627

[B354] Zou Y. Sun X. Yang Q. Zheng M. Shimoni O. Ruan W. (2022). Blood-brain barrier-penetrating single CRISPR-Cas9 nanocapsules for effective and safe glioblastoma gene therapy. Sci. Adv. 8 (16), eabm8011. 10.1126/sciadv.abm8011 35442747 PMC9020780

[B355] Zuris J. A. Thompson D. B. Shu Y. Guilinger J. P. Bessen J. L. Hu J. H. (2015). Cationic lipid-mediated delivery of proteins enables efficient protein-based genome editing *in vitro* and *in vivo* . Nat. Biotechnol. 33 (1), 73–80. 10.1038/nbt.3081 25357182 PMC4289409

